# Beyond Hydrogen Bonding: π···π Stacking Directed Self‐Assembly of Carboxylic Acid Clusters in the Gas Phase

**DOI:** 10.1002/anie.202523854

**Published:** 2026-02-17

**Authors:** Jingling Hong, Melanie Schnell, Mingfei Zhou, Weixing Li

**Affiliations:** ^1^ Department of Chemistry, State Key Laboratory of Porous Materials for Separation and Conversion, Shanghai Key Laboratory of Molecular Catalysis and Innovative Materials Fudan University Shanghai China; ^2^ Deutsches Elektronen‐Synchrotron DESY Hamburg Germany; ^3^ Institut für Physikalische Chemie Christian‐Albrechts‐Universität Zu Kiel Kiel Germany

**Keywords:** carboxylic acid, hydrogen bonding, π–π stacking, molecular aggregation, rotational spectroscopy

## Abstract

Carboxylic acids critically influence atmospheric chemistry by modulating acidity, new particle formation, and aerosol growth. The hydrogen‐bonding capability of the −COOH group drives the assembly of structurally diverse gas‐phase clusters with atmospheric species. Despite their importance, experimental data on larger carboxylic acid clusters remain limited, and computational predictions of their global minimum structures lack consensus. Here, we employ high‐resolution microwave spectroscopy to determine the geometries of formic acid and propiolic acid clusters, identifying three trimers and two tetramers. A total of 34 isotopologues were analyzed to robustly confirm the cluster structures. Symmetry‐adapted perturbation theory and many‐body expansion analyses demonstrate a fundamental transition in stabilization mechanisms: trimers rely on conventional hydrogen bonds, whereas tetramers exhibit cooperative π–π stacking interactions that drive a structural transformation from single‐layer to double‐layer architectures. These findings resolve long‐standing ambiguities in cluster configurations and establish essential benchmarks for modeling carboxylic acid‐driven atmospheric nucleation processes.

## Graphical abstract

A critical transformation from hydrogen‐bonded trimers to π–π stacked tetramers is unveiled by microwave spectroscopy, highlighting dispersion as a key force in directing carboxylic acid aggregation.

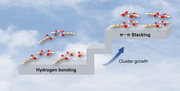

## 1 Introduction

Carboxylic acids are one of the dominant classes of organic compounds in the atmosphere and play a crucial role in precipitation acidity [1, 2, 3, 4]. Due to their extensive sources, including both direct emissions from anthropogenic and biogenic activities and the photochemical oxidation of precursor organic species, their atmospheric concentrations are expected to surpass those of inorganic acids, such as sulfuric acid [5, 6]. They have been shown to contribute significantly to new particle formation and aerosol growth as they can form large and stable clusters with other nucleating components [7, 8, 9, 10, 11].

The crucial role of carboxylic acids in atmospheric processes arises from their ability to act simultaneously as hydrogen‐bond donors and acceptors, enabling the formation of relatively strong hydrogen bonds (HBs) with other molecules. This property is pivotal in the stabilization of prenucleation clusters. In atmospheric cluster formation, stability is often discussed in terms of acid–base strength [12, 13, 14]. However, theoretical studies have shown that hydrogen‐bond topology can outweigh acid–base strength in determining cluster stability, highlighting the critical role of hydrogen‐bond networks [9, 15, 16, 17, 18]. For instance, formic acid is as effective as ammonia in forming clusters with sulfuric acid and water [17]. These findings underscore the need to investigate not only the hydrogen‐bonding interactions but also the broader spectrum of weak intermolecular forces that govern the clustering of carboxylic acids, in order to better understand their role in atmospheric nucleation.

At the molecular level, it is well established that the carboxylic acid dimer preferentially adopts a cyclic double‐hydrogen‐bonded structure with *C*_2h_ symmetry, which has long served as a prototype for studying double proton‐transfer dynamics in both theoretical and spectroscopic research [19, 20, 21]. For the formic acid trimer, a planar arrangement has been identified in which a monomer is linked to the cyclic dimer through one strong O─H···O═C hydrogen bond and an additional weak C─H···O═C hydrogen bond [22, 23]. A number of theoretical studies were dedicated to characterize the structures of the carboxylic acid tetramer, but the conclusion on the most stable structure remains controversial. Stein and Sauer first investigated formic acid tetramer using HF, MP2, and DFT calculations [24]. They identified three stable structures: a cyclic homodromic structure, a π–π stacking structure, and a “butterfly” structure. The butterfly structure was indicated to be the lowest in energy. Moreover, Ramon and Rios suggested a new chain‐like structure, in which two cyclic formic acid dimers are held together by two C─H···O═C weak HBs (wHBs), to be the most stable tetramer in condensed phases [25]. By evaluating 75 local minima on the tetramer potential surface, Roy and Thakkar also suggested this chain‐like structure to be the global minimum [26]. However, Wang pointed out that at the ab initio MP2 correlated level, the π–π stacking structure is more stable than the chain‐like structure [27]. Truhlar and Zhao further confirmed this result by using MP2/6‐31+G(*d*,*p*) calculations for geometry optimization and single‐point MP2 energies with augmented correlation‐consistent (cc) basis sets (aug‐cc‐pVDZ and aug‐cc‐pVTZ) [28]. Despite these calculations dating back nearly two decades, the theoretical establishment of the preference of the structures of carboxylic acid tetramers remains challenging. High‐resolution spectroscopic studies can provide a definitive benchmark for the debate on the subtle conformational arrangements.

Microwave spectroscopy has proven to be a powerful technique for determining molecular cluster structures, owing to its exceptional sensitivity to changes in rotational constants resulting from conformational variations and isotopic substitution [29, 30, 31, 32]. Following the pioneering work by Daly and co‐workers on the formic acid‐propiolic acid dimer in 2010, the double proton transfer in carboxylic acid dimers has been the subject of extensive experimental investigation [33, 34, 35, 36, 37, 38, 39, 40]. Nevertheless, rotational spectroscopic studies of larger carboxylic acid clusters remain scarce, with only a few systems characterized to date [23].

This study employs microwave spectroscopy to probe the molecular interactions and structural evolution of mixed formic acid (FA) and propiolic acid (PA) clusters. Based on isotopic labeling measurements and theoretical calculations, we have identified three trimers and two tetramers in the broadband spectra. The results reveal a clear structural transformation: trimers adopt planar structures dominated by hydrogen bonding, while tetramers are characterized by a π–π stacking arrangement. This transformation demonstrates that π–π stacking plays a decisive role in the stabilization and growth of larger clusters, introducing a new dimension of interaction beyond hydrogen bonding and creating additional docking sites for new particle formation.

## 2 Results and Discussion

### 2.1 Rotational Spectra Assignment

**FIGURE 1:**
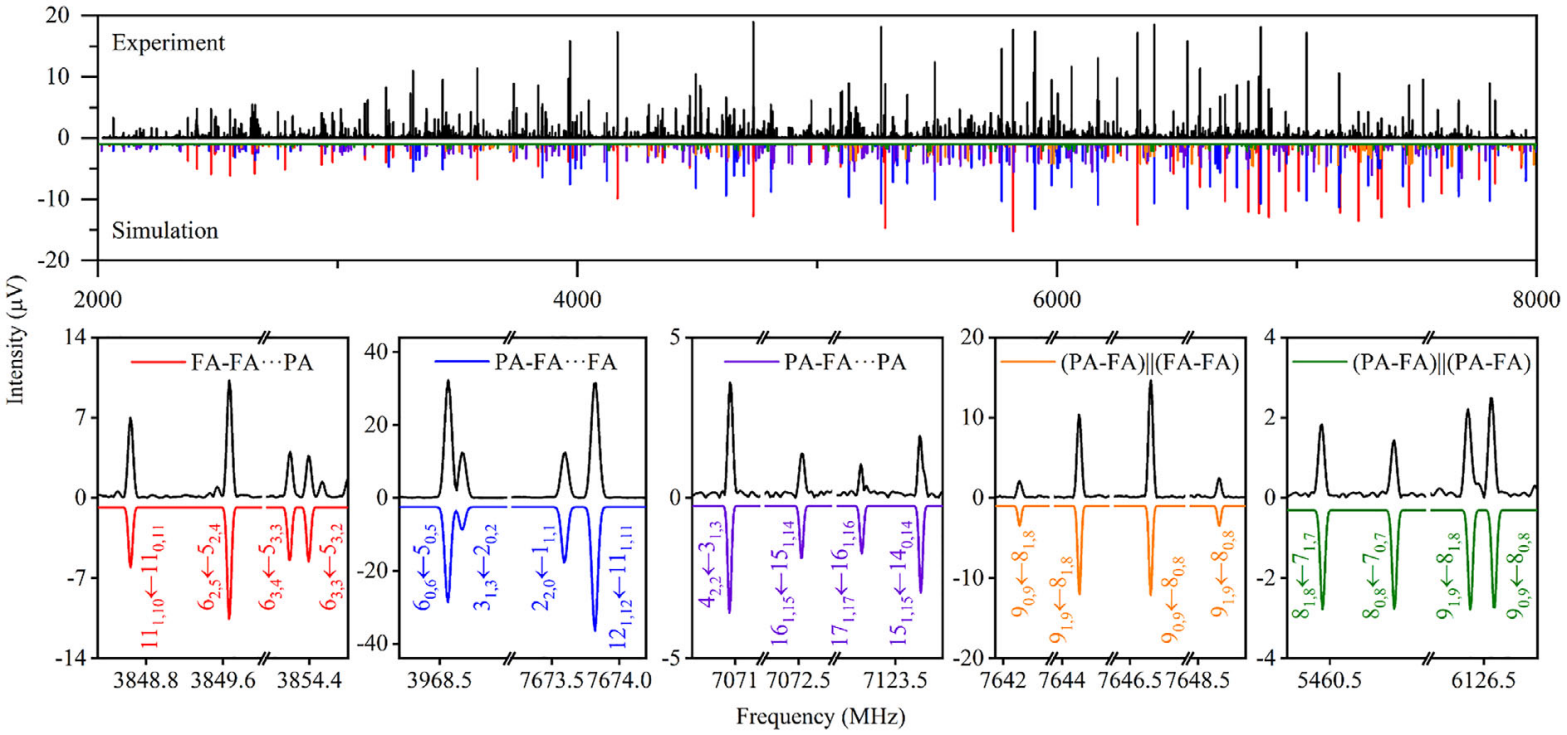
Broadband rotational spectra of the FA and PA mixture. The upper black trace corresponds to the experimental spectrum after removing the known spectral lines (see text). The lower traces in different colors represent simulated spectra from the experimental spectroscopic parameters (Table 1) at a rotational temperature of 1 K considering the theoretical dipole moment components for the respective complexes. At the bottom, parts of the spectra highlight representative transitions from each assigned cluster. The quantum numbers are defined using the standard nomenclature for the rotational energy levels of an asymmetric top, denoted as *J_Ka,Kc_*, where *J* is the quantum number for the total rotational angular momentum, and *K_a_* and *K_c_* are the quantum numbers for the projection of the total rotational angular momentum onto the rotational axes (*a* and *c*) in the two limiting cases of prolate and oblate symmetric tops, respectively.

The molecular clusters were generated by supersonic expansion as described in the Methods section of the Supporting Information. The spectrum covering the 2–8 GHz frequency range, as shown in Figure 1, was recorded using a chirped pulse Fourier transform microwave (CP‐FTMW) spectrometer by accumulating 11.4 million free induction decay (FID) signals. We first removed the spectral lines of previously reported species, including FA, PA, FA‐PA, and FA trimer [23, 33, 41, 42]. Based on the comprehensive configuration optimization and search for the potential clusters PA_m_FA_n_, the remaining strong spectral lines were assigned to two low‐lying conformers of the trimer PAFA_2_. Subsequently, we assigned another trimer PA_2_FA. Both *a*‐type and *b*‐type transitions were observed for the trimers. After removing these lines from the spectrum, two sets of weaker lines emerged, which were assigned to the tetramers PAFA_3_ and PA_2_FA_2_. Both *a*‐type and *b*‐type transitions of PAFA_3_ are observable, with the *a*‐type transitions slightly stronger than the *b*‐type transitions. For PA_2_FA_2_, the signals are weaker than those of PAFA_3_, with 68 *a*‐type transitions observed. These findings are consistent with the calculated dipole moment components (Table 1). These spectra were fitted to Watson's *S*‐reduced Hamiltonian in the *I^r^* representation using the AABS package and SPFIT from the CALPGM suite [43, 44].

**TABLE 1:** Comparison between the experimental and calculated spectroscopic parameters of the assigned PA_m_FA_n_ clusters.

Experiment					
Class	PAFA_2_		PA_2_FA	PAFA_3_	PA_2_FA_2_
Species	FA‐FA···PA	PA‐FA···FA	PA‐FA···PA	(PA‐FA)||(FA‐FA)	(PA‐FA)||(PA‐FA)
*A_0_* [MHz]	2675.95611(42)^a^	2439.23083(71)	1971.2863(21)	751.92570(61)	564.5414(47)
*B_0_* [MHz]	340.027027(81)	355.26294(12)	228.369568(76)	488.23405(19)	410.43259(35)
*C_0_* [MHz]	301.931574(79)	310.32666(12)	204.785132(70)	416.08219(17)	332.90770(36)
*D_J_* [kHz]	0.01795(30)	0.02238(49)	0.00901(13)	0.1610(13)	0.0147(19)
*D_JK_* [kHz]	−0.1266(30)	−0.3343(34)	−0.2438(31)	−0.1277(61)	0.575(19)
*D_K_* [kHz]	5.553(33)	5.870(79)	7.20(42)	0.530(41)	−1.02(17)
*d*_1_ [kHz]	−0.002930(46)	−0.00447(19)	−0.001500(73)	−0.04358(85)	−
*d*_2_ [kHz]	−0.000262(21)	−	−	−	−
μ_*a*_/μ_*b*_/μ_*c*_	Y/Y/N^b^	Y/Y/N	Y/Y/N	Y/Y/N	Y/N/N
σ [kHz]^c^	5.3	6.5	6.3	4.5	8.1
*N* ^d^	186	165	168	105	68
*P_cc_* [*u*Å^2^]^e^	0.66513(28)	0.59912(40)	0.75381(58)	246.30791(42)	304.2299(39)
*Δ_Exp_* [*u*Å^2^]^f^	−1.33026(56)	−1.19824(79)	−1.5076(12)	−492.61582(84)	−608.4598(77)

Calculation^g^					
*A_0_* [MHz]	2675.7	2448.7	1980.0	766.6	566.9
*B_0_* [MHz]	337.1	352.1	226.3	478.3	408.6
*C_0_* [MHz]	299.6	308.1	203.1	411.1	332.8
μ_*a*_/μ_*b*_/μ_*c*_ ^h^	0.8/1.3/0.0	2.2/1.1/0.0	1.7/1.5/0.0	0.6/0.3/0.1	1.3/0.0/0.0

^a^ Standard errors within parentheses are expressed in units of the last two digits.

^b^ Y (yes) and N (no) indicate whether the corresponding transition types were observed.

^c^ Root‐mean‐square deviation of the fit.

^d^ Number of the lines in the fit.

^e^ *P_cc_* is derived from the experimental moments of inertia by the equation: $P_{cc}=\sum_{i}m_{i}c_{i}^{2}=\frac{I_{a}+I_{b}-I_{c}}{2}\;$.

^f^ *Δ* indicates the inertial defect, which is calculated by the formula $\Delta =-2\sum_{i}m_{i}c_{i}^{2}=-2P_{cc}$, where *m_i_* and *c_i_* are the mass and the coordinate along the *c* axis of the *i*th particle, respectively.

^g^ The theoretical parameters were calculated at the revDSD‐PBEP86‐D3(BJ)/def2‐TZVPP level, and the rotational constants include vibrational corrections obtained at the B3LYP‐D3(BJ)/def2‐TZVP level.

^h^ Values of the dipole moment components are given in Debye.

The assigned species are named based on their structural characteristics. For trimers, the isomers are designated as FA‐FA···PA, PA‐FA···FA and PA‐FA···PA, respectively, where the hyphen ‘‐’ indicates that two moieties are connected by double hydrogen bonds between carboxyl groups, while ‘···’ represents hydrogen bonds formed between ─COOH and ─CHO groups. For tetramers, the assigned species are (PA‐FA)||(FA‐FA) and (PA‐FA)||(PA‐FA), where the symbol ‘||’ indicates π–π stacking between cyclic carboxylic acid dimers. Geometries and spectroscopic parameters were initially calculated at the B3LYP‐D4/def2‐TZVP level [45, 46, 47, 48] and subsequently reoptimized using different methods (see Tables S1–S4). The theoretical results in Table 1 were obtained at the revDSD‐PBEP86‐D3(BJ)/def2‐TZVPP level [47, 49, 50, 51] with vibrational corrections from B3LYP‐D3(BJ)/def2‐TZVP and agree with the experimental values within 2.0%.

The measured spectrum exhibits a sufficiently high signal‐to‐noise ratio for the trimers, with the strongest rotational transitions of FA‐FA···PA and PA‐FA···FA showing a signal‐to‐noise ratio of approximately 370:1, enabling the assignment of the singly ^13^C‐substituted species at natural abundance. To obtain more detailed structure information, we also measured the spectrum of deuterated species, employing a sample mixture of DCOOD, HCOOH, and HC≡CCOOH. Consequently, we successfully assigned five mono‐deuterated isotopologues and seven double‐deuterated isotopologues for FA‐FA···PA. For PA‐FA···FA, we identified five mono‐deuterated isotopologues and three dual‐deuterated isotopologues. For PA‐FA···PA, four mono‐deuterated isotopologues were identified. The experimental parameters of these isotopologues are provided in the Supporting Information. Our attempts to detect other isotopologues were unsuccessful, due to their overall low signal intensity and thus an insufficient number of transitions for accurate fitting.

### 2.2 Structural Analysis

**FIGURE 2:**
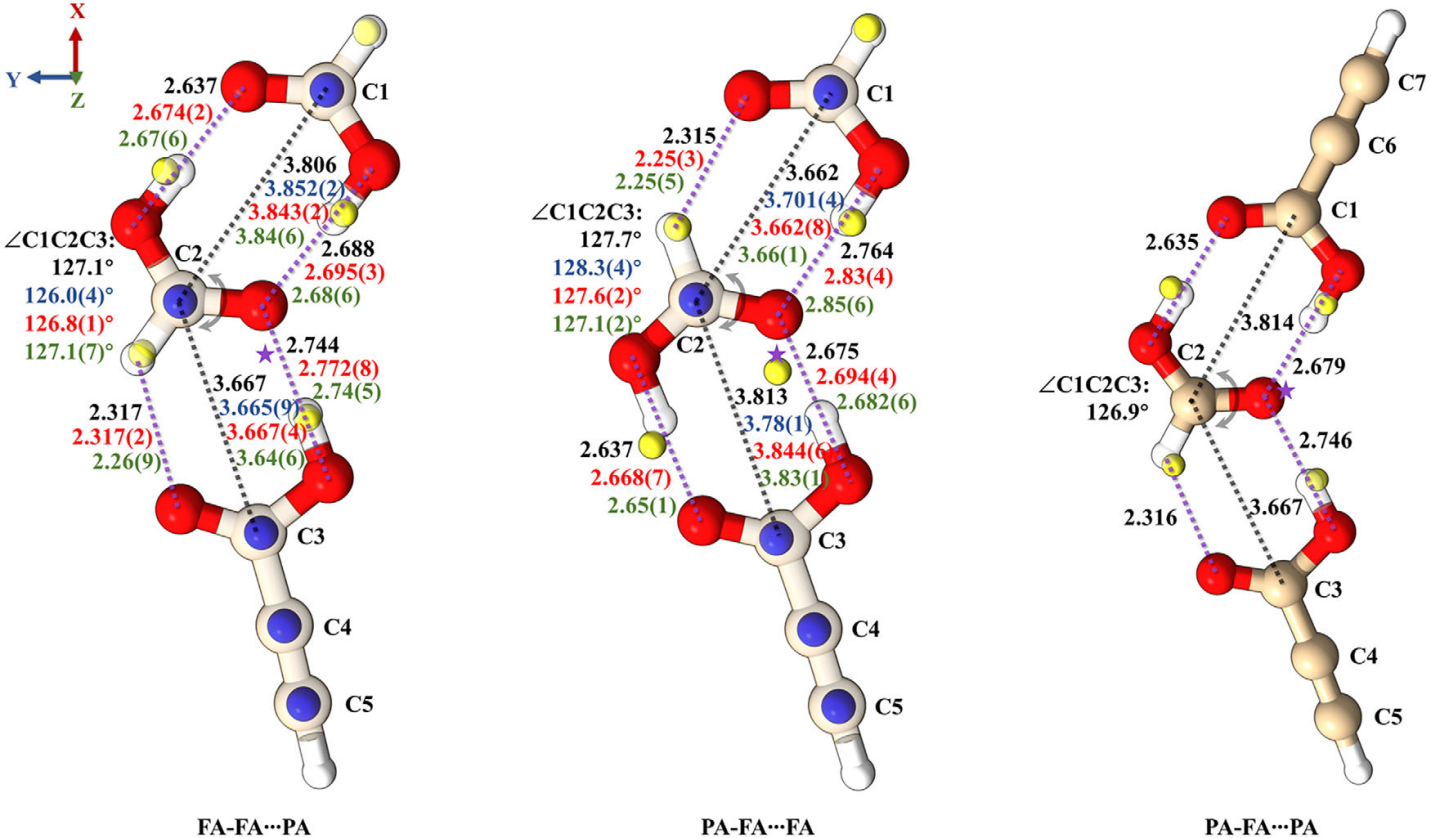
Observed structures for PAFA_2_ and PA_2_FA. The blue and yellow spheres represent the actual positions of the C and H atoms, derived from isotopic experiments and the Kraitchman's equations. The purple stars denote the centers of mass of the clusters. Black values indicate the *r_e_* structural parameters calculated at the B3LYP‐D4/def2‐TZVP level, whereas red, blue, and green values represent the *r_0_*, *r_s_* and *r_e_* ^SE^ structural parameters, respectively.

The substitution (*r_s_*) structure, effective ground‐state (*r_0_*) structure, and semi‐experimental equilibrium structure (*r_e_* ^SE^) can be derived from the experimental rotational constants of the parent and isotopically substituted species for a comprehensive structural analysis. These structures, compared with the theoretically optimized ones (*r_e_*), are depicted in Figure 2 (see details in the Supporting Information). The *r_s_* structure is determined using Kraitchman's equations, which allow us to extract the magnitudes of the coordinates of substituted atoms in the principal axis system of the moments of inertia without relying on structural assumptions from theoretical calculations [52]. This method provides valuable structural information but has inherent limitations. Specifically, determining the correct signs of the coordinates requires additional judgment based on computational results, and the uncertainty in Kraitchman's coordinates is inversely proportional to the distance of the substituted atom from the principal axes of inertia [53, 54]. As illustrated in Figure 2, the positions of the isotopically substituted atoms (blue and yellow spheres) closely match the theoretical calculations, further confirming our assignments. An exception is the hydrogen atom of the hydroxyl group in PA within the PA‐FA···FA trimer, which is located very close to the cluster's center of mass along the principal axes. This proximity, together with zero‐point vibrational effects of deuterium discussed later, leads to increased uncertainty in its determined position, resulting in a larger coordinate error and a noticeable deviation between the experimental *r*_s_ structure and the computed geometry for this hydrogen.

As an alternative, the *r_0_* structure was determined by fitting the moments of inertia of initial structures to the experimental moments of inertia of different isotopologues via a least‐squares method using the STRFIT program [53, 55]. This approach yields an effective geometry that incorporates vibrational effects in the ground vibrational state. The analysis utilized moments of inertia from the parent species and all singly ^13^C‐substituted isotopologues. Deuterated species were usually excluded because replacing hydrogen with deuterium violates the core assumption of *r_0_* calculations, namely that isotopic substitutions preserve the vibrational level of the ground state. Deuterium, however, has a significantly lower zero‐point vibrational energy than hydrogen. The intermolecular O···O distances and the angle formed by the three carboxyl carbon atoms were selected as floating parameters, while the remaining structural parameters were fixed to their theoretical values calculated at the B3LYP‐D4/def2‐TZVP level.

The $r_{e}^{\mathrm{SE}}$ approach, originally proposed by Pulay and co‐workers [56], combines experimental ground‐state rotational constants with theoretically calculated vibrational corrections to derive equilibrium geometries that are less affected by vibrational averaging. Recent benchmark studies indicate that vibrational corrections computed at the B3LYP level with an appropriate basis set are sufficiently accurate to obtain reliable structural information, even for relatively large molecular systems [45, 57, 58, 59]. Accordingly, we determined the $r_{e}^{\mathrm{SE}}$ structures using reference geometries optimized at the B2PLYP‐D3(BJ)/jun‐cc‐pVTZ level [49, 50, 60, 61] and vibrational corrections calculated at the B3LYP‐D3(BJ)/def2‐TZVP level [45, 46, 47, 49, 50]. Overall, the comparative analysis demonstrates good agreement between the *r_s_*, *r_0_*, and $r_{e}^{\mathrm{SE}}$ structures and the theoretical *r_e_* structures.

Within the cluster FA‐FA···PA, PA is attached to the cyclic FA dimer through one O−H···O HB as HB donor and one C−H···O wHB as acceptor, while in PA‐FA···FA, FA is attached to the FA end of the cyclic FA‐PA dimer. FA‐FA···PA is predicted to be 0.3 kJ mol^−1^ higher in energy than PA‐FA···FA after zero‐point energy (ZPE) and basis set superposition error (BSSE) corrections. The experimental relative abundance ratio of FA‐FA···PA to PA‐FA···FA is estimated to be approximately 3:2. The two isomers can interconvert via a 180° rotation of the central formic acid moiety around the C═O bond. The conversion barrier is calculated to be 55.8 kJ mol^−1^, as shown in Figure S2. For PA_2_FA, we observed the energetically preferred isomer PA‐FA···PA, in which the PA monomer is held to the FA end of the cyclic PA‐FA dimer. The rotation of the central FA monomer in this configuration follows a mechanism analogous to the interconversion described above. However, for PA‐FA···PA, this motion connects two equivalent conformers along a symmetric double‐well potential. It is interesting to note that, given this identical motion mode, the barrier is calculated to be the same as above, 55.8 kJ mol^−1^ (Figure S4). Such a high barrier effectively precludes interconversion via quantum tunneling. The second most stable isomer of PA_2_FA is predicted to be 3.8 kJ mol^−1^ higher in energy than PA‐FA···PA and adopts a nonplanar structure, where three carboxylic acids form a twisted cyclic arrangement through three HBs (Figure S3).

The planarity of the assigned structures of these trimers can be evidenced by their planar moments of inertia *P_cc_*, which reflect the mass distribution along the *c‐*axis. The *P_cc_* values derived from fitted rotational constants for FA‐FA···PA, PA‐FA···FA, and PA‐FA···PA are 0.66512(30) *u*Å^2^, 0.59912(40) *u*Å^2^, and 0.75974(87) *u*Å^2^, respectively. These values are very small, indicating their planar skeletal structure. The positive values of *P_cc_* can be attributed to out‐of‐plane vibrations. The lowest frequency of out‐of‐plane vibrations (*v*_1_) can be estimated using Oka's equation: $\Delta_{0}=\Delta_{01}+\alpha \;\sqrt{I_{cc}}=-\frac{33.715}{v_{1}}+\alpha \sqrt{I_{cc}}$, with the empirical constant α  =  0.00803, suitable for aromatic molecules [62]. The frequencies determined from the equation are 20.33, 22.15, and 17.57 cm^−1^, respectively, which are in excellent agreement with the lowest out‐of‐plane vibrational frequencies (23.43, 25.59, and 20.14 cm^−1^) predicted by theoretical calculations for the planar structures.

The hydrogen bonds, the purple dashed lines in Figure 2, can be classified into two types: HB and wHB. The HBs form between the hydrogen atom of the hydroxyl group (O─H) and the oxygen atom of the carbonyl group (O─H···O═C), with O···O theoretical distances ranging from 2.64 to 2.76 Å. The oxygen atom of the C═O group of the central FA of the trimers acts as HB acceptor, forming two HBs with neighboring carboxylic acids. Theoretical calculations show that the HB distance in the cyclic dimers (∼2.68 Å) is shorter than that between the central molecule and the third carboxylic acid (∼2.75 Å). This finding is further supported by the *r_0_*, *r_s_*, and *r*_e_^SE^ structures, despite minor deviations among them. The wHB forms between the hydrogen atom in the C─H bond and the oxygen atom of the carbonyl group, with a distance of approximately 2.3 Å (H···O). In the cyclic dimers connected by two O─H⋯O═C hydrogen bonds, the distance between the carbon atoms has been determined to be 3.78–3.85 Å. Meanwhile, the distance between the carboxyl carbon atoms of the third carboxylic acid and the central FA ranges from 3.64 to 3.70 Å. The ∠C1C2C3 angles formed by the carboxyl carbons of the carboxyl groups were determined to be between 126.0° and 128.3°.

**FIGURE 3:**
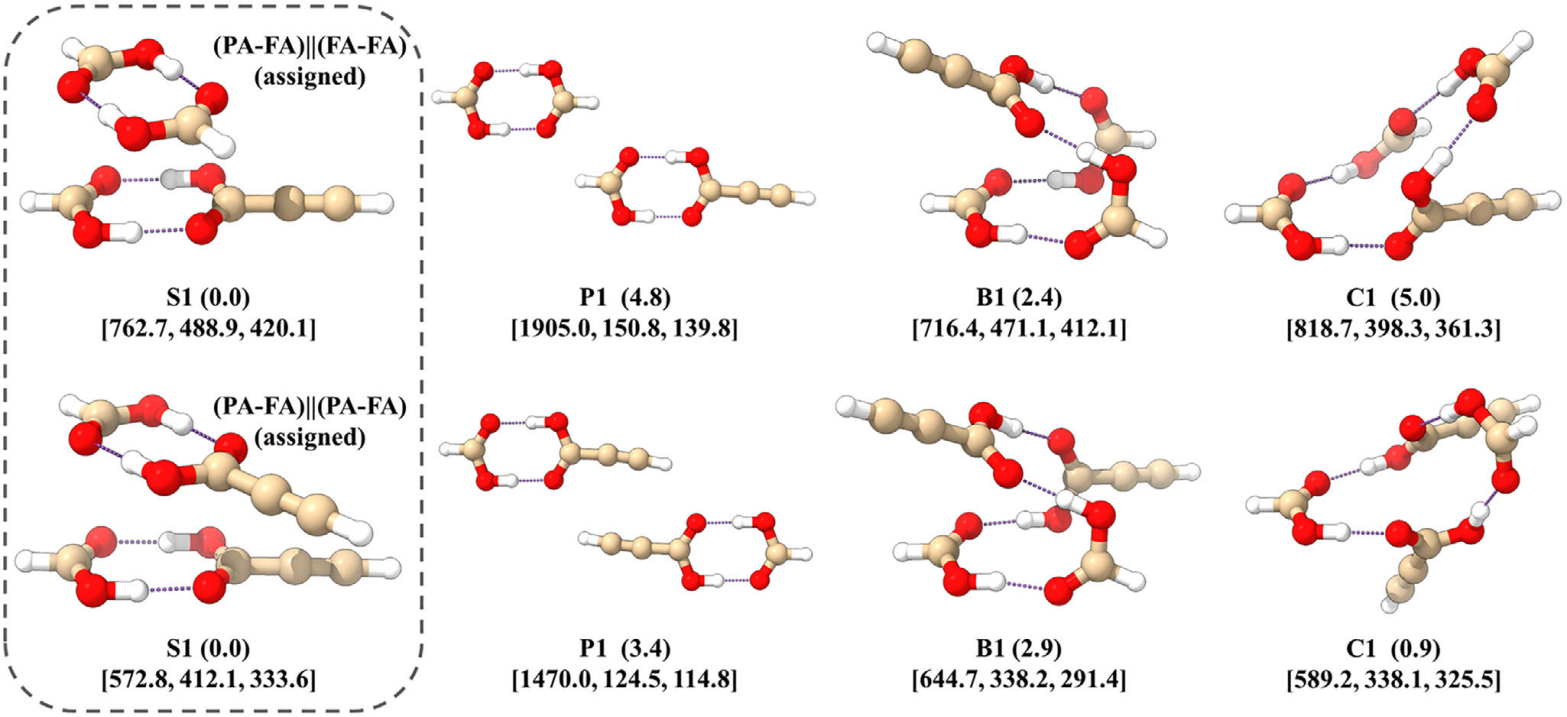
Low‐energy isomers of PAFA_3_ and PA_2_FA_2_: stacking (S), planar (P), butterfly (B), and cyclic (C) structures. These structures are named according to their classes and followed by an Arabic numeral, which corresponds to their energy ordering within the respective classes, with 1 being the most stable structure at the B3LYP‐D4/def2‐TZVP level of theory. The energy ordering includes corrections for ZPE and BSSE. Values in parentheses denote relative energies (in kJ mol^−1^), and values in square brackets indicate rotational constants (in MHz).

For tetramers, four distinct classes of isomers can be identified: stacking (S), planar (P), butterfly (B), and cyclic (C) structures. Figure 3 displays only the most stable representative from each category, with additional structures depicted in Figures S5 and S6. Stacked structures, composed of two cyclic carboxylic acid dimers, are identified as the global minima for both PAFA_3_ and PA_2_FA_2_, consistent with the findings of Wang, Truhlar, and Zhao [27, 28]. In these global minimum configurations, the two carboxylic acid dimers are nearly parallel but staggered when viewed from above. Depending on the H‐atom positions within the cyclic hydrogen bonds, several quasi‐degenerate configurations exist (see Figures S5 and S6), which may interconvert (or relax) via proton transfer. When the two dimers lie in the same plane, they interact either through two wHBs involving the CHO groups of FA in PAFA_3_ or via interactions between acetylene groups in PA_2_FA_2_. This planar configuration has been described as a chain‐like structure in previous studies [25, 26]. Furthermore, butterfly and cyclic structures feature four carboxylic acid groups connected end‐to‐end through four HBs, leading to diverse configurations due to variations in hydrogen bonding sites. Notably, in the cyclic PAFA_3_ structure, PA adopts the higher‐energy *cis* conformation, which is energetically unfavorable in its monomeric form. Monomeric *cis*‐PA lies 12.6 kJ mol^−1^ above *trans*‐PA in energy, while *cis*‐PAFA_3_ is 0.7 kJ mol^−1^ more stable than its *trans* counterpart, at the B3LYP‐D4/def2‐TZVP level of theory. The absence of other predicted isomers in our spectra is likely due to their higher energies relative to the corresponding assigned structures and, for some isomers, dipole moments that are insufficiently large to be detected.

**FIGURE 4:**
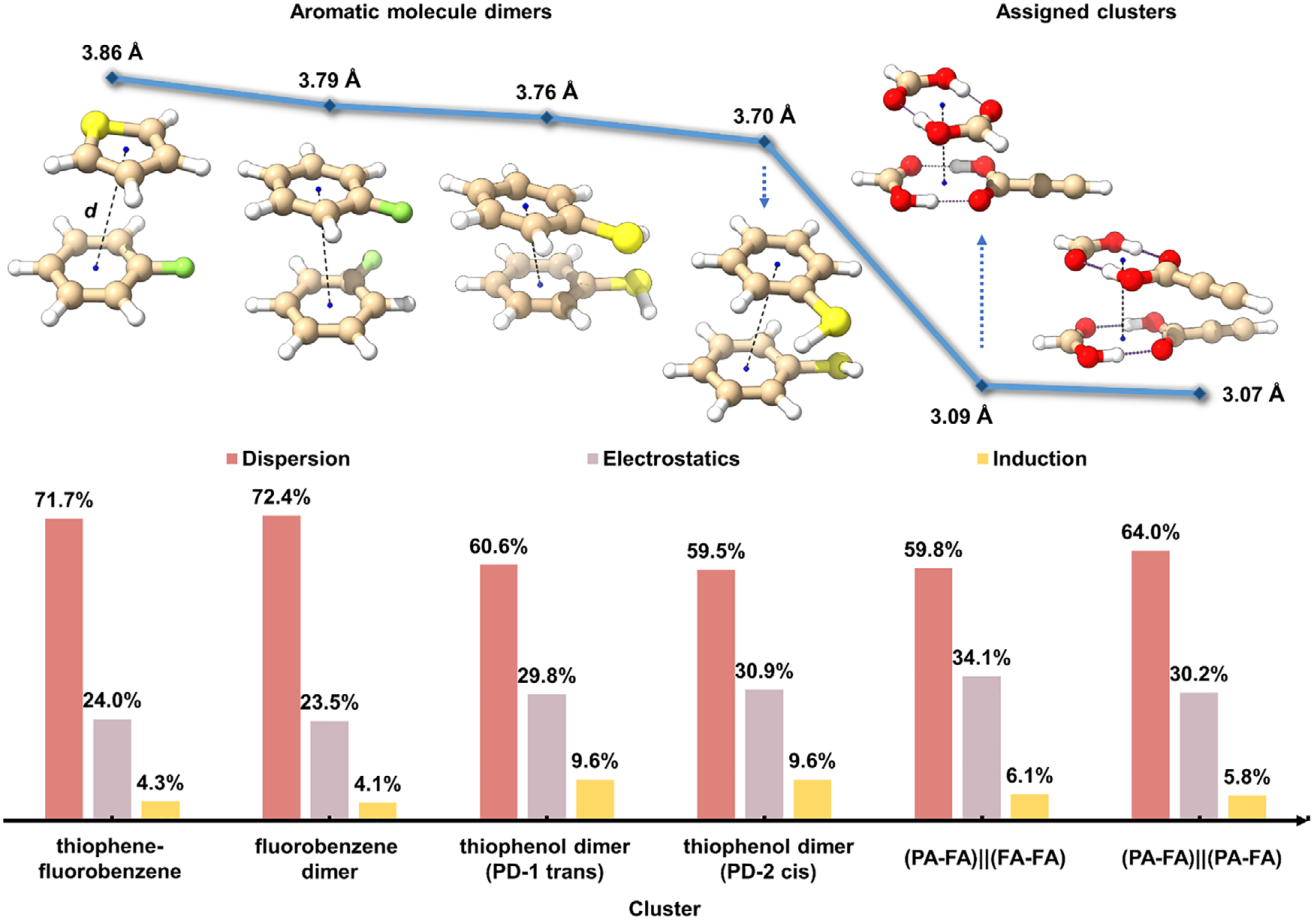
Layer distances and SAPT energy decomposition of selected aromatic molecule dimers compared with the experimentally observed carboxylic acid clusters. The upper line chart shows the theoretical distances (*d*) between the geometric centers of the interacting layers, while the lower bar charts display the contributions of electrostatic, induction, and dispersion energies as percentages of the total attractive interactions in each complex, calculated at the SAPT2+(3)δMP2/aug‐cc‐pVTZ level.

Through a systematic evaluation of all possible isomer classes, combining an in‐house developed automated spectral fitting program with manual analysis, we conclusively identified the global minima S1 for both tetramers present in our spectrum (Figure 3): (PA‐FA)||(FA‐FA) and (PA‐FA)||(PA‐FA). The structures of the assigned clusters exhibit characteristics similar to π–π stacking, which is also a conventional arrangement in aromatic ring dimers. Aromatic ring homodimers and heterodimers, including the thiophenol dimer, fluorobenzene dimer, and thiophene‐fluorobenzene dimer, present similar parallel and staggered structures, which have been confirmed by microwave spectroscopy [63, 64]. We reproduced these structures using the B3LYP‐D4/def2‐TZVP level of theory and compared them with the assigned (PA‐FA)||(FA‐FA) and (PA‐FA)||(PA‐FA) structures. All of them can be considered as two‐layered, and the distances (*d*) here refer to the separation between the geometric centers of the “rings” in each layer. In aromatic ring dimers, the rings are the carbon skeletons of benzene fragments, while in the assigned clusters they are eight‐membered rings formed by the carboxyl group of each carboxylic acid in the layer. As shown in Figure 4, the calculated interlayer distances (*d*) for the thiophene–fluorobenzene dimer, fluorobenzene dimer, thiophenol dimer isomer 1 (PD‐1 trans), thiophenol dimer isomer 2 (PD‐2 cis), (PA‐FA)||(FA‐FA), and (PA‐FA)||(PA‐FA) are 3.86, 3.79, 3.76, 3.70, 3.09, and 3.07 Å, respectively. The pronounced decrease in the values observed for the last two clusters indicates that the (PA‐FA)||(FA‐FA) and (PA‐FA)||(PA‐FA) assemblies adopt significantly more compact configurations relative to these aromatic ring dimers.

### 2.3 Noncovalent Interaction Analysis

To better understand the origin of this structural compactness and the molecular interactions within the assigned clusters, we performed two‐body symmetry‐adapted perturbation theory (SAPT) energy decomposition calculations using Psi4 at the SAPT2+(3)δ(MP2)/aug‐cc‐pVTZ level of theory [65, 66]. SAPT calculations decompose the binding energy between two fragments into four components: electrostatics, exchange, dispersion, and induction. In the carboxylic acid tetramer, we considered each cyclic carboxylic acid dimer as one unit to examine the intermolecular forces between the carboxylic acid dimer rings. As comparison, we also performed SAPT analyses for the aromatic dimers discussed above (Figure 4). The dominant interactions between the carboxylic acid dimer rings are dispersion forces, which are also the leading forces in π–π stacking structures. (PA‐FA)||(PA‐FA) exhibits a greater dispersion contribution compared to (PA‐FA)||(FA‐FA), which can be attributed to interactions between the C≡C groups of PA. The carboxylic acid tetramers exhibit over 30% electrostatic interaction between the two layers, a contribution comparable to that of the thiophenol dimer but greater than those of the fluorobenzene dimer and the thiophene‐fluorobenzene dimer. This enhanced interaction arises from the electrostatic attraction between the electropositive hydrogen atoms of OH/SH groups and electronegative oxygen/sulfur atoms in adjacent layers. Although aromatic ring dimers show greater stabilization energies, the carboxylic acid tetramers adopt a more compact structure. This compactness arises from reduced π‐orbital overlap and thus weaker repulsion between the C═O bonds, compared with the more extensive and diffuse π‐overlap between aromatic rings (Figures S7 and S8).

**FIGURE 5:**
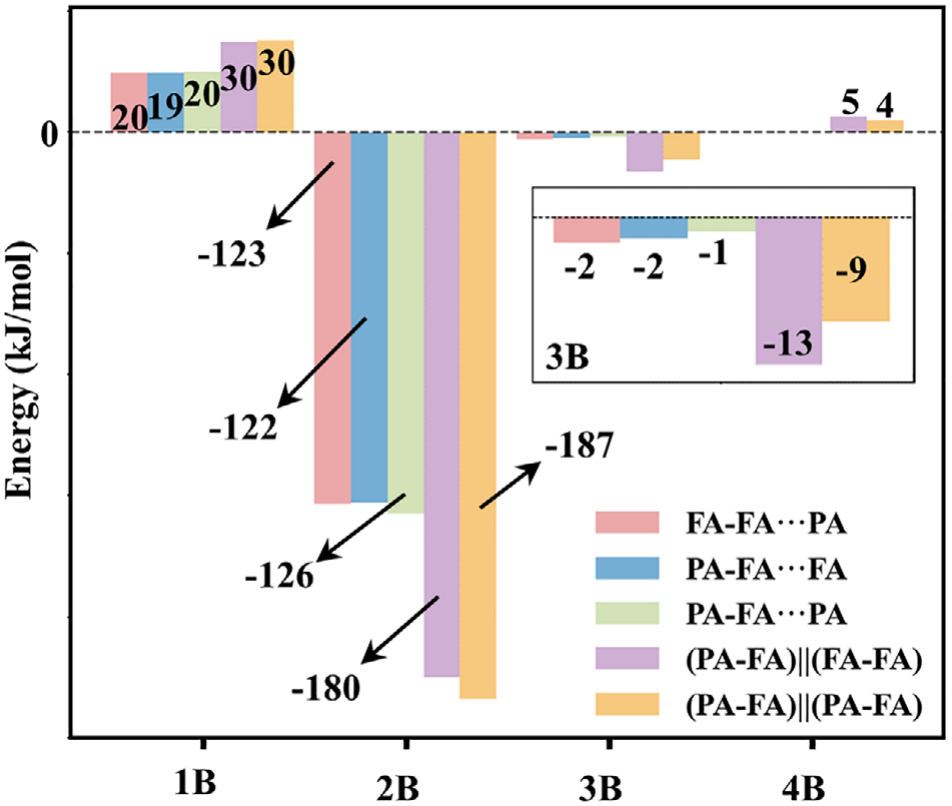
MBE analysis including ZPE corrections for the assigned carboxylic acid clusters.

In addition, we applied many‐body expansion (MBE) analysis to explore the trends in specific interactions between molecular pairs, triples, and higher‐order groups as the system evolves from trimers to tetramers (Figure 5) [67, 68, 69]. The influence of π–π stacking is particularly evident in the three‐body and four‐body terms. In the three‐body interactions, the pronounced increase observed from trimers to tetramers reflects the participation of π–π stacking, which enhances cooperative effects in the two‐layered structures. In pure water clusters or other hydrogen‐bonded systems, the four‐body term is usually negative due to hydrogen bond cooperativity [68, 69, 70]. Notably, in our tetramers, the four‐body term becomes positive (3.95–5.05 kJ mol^−1^), indicating an anticooperative effect likely caused by steric strain or suboptimal π–π stacking alignment. Overall, the increased two‐ and three‐body interactions in the tetramers outweigh the unfavorable four‐body contributions, thereby stabilizing the stacked‐layer structures.

## 3 Conclusion

In summary, using rotational spectroscopy, we have characterized the structures of trimeric and tetrameric clusters formed by formic acid and propiolic acid. Our results demonstrate that carboxylic acid trimers adopt planar geometries stabilized by cyclic hydrogen bonding networks, while tetramers form stacked structures mediated by π–π interactions. Notably, the interlayer distances in these tetramers are significantly shorter than those in typical aromatic dimers, suggesting reduced π‐cloud repulsion. Despite their distinct architectures, our SAPT analysis shows that dispersion forces dominate interlayer interactions in both systems, contributing comparably to the overall binding stability. Many‐body expansion analysis reveals a delicate balance between cooperative and anticooperative effects with increasing cluster size.

The observed planar‐to‐stacked transition provides a framework for understanding molecular assembly in carboxylic acid systems, particularly the critical size at which π‐stacking begins to compete with and eventually surpass hydrogen bonding as the dominant organizational principle. This finding can provide mechanistic understanding for cluster‐mediated atmospheric processes, particularly new particle formation and aerosol growth. The enhanced molecular docking capacity of stacked tetramers suggests that these structures may serve as effective nucleation precursors, a hypothesis that could be tested through coupled experimental‐computational investigations of larger clusters. Finally, the methodological approach demonstrated here, combining rotational spectroscopy with advanced energy decomposition analysis, establishes a powerful paradigm for probing subtle intermolecular forces in increasingly complex molecular assemblies, with potential applications spanning atmospheric chemistry, materials science, and drug design.

## Conflicts of Interest

The authors declare no conflicts of interest.

## Supporting information



The experimental and computational data supporting this article have been included in the Supporting Information, which is provided as a PDF file. The PDF file contains supporting text, Figures S1–S8, Tables S1–S67 and references.
**Supporting File 1**: anie71536‐sup‐0001‐SuppMat.pdf.

## Data Availability

The data that support the findings of this study are available in the Supporting Information of this article.
