# Supplementary material for: Beyond Hydrogen Bonding: π···π Stacking Directed Self‐Assembly of Carboxylic Acid Clusters in the Gas Phase
Source: Angew Chem Int Ed Engl. 2026 Feb 17;65(13):e23854. doi: 10.1002/anie.202523854 (PMC13007584; doi:10.1002/anie.202523854)
Supplement: Supplementary file 1 — The experimental and computational data supporting this article have been included in the Supporting Information, which is provided as a PDF file. The PDF file contains supporting text, Figures S1–S8, Tables S1–S67 and references. Supporting File 1: anie71536‐sup‐0001‐SuppMat.pdf. [file ANIE-65-e23854-s001.pdf]

Supporting Information for

Beyond Hydrogen Bonding: π···π Stacking Directed Self-Assembly of Carboxylic Acid Clusters in the Gas Phase

Jingling Hong[1], Melanie Schnell[2,3,*], Mingfei Zhou[1], Weixing Li[1,*]

[1]*Department of Chemistry, State Key Laboratory of Porous Materials for Separation and Conversion, Shanghai Key Laboratory of Molecular Catalysis and Innovative Materials, Fudan University, Songhu Rd. 2005, 200438 Shanghai, China.*

[2]*Deutsches Elektronen-Synchrotron DESY, Notkestr. 85, 22607 Hamburg, Germany.*

[3]*Institut für Physikalische Chemie, Christian-Albrechts-Universität zu Kiel, Max-Eyth-Str. 1, 24118 Kiel, Germany.*

*Corresponding authors: melanie.schnell@desy.de; weixingli@fudan.edu.cn

Table of Contents

Methods 8

Experimental details 8

Spectral Fitting 8

Isomer abundances 8

Theoretical details 9

Structural Determination 10

Figures 11

Figure S1. Low-energy isomers of $PAFA_2$. Values in parentheses give relative energies (kJ/mol, B3LYP-D4/def2-TZVP with ZPE and BSSE corrections), brackets list the rotational constants (A, B, C in MHz), and curly braces denote the dipole moment components (μ_a, μ_b, μ_c in Debye). 11

Figure S2. The conversion barrier between FA-FA···PA and PA-FA···FA calculated at the B3LYP-D4/def2-TZVP level. 12

Figure S3. Low-energy isomers of PA_2FA. Values in parentheses give relative energies (kJ/mol, B3LYP-D4/def2-TZVP with ZPE and BSSE corrections), brackets list the rotational constants (A, B, C in MHz), and curly braces denote the dipole moment components (μ_a, μ_b, μ_c in Debye). 13

Figure S4. Rotation barrier of the FA unit in the PA-FA···PA cluster calculated at the B3LYP-D4/def2-TZVP level. 14

Figure S5. Low-energy isomers of $PAFA_3$: stacking (S), planar (P), butterfly (B), and cyclic (C) structures. These structures are named according to their classes, and followed by an Arabic numeral, which corresponds to their energy ordering within the respective classes, with 1 being the most stable structure. Values in parentheses give relative energies (kJ/mol, B3LYP-D4/def2-TZVP with ZPE and BSSE corrections), brackets list the rotational constants (A, B, C in MHz), and curly braces denote the dipole moment components (μ_a, μ_b, μ_c in Debye). 15

Figure S6. Low-energy isomers of PA_2FA_2: stacking (S), planar (P), butterfly (B), and cyclic (C) structures. These structures are named according to their classes, and followed by an Arabic numeral, which corresponds to their energy ordering within the respective classes, with 1 being the most stable structure. Values in parentheses give relative energies (kJ/mol, B3LYP-D4/def2-TZVP with ZPE and BSSE corrections), brackets list the rotational constants (A, B, C in MHz), and curly braces denote the dipole moment components (μ_a, μ_b, μ_c in Debye). 16

Figure S7. LOL-π isosurfaces of selected aromatic ring dimers (isovalue = 0.3). 17

Figure S8. Top and side views of the LOL-π isosurfaces for (PA-FA)||(FA-FA) and (PA-FA)||(PA-FA) (isovalue = 0.15). 17

Tables 18

Table S1. Calculated spectroscopic parameters for the assigned PA_mFA_n clusters at the revDSD-PBEP86-D3(BJ)/def2-TZVPP level. 18

Table S2. Calculated spectroscopic parameters for the assigned PA_mFA_n clusters at the B2PLYP-D3(BJ)/jun-cc-pVTZ level. 18

Table S3. Calculated spectroscopic parameters for the assigned PA_mFA_n clusters at the B3LYP-D4/def2-TZVP level. 19

Table S4. Calculated spectroscopic parameters for the assigned PA_mFA_n clusters at the MP2/aug-cc-pVTZ level. 19

Table S5. Vibrational corrections of the parent and five ^{13}C isotopologues for FA-FA···PA used in the semi-experimental equilibrium structure analysis. 20

Table S6. Vibrational corrections of the parent and five ^{13}C isotopologues for PA-FA···FA used in the semi-experimental equilibrium structure analysis. 20

Table S7. Boltzmann populations (%) for $PAFA_2$. 21

Table S8. Boltzmann populations (%) for PA_2FA. 21

Table S9. Boltzmann populations (%) for $PAFA_3$. 21

Table S10. Boltzmann populations (%) for PA_2FA_2. 21

Table S11. Results of many-body expansion analysis, in kJ/mol. 22

Table S12. Results of two-body SAPT energy decomposition calculations at the SAPT2+(3)δ(MP2)/aug-cc-pVTZ level, in kJ/mol. 22

Table S13. Experimental spectroscopic parameters for singly ^{13}C-substituted isotopologues of the FA-FA···PA cluster. 23

Table S14. Experimental spectroscopic parameters for singly deuterium-substituted isotopologues of the FA-FA···PA cluster. 24

Table S15. Experimental spectroscopic parameters for double deuterium-substituted isotopologues of the FA-FA···PA cluster. 25

Table S16. Experimental spectroscopic parameters for singly ^{13}C-substituted isotopologues of the PA-FA···FA cluster. 26

Table S17. Experimental spectroscopic parameters for singly deuterium-substituted isotopologues of the PA-FA···FA cluster. 27

Table S18. Experimental spectroscopic parameters for double deuterium-substituted isotopologues of the PA-FA···FA cluster. 28

Table S19. Experimental spectroscopic parameters for singly deuterium-substituted isotopologues of the PA-FA···PA cluster. 29

Table S20. Experimental transition frequencies (v/MHz) together with the corresponding observed - calculated differences (Δv/MHz) for the parent FA-FA···PA complex. 30

Table S21. Experimental transition frequencies (v/MHz) together with the corresponding observed - calculated differences (Δv/MHz) for the ^{13}C1 isotopologue of the FA-FA···PA complex. 35

Table S22. Experimental transition frequencies (v/MHz) together with the corresponding observed - calculated differences (Δv/MHz) for the ^{13}C2 isotopologue of the FA-FA···PA complex. 36

Table S23. Experimental transition frequencies (v/MHz) together with the corresponding observed - calculated differences (Δv/MHz) for the ^{13}C3 isotopologue of the FA-FA···PA complex. 37

Table S24. Experimental transition frequencies (v/MHz) together with the corresponding observed - calculated differences (Δv/MHz) for the ^{13}C4 isotopologue of the FA-FA···PA complex. 38

Table S25. Experimental transition frequencies (v/MHz) together with the corresponding observed - calculated differences (Δv/MHz) for the ^{13}C5 isotopologue of the FA-FA···PA complex. 39

Table S26. Experimental transition frequencies (v/MHz) together with the corresponding observed - calculated differences (Δv/MHz) for the D1 isotopologue of the FA-FA···PA complex. 40

Table S27. Experimental transition frequencies (v/MHz) together with the corresponding observed - calculated differences (Δv/MHz) for the D2 isotopologue of the FA-FA···PA complex. 42

Table S28. Experimental transition frequencies (v/MHz) together with the corresponding observed - calculated differences (Δv/MHz) for the D3 isotopologue of the FA-FA···PA complex. 44

Table S29. Experimental transition frequencies (v/MHz) together with the corresponding observed - calculated differences (Δv/MHz) for the D4 isotopologue of the FA-FA···PA complex. 47

Table S30. Experimental transition frequencies (v/MHz) together with the corresponding observed - calculated differences (Δv/MHz) for the D5 isotopologue of the FA-FA···PA complex. 49

Table S31. Experimental transition frequencies (ν/MHz) together with the corresponding observed - calculated differences (Δν/MHz) for the doubly deuterated (D1 and D2) isotopologue of the FA-FA···PA complex. 51

Table S32. Experimental transition frequencies (ν/MHz) together with the corresponding observed - calculated differences (Δν/MHz) for the doubly deuterated (D1 and D3) isotopologue of the FA-FA···PA complex. 53

Table S33. Experimental transition frequencies (ν/MHz) together with the corresponding observed - calculated differences (Δν/MHz) for the doubly deuterated (D1 and D4) isotopologue of the FA-FA···PA complex. 56

Table S34. Experimental transition frequencies (ν/MHz) together with the corresponding observed - calculated differences (Δν/MHz) for the doubly deuterated (D1 and D5) isotopologue of the FA-FA···PA complex. 58

Table S35. Experimental transition frequencies (ν/MHz) together with the corresponding observed - calculated differences (Δν/MHz) for the doubly deuterated (D2 and D3) isotopologue of the FA-FA···PA complex. 59

Table S36. Experimental transition frequencies (ν/MHz) together with the corresponding observed - calculated differences (Δν/MHz) for the doubly deuterated (D3 and D4) isotopologue of the FA-FA···PA complex. 61

Table S37. Experimental transition frequencies (ν/MHz) together with the corresponding observed - calculated differences (Δν/MHz) for the doubly deuterated (D3 and D5) isotopologue of the FA-FA···PA complex. 63

Table S39. Experimental transition frequencies (ν/MHz) together with the corresponding observed - calculated differences (Δν/MHz) for the ^{13}C1 isotopologue of the PA-FA···FA complex. 68

Table S40. Experimental transition frequencies (ν/MHz) together with the corresponding observed - calculated differences (Δν/MHz) for the ^{13}C2 isotopologue of the PA-FA···FA complex. 69

Table S41. Experimental transition frequencies (ν/MHz) together with the corresponding observed - calculated differences (Δν/MHz) for the ^{13}C3 isotopologue of the PA-FA···FA complex. 70

Table S42. Experimental transition frequencies (ν/MHz) together with the corresponding observed - calculated differences (Δν/MHz) for the ^{13}C4 isotopologue of the PA-FA···FA complex. 71

Table S43. Experimental transition frequencies (ν/MHz) together with the corresponding observed - calculated differences (Δν/MHz) for the ^{13}C5 isotopologue of the PA-FA···FA complex. 72

Table S44. Experimental transition frequencies (ν/MHz) together with the corresponding observed - calculated differences (Δν/MHz) for the D1 isotopologue of the PA-FA···FA complex. 73

Table S45. Experimental transition frequencies (ν/MHz) together with the corresponding observed - calculated differences (Δν/MHz) for the D2 isotopologue of the PA-FA···FA complex. 75

Table S46. Experimental transition frequencies (ν/MHz) together with the corresponding observed - calculated differences (Δν/MHz) for the D3 isotopologue of the PA-FA···FA complex. 77

Table S47. Experimental transition frequencies (ν/MHz) together with the corresponding observed - calculated differences (Δν/MHz) for the D4 isotopologue of the PA-FA···FA complex. 79

Table S48. Experimental transition frequencies (ν/MHz) together with the corresponding observed - calculated differences (Δν/MHz) for the D5 isotopologue of the PA-FA···FA complex. 81

Table S49. Experimental transition frequencies (ν/MHz) together with the corresponding observed - calculated differences (Δν/MHz) for the doubly deuterated (D1 and D2) isotopologue of the PA-FA···FA complex. 84

Table S50. Experimental transition frequencies (ν/MHz) together with the corresponding observed - calculated differences (Δν/MHz) for the doubly deuterated (D1 and D3) isotopologue of the PA-FA···FA complex. 85

Table S51. Experimental transition frequencies (ν/MHz) together with the corresponding observed - calculated differences (Δν/MHz) for the doubly deuterated (D1 and D4) isotopologue of the PA-FA···FA complex. 87

Table S52. Experimental transition frequencies (ν/MHz) together with the corresponding observed - calculated differences (Δν/MHz) for the parent PA-FA···PA complex. 88

Table S54. Experimental transition frequencies (ν/MHz) together with the corresponding observed - calculated differences (Δν/MHz) for the D2 isotopologue of the PA-FA···PA complex. 95

Table S55. Experimental transition frequencies (ν/MHz) together with the corresponding observed - calculated differences (Δν/MHz) for the D3 isotopologue of the PA-FA···PA complex. 99

Table S56. Experimental transition frequencies (ν/MHz) together with the corresponding observed - calculated differences (Δν/MHz) for the D4 isotopologue of the PA-FA···PA complex. 102

Table S57. Experimental transition frequencies (ν/MHz) together with the corresponding observed - calculated differences (Δν/MHz) for the parent (PA-FA)||(FA-FA) complex. 105

Table S58. Experimental transition frequencies (ν/MHz) together with the corresponding observed - calculated differences (Δν/MHz) for the parent (PA-FA)||(PA-FA) complex. 108

Table S59. Equilibrium (r_e) and substitution (r_s) coordinates of the FA-FA···PA complex. 110

Table S60. Effective ground-state (r_0) coordinates of the FA-FA···PA complex. 111

Table S61. Semi-experimental equilibrium (r_e^{SE}) coordinates of the FA-FA···PA complex. 112

Table S62. Equilibrium (r_e) and substitution (r_s) coordinates of the PA-FA···FA complex. 113

Table S63. Effective ground-state (r_0) coordinates of the PA-FA···FA complex. 114

Table S64. Semi-experimental equilibrium (r_e^{SE}) coordinates of the PA-FA···FA complex. 115

Table S65. Equilibrium (r_e) and substitution (r_s) coordinates of the PA-FA···PA complex. 116

Table S66. Equilibrium (r_e) coordinates of the (PA-FA)||(FA-FA) complex. 117

Table S67. Equilibrium (r_e) coordinates of the (PA-FA)||(PA-FA) complex. 118

References 119

Methods

Experimental details

The microwave spectra of FA_mPA_n clusters were recorded using two broadband CP-FTMW spectrometers. All samples are used without further purification, including formic acid (Aladdin, HCOOH, purity ≥ 98%), propiolic acid (Admas-beta, HCCCOOH, purity ≥ 98%) and formic acid-d_2 (Isotope, DCOOD, purity ≥ 98%). Propiolic acid was placed in a reservoir, which is custom-made as part of the pulsed valve (General Valve series 9), located close to the valve orifice, and heated to 65 °C. A gas mixture of ca. 0.5% FA in neon as carrier gas at a stagnation pressure of about 4 MPa was expanded into the vacuum chamber (~5×10^{-5} Pa) through a 1 mm diameter nozzle. We initially collected the spectrum in the range of 2-8 GHz at DESY.[1] Then, we replicated the experiment at Fudan University[2] and conducted the isotopic substitution experiments. For the measurement within the 2-8 GHz frequency range, after a delay of 960 μs from the trigger of the pulsed nozzle with an opening duration of 500 μs, six back-to-back chirped pulses with a duration of 4 μs spanning the 2-8 GHz frequency range were broadcast into the vacuum chamber through a horn antenna to interact with each molecular beam pulse. The chirped pulses were generated by a 25 GS/s arbitrary waveform generator and amplified by a 300 Watt traveling wave tube amplifier. Subsequently, the free induction decay (FID) signals of the macroscopic dipole moment of the ensemble of molecules were collected for 40 μs with another horn antenna on the opposite end of the vacuum chamber, then amplified, and recorded with a digital oscilloscope in the time domain. The signals were transformed to the frequency domain through Fourier transformation. A fast-frame data acquisition scheme was employed for the excitation and emission cycles per supersonic expansion to reduce measurement time and sample consumption and to accelerate the data processing of the oscilloscope. The spectra have an accuracy in the frequency measurement better than 15 kHz and a resolution better than 25 kHz. The isotopic substitution experiments were conducted under the same spectral measurement process, using a mixture sample of FA and FA-d_2 (DCOOD) with a 1:1 ratio. The spectra of singly ^{13}C substituted species were measured in the natural abundance (ca. 1.1%).

Spectral Fitting

The rotational constants of the optimized structures of each cluster class were used for initial assignment in the spectra using JB95[3] and a home-made autofitting program. The refining fit was performed with Pickett's SPFIT program in the AABS program package.[4,5] The isotopologues were fitted in the same manner.

Isomer abundances

For the observed isomers, their relative abundances can be estimated from the experimental rotational signal intensities. For a given transition, the observed line intensity *I* depends on the molecular population *N*, the square of the calculated dipole

moment components ($\mu^2_{a,b,c}$), and the transition-specific simulated intensity factor γ, which accounts for the transition probability and rotational line strength. By selecting transitions with similar frequencies, the relative population ratio between two species can be estimated as:

$$\frac{I_A}{I_B} \approx \frac{N_A \mu_A^2 \gamma_A}{N_B \mu_B^2 \gamma_B}$$

For the predicted low-lying isomers that are not experimentally observed, their relative abundances were estimated from the Boltzmann distribution:

$$\frac{N_i}{N_j} \approx g_i \exp\left(-\frac{\Delta E_{ij}}{kT}\right)$$

where ΔE_{ij} is the relative energy difference between isomers i and j, g_i is the corresponding degeneracy factor, k is the Boltzmann constant, and T is the temperature. The corresponding population predictions at different temperatures are summarized in Tables S6–S9.

Theoretical details

The Conformer-Rotamer Ensemble Sampling Tool (CREST) software is known for its efficiency in generating and analyzing structural ensembles.[6] For the PA_mFA_n clusters, multiple connectivity motifs were considered, including FA-FA···PA, PA-FA···FA, PA-FA···PA, (PA-FA)||(FA-FA), and (PA-FA)||(PA-FA) (see main text for an explanation of the nomenclature). Separate initial structures were constructed for each motif. Independent conformational searches were performed using the iMTD-GC workflow implemented in CREST at the GFN2-xTB level of theory. The simulations were conducted in the so-called NCI mode, in which an ellipsoidal confining potential is applied around the initial structure to prevent dissociation of the noncovalently bound clusters. All simulations were performed using the default CREST settings (SHAKE applied to all bonds; time step = 5 fs; T = 400 K; coordinate dump = 100 fs; bias update = 1.0 ps; energy window = 6.0 kcal/mol; all other parameters set to the default iMTD-GC protocol).

For each motif, the 30 lowest-energy structures were selected from the combined ensembles and subsequently optimized at the B3LYP-D4/def2-TZVP level[7–10] using the ORCA program[11]. Zero-point energy (ZPE) and basis set superposition error (BSSE) corrections were included for the relative energy evaluation. The interconversion barriers between representative low-energy conformers were calculated at the same level of theory using the nudged elastic band (NEB) method as implemented in ORCA. Unless otherwise specified, all results discussed in this work are based on the B3LYP-D4/def2-TZVP level. To obtain more accurate structural information, the experimentally assigned structures were re-optimized at several levels of theory, including revDSD-PBEP86-D3(BJ)/def2-TZVPP[9,12–14], B2PLYP-D3(BJ)/jun-cc-pVTZ[12,13,15,16] and MP2/aug-cc-pVTZ[17–19], using the Gaussian software package[20,21].

For the analysis of non-covalent intermolecular interactions, symmetry-adapted perturbation theory (SAPT) was employed.[22] SAPT applies inter-molecular perturbation theory to analyze the various contributions from the fragments of a complex, utilizing the Psi4 package.[23]

To intuitively reveal the characteristics of π-electron delocalization, the Localized Orbital Locator (LOL) specifically for π electrons (denoted as LOL-π) was calculated based on the original definition by Schmider and Becke.[24] The LOL-π analysis was performed specifically for the π electrons identified using the method proposed by Lu and Chen,[25] utilizing the Multiwfn program[26]. The resulting grid data were visualized using the VMD software[27].

To gain further insight into the nature of intermolecular interactions, we employed the many-body expansion (MBE) method, which systematically breaks down the total interaction energy of an n-body molecular system into contributions from 1-body, 2-body, 3-body, and higher-order interactions.[28] The total interaction energy of an *N*-molecule system is expressed as:

$$E_{int} = E_{iB} + E_{2B} + E_{3B} + \cdots + E_{NB}.$$

Here, E_{iB} denotes the total energy perturbation of all monomers, defined as the sum of the differences between the energy of each monomer i in the cluster configuration (E_{ci}) and its energy in the isolated gas-phase geometry (E_{mi}):

$$E_{iB} = \sum_{i}^{\mathrm{N}}(E_{ci} - E_{mi}).$$

For higher-order interaction terms ($E_{nB}, with$ n ≥ 2), the general form is

$$E_{nB} = \sum_{i>j>n}^{N}\left[E_c^{ij\cdots n} - \left(E_c^i + E_c^j + \cdots + E_c^n\right)\right] - \sum_{a=2}^{n-1} E_{aB}.$$

This recursive formulation captures the non-additive contributions of *n*-body interactions by subtracting all lower-order terms already included in the expansion. All many-body interaction energies reported in this work include zero-point energy (ZPE) corrections, ensuring a more accurate estimation of the interaction contributions under vibrationally averaged conditions.

Structural Determination

Both the r_0 and r_e^{SE} structures were determined by least-squares fitting using the STRFIT program.[5,29,30] For the r_e^{SE} analysis, the required vibrational corrections were included, with these corrections evaluated at the B3LYP-D3(BJ)/def2-TZVP level[7–9,12,13].

Figures

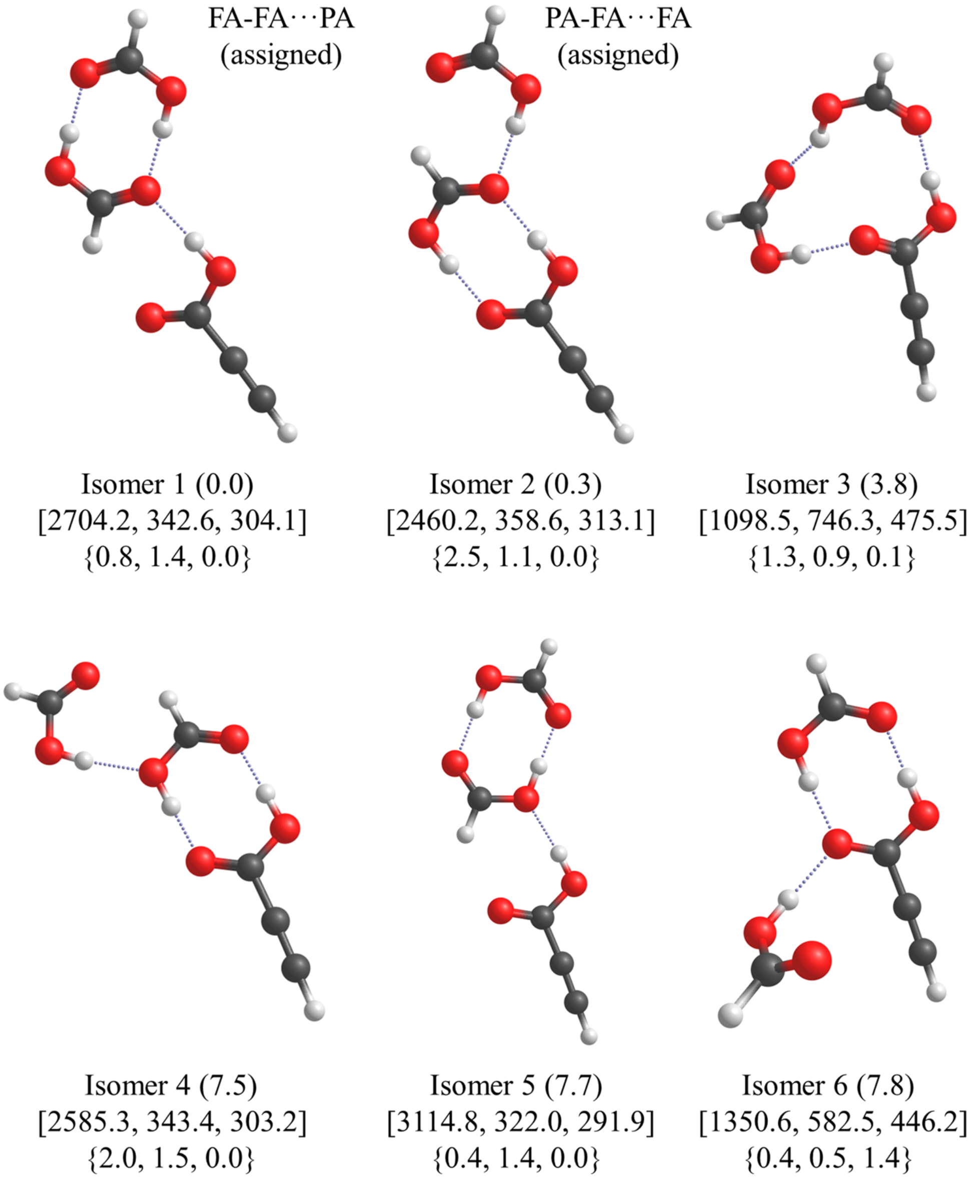

Figure S1. Low-energy isomers of $PAFA_2$. Values in parentheses give relative energies (kJ/mol, B3LYP-D4/def2-TZVP with ZPE and BSSE corrections), brackets list the rotational constants (*A*, *B*, *C* in MHz), and curly braces denote the dipole moment components (μ_a, μ_b, μ_c in Debye).

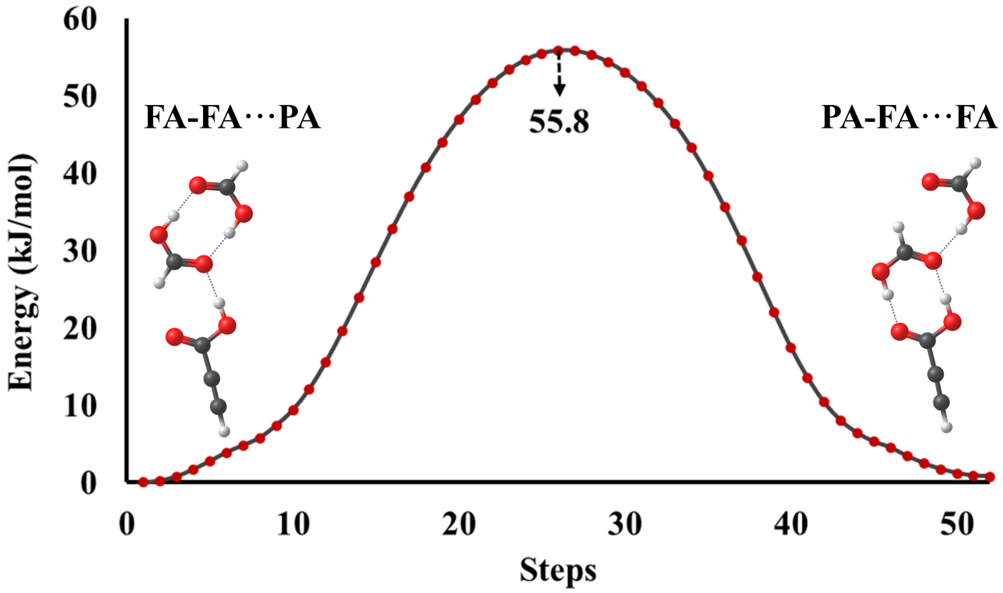

Figure S2. The conversion barrier between FA-FA···PA and PA-FA···FA calculated at the B3LYP-D4/def2-TZVP level.

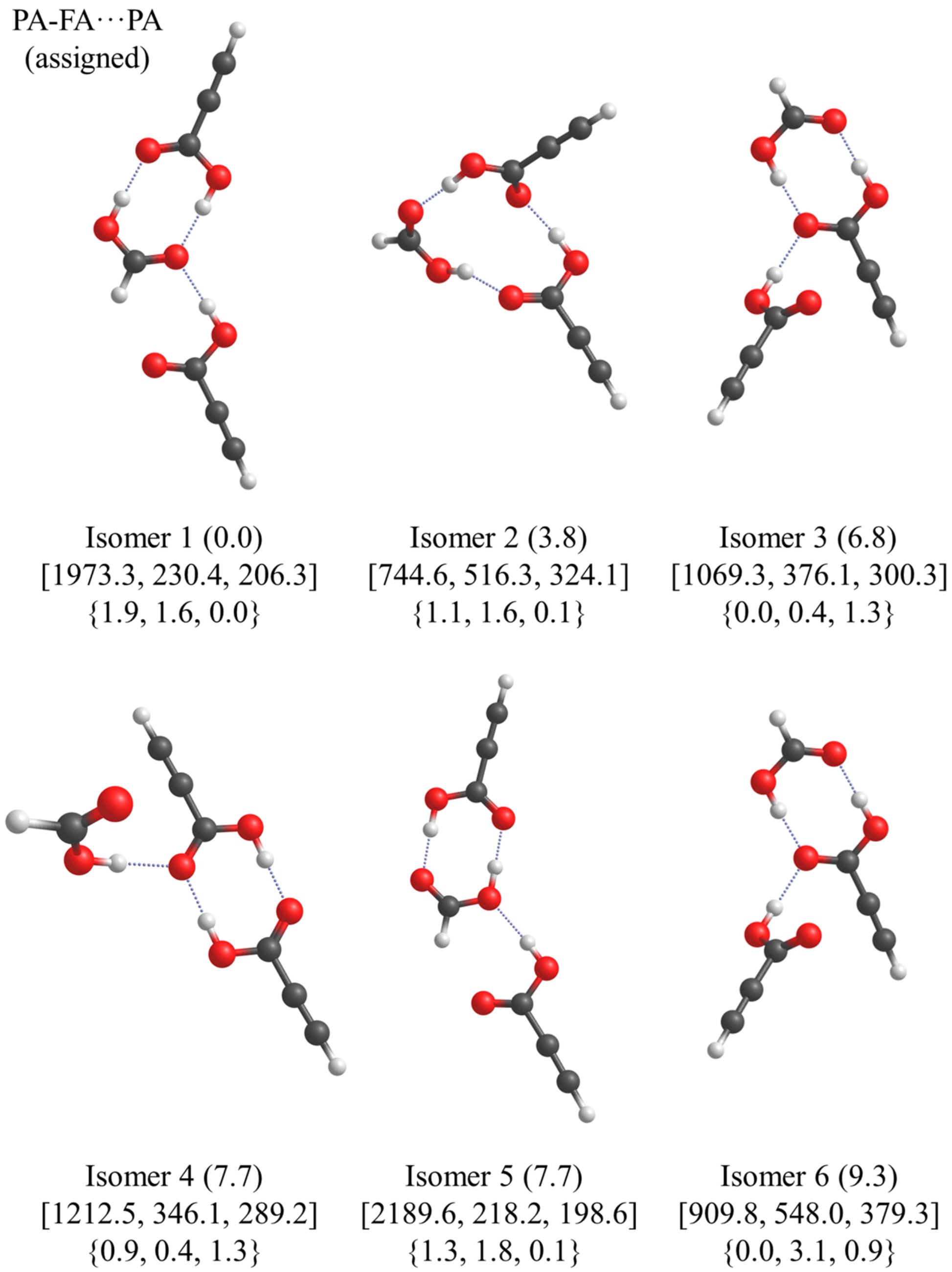

Figure S3. Low-energy isomers of PA_2FA. Values in parentheses give relative energies (kJ/mol, B3LYP-D4/def2-TZVP with ZPE and BSSE corrections), brackets list the rotational constants (A, B, C in MHz), and curly braces denote the dipole moment components (μ_a, μ_b, μ_c in Debye).

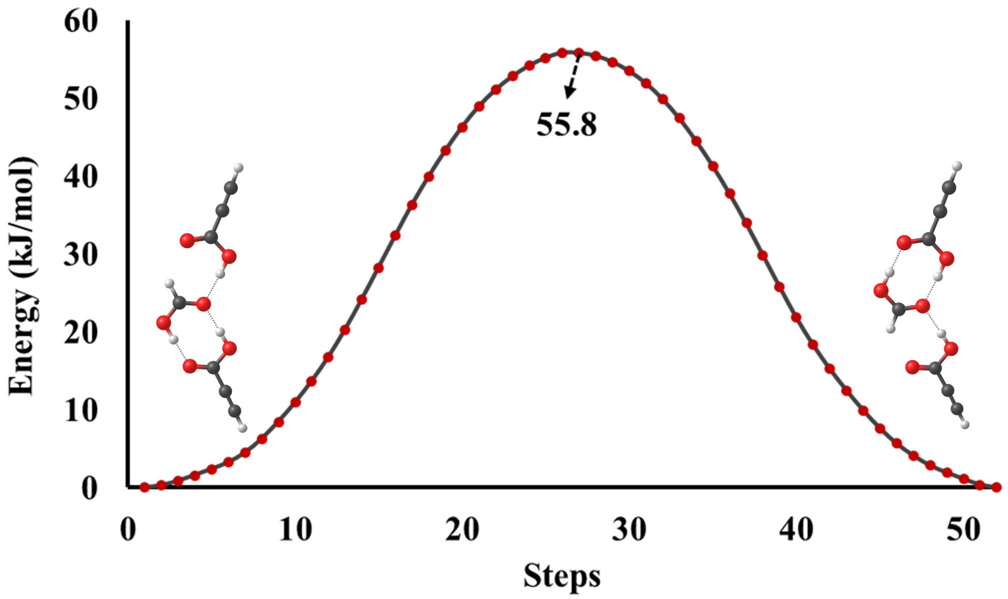

Figure S4. Rotation barrier of the FA unit in the PA-FA···PA cluster calculated at the B3LYP-D4/def2-TZVP level.

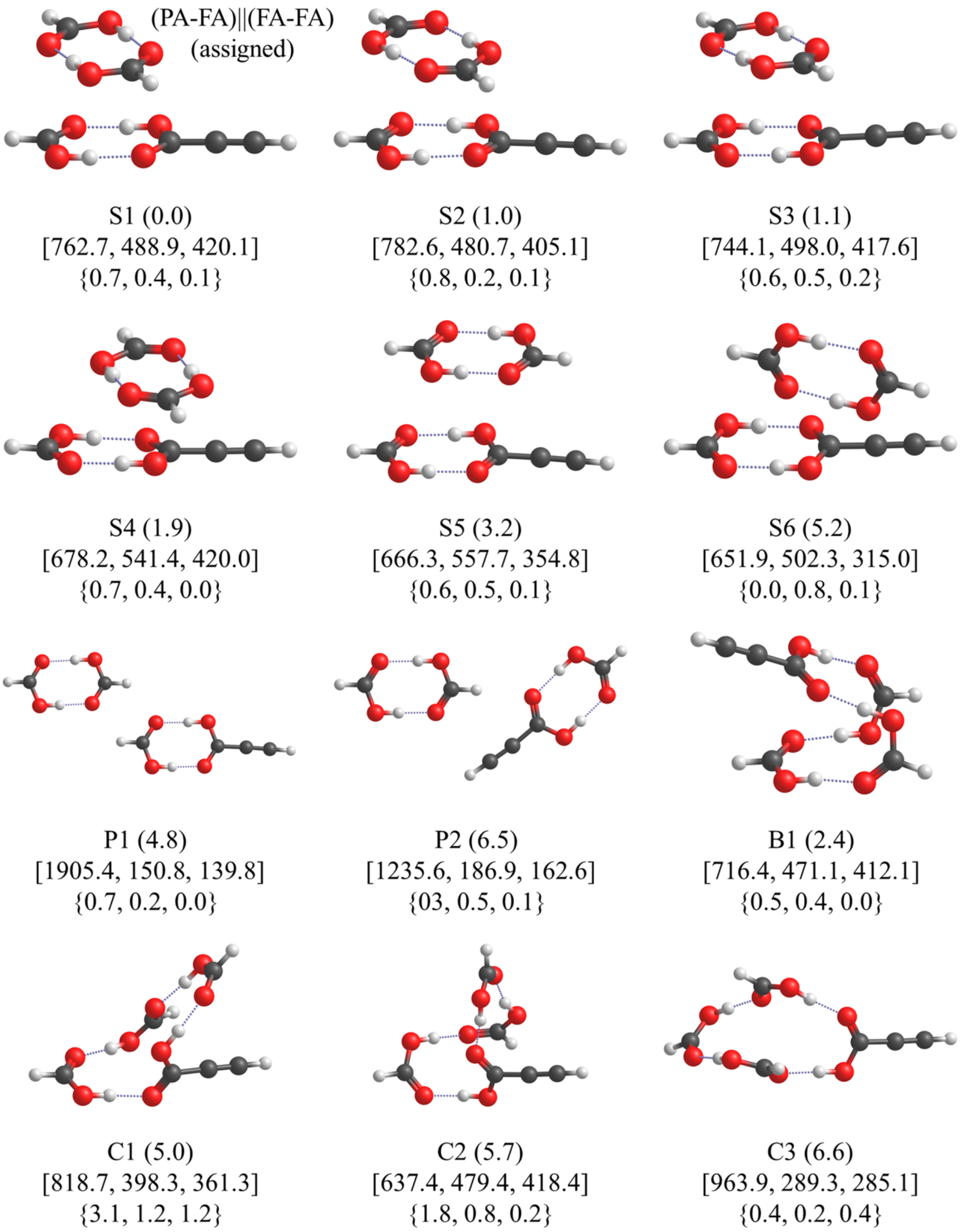

Figure S5. Low-energy isomers of $PAFA_3$: stacking (S), planar (P), butterfly (B), and cyclic (C) structures. These structures are named according to their classes, and followed by an Arabic numeral, which corresponds to their energy ordering within the respective classes, with 1 being the most stable structure. Values in parentheses give relative energies (kJ/mol, B3LYP-D4/def2-TZVP with ZPE and BSSE corrections), brackets list the rotational constants (A, B, C in MHz), and curly braces denote the dipole moment components (μ_a, μ_b, μ_c in Debye).

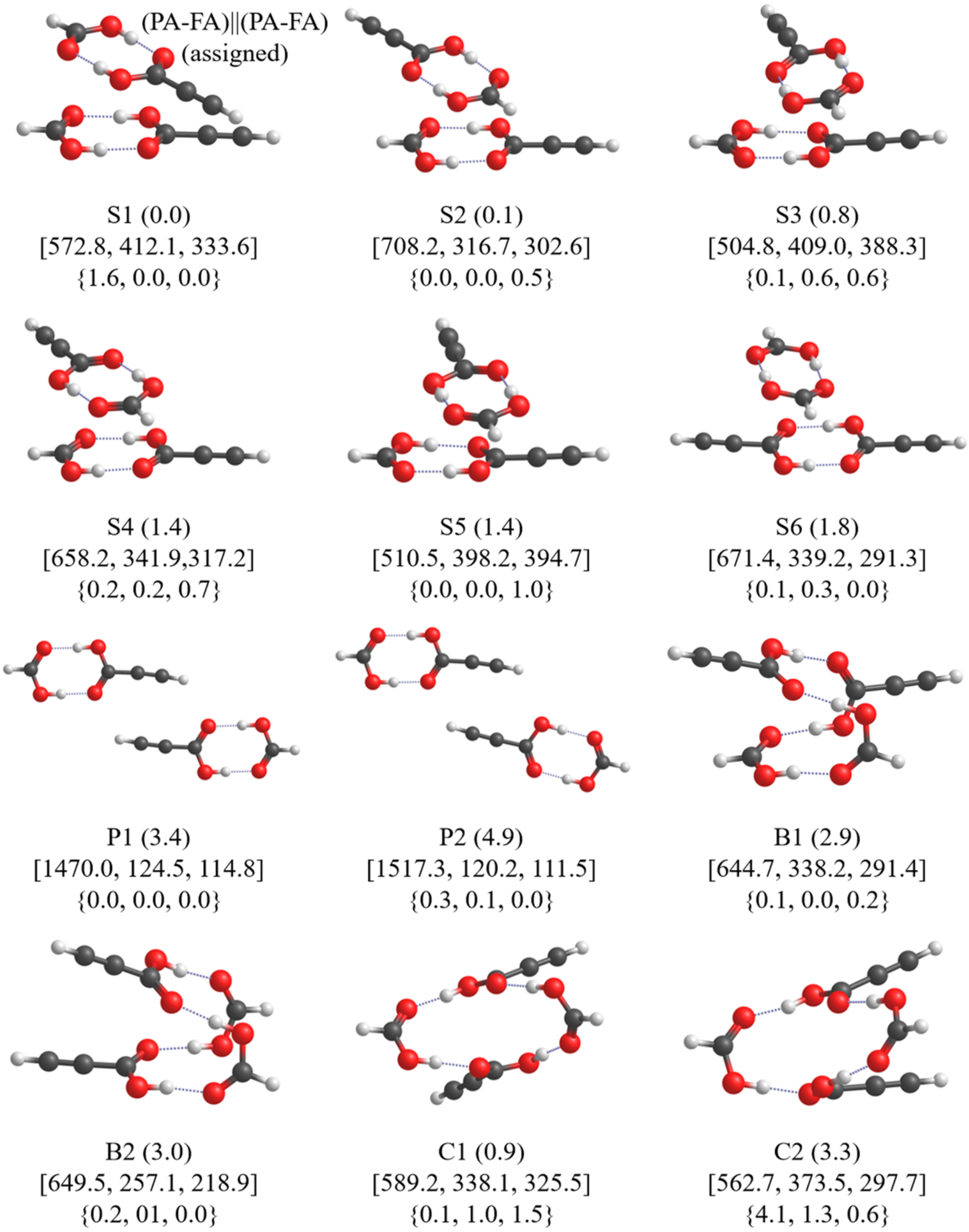

Figure S6. Low-energy isomers of PA_2FA_2: stacking (S), planar (P), butterfly (B), and cyclic (C) structures. These structures are named according to their classes, and followed by an Arabic numeral, which corresponds to their energy ordering within the respective classes, with 1 being the most stable structure. Values in parentheses give relative energies (kJ/mol, B3LYP-D4/def2-TZVP with ZPE and BSSE corrections), brackets list the rotational constants (A, B, C in MHz), and curly braces denote the dipole moment components (μ_a, μ_b, μ_c in Debye).

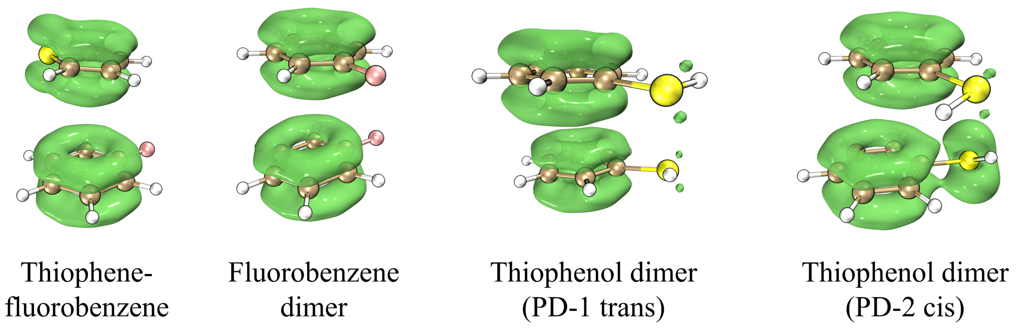

Figure S7. LOL-π isosurfaces of selected aromatic ring dimers (isovalue = 0.3).

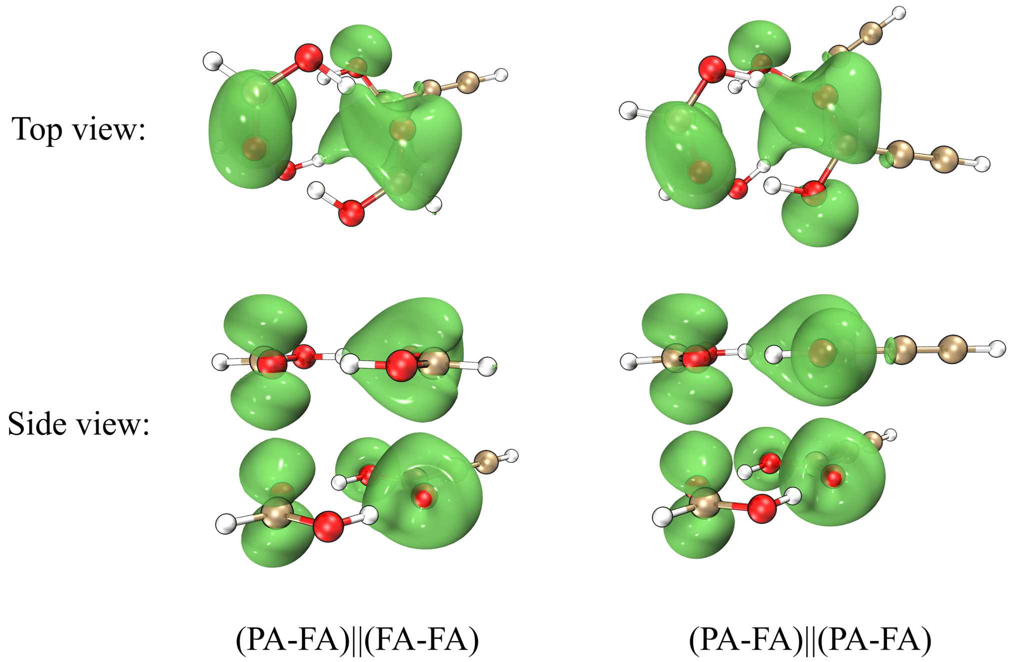

Figure S8. Top and side views of the LOL-π isosurfaces for (PA-FA)||(FA-FA) and (PA-FA)||(PA-FA) (isovalue = 0.15).

Tables

Table S1. Calculated spectroscopic parameters for the assigned PA_mFA_n clusters at the revDSD-PBEP86-D3(BJ)/def2-TZVPP level.

**Class**	**$PAFA_2$**		**PA_2FA**	**$PAFA_3$**	**PA_2FA_2**
**Species**	**FA-FA···PA**	**PA-FA···FA**	**PA-FA···PA**	**(PA-FA)‖(FA-FA)**	**(PA-FA)‖(PA-FA)**
A **[MHz]**	2691.4	2462.7	1979.5	758.4	571.8
B **[MHz]**	340.0	354.9	228.1	487.7	409.6
C **[MHz]**	301.9	310.2	204.5	417.6	334.9
D_J **[kHz]**	0.016	0.020	0.0078	0.18	-0.0010
D_{JK} **[kHz]**	-0.097	-0.30	-0.22	-0.11	0.83
D_K **[kHz]**	4.9	5.5	5.7	0.66	-0.59
d_1 **[kHz]**	-0.0027	-0.0040	-0.0015	-0.056	0.0066
d_2 **[kHz]**	-0.00022	-0.00020	-0.000057	-0.0057	-0.014
$\mu_a/\mu_b/\mu_c$ **[D]**	0.8/1.3/0.0	2.2/1.1/0.0	1.7/1.5/0.0	0.6/0.3/0.1	1.3/0.0/0.0

Table S2. Calculated spectroscopic parameters for the assigned PA_mFA_n clusters at the B2PLYP-D3(BJ)/jun-cc-pVTZ level.

**Class**	**$PAFA_2$**		**PA_2FA**	**$PAFA_3$**	**PA_2FA_2**
**Species**	**FA-FA···PA**	**PA-FA···FA**	**PA-FA···PA**	**(PA-FA)‖(FA-FA)**	**(PA-FA)‖(PA-FA)**
A **[MHz]**	2707.0	2473.2	1994.6	766.1	572.6
B **[MHz]**	341.7	356.9	229.2	491.1	411.8
C **[MHz]**	303.4	311.9	205.6	420.8	336.4
D_J **[kHz]**	0.015	0.019	0.0074	0.58	0.0038
D_{JK} **[kHz]**	-0.097	-0.29	-0.22	-0.35	0.66
D_K **[kHz]**	4.8	5.2	5.6	1.0	-0.43
d_1 **[kHz]**	-0.0026	-0.0039	-0.0015	-0.16	0.0065
d_2 **[kHz]**	-0.00021	-0.00019	-0.000055	-0.023	-0.011
$\mu_a/\mu_b/\mu_c$ **[D]**	0.8/1.4/0.0	2.4/1.1/0.0	1.7/1.6/0.0	0.7/0.3/0.1	1.5/0.0/0.0

Table S3. Calculated spectroscopic parameters for the assigned PA_mFA_n clusters at the B3LYP-D4/def2-TZVP level.

Class	$PAFA_2$		PA_2FA	$PAFA_3$	PA_2FA_2
Species	FA-FA···PA	PA-FA···FA	PA-FA···PA	(PA-FA)‖(FA-FA)	(PA-FA)‖(PA-FA)
A [MHz]	2704.2	2460.2	1973.3	762.7	572.5
B [MHz]	342.6	358.6	230.4	488.9	412.4
C [MHz]	304.1	313.1	206.3	420.1	334.2
D_J [kHz]	0.015	0.019	0.0063	0.17	0.0020
D_{JK} [kHz]	-0.13	-0.28	0.034	-0.11	0.72
D_K [kHz]	5.1	5.2	3.2	0.61	-0.49
d_1 [kHz]	-0.0025	-0.0037	-0.0010	-0.053	0.0056
d_2 [kHz]	-0.00018	-0.00018	-0.00016	-0.0053	-0.012
$\mu_a/\mu_b/\mu_c$ [D]	0.8/1.4/0.0	2.5/1.1/0.0	1.9/1.6/0.0	0.7/0.4/0.1	1.6/0.1/0.0

Table S4. Calculated spectroscopic parameters for the assigned PA_mFA_n clusters at the MP2/aug-cc-pVTZ level.

Class	$PAFA_2$		PA_2FA	$PAFA_3$	PA_2FA_2
Species	FA-FA···PA	PA-FA···FA	PA-FA···PA	(PA-FA)‖(FA-FA)	(PA-FA)‖(PA-FA)
A [MHz]	2678.0	2449.6	1967.8	779.4	607.5
B [MHz]	343.4	358.3	230.2	497.8	409.3
C [MHz]	304.3	312.6	206.1	430.0	345.7
D_J [kHz]	0.016	0.020	0.0077	0.13	0.0090
D_{JK} [kHz]	-0.10	-0.30	-0.22	-0.047	0.54
D_K [kHz]	4.7	5.3	5.5	0.49	-0.34
d_1 [kHz]	-0.0027	-0.0041	-0.0015	-0.039	0.0060
d_2 [kHz]	-0.00022	-0.00020	-0.000058	-0.0035	-0.0070
$\mu_a/\mu_b/\mu_c$ [D]	0.8/1.3/0.0	2.3/1.0/0.0	1.7/1.4/0.0	0.7/0.3/0.1	1.4/0.0/0.0

Table S5. Vibrational corrections of the parent and five ^{13}C isotopologues for FA-FA···PA used in the semi-experimental equilibrium structure analysis.

$\Delta B_{i,0}^{\mathrm{calc}\,a}$	Parent	$^{13}C_1$	$^{13}C_2$	$^{13}C_3$	$^{13}C_4$	$^{13}C_5$
***a* /MHz**	3.61556	6.12327	6.90692	6.92780	6.84022	6.90817
***b* /MHz**	2.95670	2.63901	2.63657	2.63001	2.64569	2.76252
***c* /MHz**[b]	2.27901	2.04427	2.04657	2.04437	2.05863	2.15566

[a]$\Delta B_{i,0}^{calc} = \frac{1}{2}\sum_n \alpha_i^n$, which is half the sum of the vibration-rotation interaction constants calculated at the B3LYP-D3(BJ)/def2-TZVP level of theory. Here, α_i^n denotes the vibration-rotation interaction constants, i denotes the principal inertial axis (a, b or c), and the sum is taken over all fundamental vibrational modes n. Note that $\Delta B_{i,0}^{calc}$ also corresponds to the difference between the semi-experimental equilibrium rotational constants and the experimental vibrational ground-state rotational constants ($\Delta B_{i,0}^{calc} = B_{i,e}^{SE} - B_{i,0}^{exp}$).

[b]The experimental rotational constants A and B and their corresponding calculated vibrational corrections are used to derive the semi-experimental equilibrium structure for the planar molecule ($\frac{1}{C_e} = \frac{1}{A_e} + \frac{1}{B_e}$).

d

Table S6. Vibrational corrections of the parent and five ^{13}C isotopologues for PA-FA···FA used in the semi-experimental equilibrium structure analysis.

$\Delta B_{i,0}^{\mathrm{calc}\,a}$	Parent	$^{13}C_1$	$^{13}C_2$	$^{13}C_3$	$^{13}C_4$	$^{13}C_5$
***a* /MHz**	9.72195	9.98665	9.33153	9.93870	9.88523	9.84679
***b* /MHz**	2.26207	2.21775	2.25371	2.22165	2.21972	2.19159
***c* /MHz**[b]	1.76021	1.73551	1.74845	1.73436	1.73410	1.71473

[a]$\Delta B_{i,0}^{calc} = \frac{1}{2}\sum_n \alpha_i^n$, which is half the sum of the vibration-rotation interaction constants calculated at the B3LYP-D3(BJ)/def2-TZVP level of theory. Here, α_i^n denotes the vibration-rotation interaction constants, i denotes the principal inertial axis (a, b or c), and the sum is taken over all fundamental vibrational modes n. Note that $\Delta B_{i,0}^{calc}$ also corresponds to the difference between the semi-experimental equilibrium rotational constants and the experimental vibrational ground-state rotational constants ($\Delta B_{i,0}^{calc} = B_{i,e}^{SE} - B_{i,0}^{exp}$).

[b]The experimental rotational constants A and B and their corresponding calculated vibrational corrections are used to derive the semi-experimental equilibrium structure for the planar molecule ($\frac{1}{C_e} = \frac{1}{A_e} + \frac{1}{B_e}$).

Table S7. Boltzmann populations (%) for $PAFA_2$.

T/K	Isomer1	Isomer2	Isomer3	Isomer4	Isomer5	Isomer6
10	97.4	2.6	<0.01	<0.01	<0.01	<0.01
50	67.3	32.7	<0.01	<0.01	<0.01	<0.01
100	58.6	40.8	0.6	<0.01	<0.01	<0.01
250	47.6	41.2	7.6	1.3	1.2	1.1
300	44.6	39.5	9.7	2.2	2.0	2.0

Table S8. Boltzmann populations (%) for PA_2FA.

T/K	Isomer1	Isomer2	Isomer3	Isomer4	Isomer5	Isomer6
10	100.0	<0.1	<0.1	<0.1	<0.1	<0.1
50	100.0	<0.1	<0.1	<0.1	<0.1	<0.1
100	98.9	1.0	<0.1	<0.1	<0.1	<0.1
250	79.4	12.8	3.0	2.0	2.0	0.9
300	71.5	15.6	4.7	3.3	3.3	1.7

Table S9. Boltzmann populations (%) for $PAFA_3$.

T/K	S1	S2	S3	S4	S5	S6	P1	P2	B1	C1	C2	C3
10	100.0	<0.1	<0.1	<0.1	<0.1	<0.1	<0.1	<0.1	<0.1	<0.1	<0.1	<0.1
50	85.1	7.7	6.0	0.9	<0.1	<0.1	<0.1	<0.1	0.3	<0.1	<0.1	<0.1
100	57.0	17.1	15.2	5.8	1.2	0.1	0.2	<0.1	3.2	0.1	<0.1	<0.1
250	28.1	17.4	16.6	11.3	6.0	2.3	2.8	1.2	8.9	2.5	1.8	1.2
300	24.4	16.4	15.7	11.4	6.8	3.0	3.6	1.8	9.3	3.3	2.5	1.7

Table S10. Boltzmann populations (%) for PA_2FA_2.

T/K	S1	S2	S3	S4	S5	S6	P1	P2	B1	C1	C2	C3
10	76.9	23.1	<0.1	<0.1	<0.1	<0.1	<0.1	<0.1	<0.1	<0.1	<0.1	<0.1
50	46.9	36.9	6.8	1.6	1.6	0.6	<0.1	<0.1	0.3	<0.1	<0.1	<0.1
100	31.4	27.8	12.0	5.8	5.8	3.6	0.5	<0.1	1.0	0.8	10.6	0.6
250	17.5	16.7	11.9	8.9	8.9	7.4	3.4	1.7	4.3	4.1	11.4	3.6
300	15.9	15.3	11.5	9.1	9.1	7.7	4.1	2.2	5.0	4.8	11.1	4.2

Table S11. Results of many-body expansion analysis, in kJ/mol.

Calculation					
**Class**	**$PAFA_2$**		**PA_2FA**	**$PAFA_3$**	**PA_2FA_2**
**Species**	**FA-FA···PA (isomer 1)**	**PA-FA···FA (isomer 2)**	**PA-FA···PA**	**(PA-FA)‖(FA-FA)**	**(PA-FA)‖(PA-FA)**
E_{1B}	19.67	19.40	20.01	29.70	30.37
E_{2B}	-122.71	-122.30	-125.81	-180.06	-187.02
E_{3B}	-2.27	-1.96	-1.31	-13.12	-9.29
E_{4B}	-	-	-	5.05	3.95
E_{int}	-124.97	-124.27	-127.12	-188.13	-192.37

Table S12. Results of two-body SAPT energy decomposition calculations at the SAPT2+(3)δ(MP2)/aug-cc-pVTZ level, in kJ/mol.

**Species**	**Thiophene-fluorobenzene**	**Fluorobenzene dimer**	**PD-1 trans**
**Exchange**	30.93	31.82	54.60
**Electrostatics**	-10.95	-11.48	-24.90
**Induction**	-1.98	-2.00	-7.70
**Dispersion**	-32.76	-35.41	-47.90
$E_{repulsion}$	30.93	31.82	54.60
$E_{attractive}$	-45.69	-48.89	-80.50
E_{total}	-14.76	-17.07	-25.90
**Species**	**PD-2 cis**	**(PA-FA)‖(FA-FA)**	**(PA-FA)‖(PA-FA)**
**Exchange**	61.00	7.70	8.10
**Electrostatics**	-26.20	-4.50	-4.20
**Induction**	-8.40	-0.80	-0.80
**Dispersion**	-53.3	-7.90	-8.90
$E_{repulsion}$	61.00	7.70	8.10
$E_{attractive}$	-87.90	-13.2	-13.90
E_{total}	-26.90	-5.50	-5.80

Table S13. Experimental spectroscopic parameters for singly ^{13}C-substituted isotopologues of the FA-FA···PA cluster.

Species	^{13}C1	^{13}C2	^{13}C3	^{13}C4	^{13}C5
A [MHz]	2662.5951(34)[a]	2655.3691(24)	2676.0260(28)	2674.2564(33)	2670.4147(36)
B [MHz]	336.51186(25)	339.88876(19)	338.39642(25)	336.26704(15)	333.84794(24)
C [MHz]	298.98888(18)	301.55875(11)	300.64581(15)	298.94277(15)	296.98148(15)
D_J [kHz]	[0.01766][b]	[0.01766]	[0.01766]	[0.01766]	[0.01766]
D_{JK} [kHz]	[-0.1287]	[-0.1287]	[-0.1287]	[-0.1287]	[-0.1287]
D_K [kHz]	[5.546]	[5.546]	[5.546]	[5.546]	[5.546]
d_1 [kHz]	[-0.002935]	[-0.002935]	[-0.002935]	[-0.002935]	[-0.002935]
d_2 [kHz]	[-0.000271]	[-0.000271]	[-0.000271]	[-0.000271]	[-0.000271]
$\mu_a/\mu_b/\mu_c$ [D]	Y/Y/N[c]	Y/Y/N	Y/Y/N	Y/Y/N	Y/Y/N
N[d]	17	23	20	25	21
σ [kHz][e]	4.9	4.3	4.2	4.5	5.9

[a]Standard error within parentheses are expressed in units of the last two digits. [b]The values in square brackets were fixed to those of the normal species. [c]Y and N denote that the corresponding transitions were observed or not observed. [d]Number of the lines in the fit. [e]Root-mean-square deviation of the fit.

Table S14. Experimental spectroscopic parameters for singly deuterium-substituted isotopologues of the FA-FA···PA cluster.

Species	FACD-FA ···PA (D1)	FAOD-FA ···PA (D2)	FA-FACD ···PA (D3)	FA-FAOD ···PA (D4)	FA-FA ···PAOD (D5)
A [MHz]	2641.0416(33)[a]	2654.5569(14)	2631.9478(20)	2647.2451(12)	2669.1200(33)
B [MHz]	334.72875(17)	339.02711(26)	340.03183(18)	338.31549(27)	339.81358(17)
C [MHz]	297.31375(19)	300.87255(25)	301.36785(17)	300.21790(25)	301.68018(24)
D_J [kHz]	0.01747(76)	0.0189(15)	0.01596(83)	0.0159(13)	0.01657(96)
D_{JK} [kHz]	-0.123(15)		-0.127(10)		-0.176(32)
D_K [kHz]	4.77(69)		6.47(43)		
$\mu_a/\mu_b/\mu_c$ [D]	Y/Y/N[b]	Y/Y/N	Y/Y/N	Y/Y/N	Y/Y/N
N[c]	75	40	89	49	48
σ [kHz][d]	7.8	6.8	7.4	8.0	6.3

[a]Standard error within parentheses are expressed in units of the last two digits. [b]Y and N denote that the corresponding transitions were observed or not observed. [c]Number of the lines in the fit. [d]Root-mean-square deviation of the fit.

Table S15. Experimental spectroscopic parameters for double deuterium-substituted isotopologues of the FA-FA⋯PA cluster.

**Species**	**D1 & D2**	**D1 & D3**	**D1 & D4**	**D1 & D5**
***A* [MHz]**	2620.7926(28)[a]	2597.8551(18)	2612.1527(15)	2634.3966(22)
***B* [MHz]**	333.77792(22)	334.72959(17)	333.11037(34)	334.51276(33)
***C* [MHz]**	296.30724(23)	296.75778(15)	295.67088(28)	297.06305(29)
D_J **[kHz]**	0.0131(13)	0.01814(65)	0.0199(18)	0.0179(15)
D_{JK} **[kHz]**	-	-0.1107(66)	-	0.119(36)
D_K **[kHz]**	5.23(56)	7.23(37)	-	-
d_1 **[kHz]**	-	-0.00414(37)	-	-
$\mu_a/\mu_b/\mu_c$ **[D]**	Y/Y/N[b]	Y/Y/N	Y/Y/N	Y/Y/N
N[c]	44	92	44	36
σ **[kHz]**[d]	6.0	6.4	7.8	7.8
**Species**	**D2 & D3**	**D3 & D4**	**D3 & D5**	
***A* [MHz]**	2610.8312(12)	2604.4139(15)	2625.0309(15)	
***B* [MHz]**	339.03031(24)	338.32193(28)	339.81852(29)	
***C* [MHz]**	300.30628(22)	299.66467(29)	301.11349(26)	
D_J **[kHz]**	0.0213(10)	0.0211(17)	0.0162(14)	
$\mu_a/\mu_b/\mu_c$ **[D]**	Y/Y/N	Y/Y/N	Y/Y/N	
N	49	41	38	
σ **[kHz]**	7.6	7.5	7.7	

[a]Standard error within parentheses are expressed in units of the last two digits. [b]Y and N denote that the corresponding transitions were observed or not observed. [c]Number of the lines in the fit. [d]Root-mean-square deviation of the fit.

Table S16. Experimental spectroscopic parameters for singly ^{13}C-substituted isotopologues of the PA-FA···FA cluster.

Species	^{13}C1	^{13}C2	^{13}C3	^{13}C4	^{13}C5
A [MHz]	2431.649(66)[a]	2422.296(70)	2439.2576(30)	2435.5053(28)	2429.2842(34)
B [MHz]	350.96379(21)	354.97495(20)	353.71089(16)	351.56609(16)	349.09291(18)
C [MHz]	306.92161(20)	309.83083(19)	309.14248(12)	307.44278(11)	305.45215(14)
D_J [kHz]	[0.02238][b]	[0.02238]	[0.02238]	[0.02238]	[0.02238]
D_{JK} [kHz]	[-0.3343]	[-0.3343]	[-0.3343]	[-0.3343]	[-0.3343]
D_K [kHz]	[5.87]	[5.87]	[5.87]	[5.87]	[5.87]
d_1 [kHz]	[-0.00447]	[-0.00447]	[-0.00447]	[-0.00447]	[-0.00447]
$\mu_a/\mu_b/\mu_c$ [D]	Y/N/N[c]	Y/N/N[c]	Y/Y/N[c]	Y/Y/N[c]	Y/Y/N[c]
N[d]	26	33	30	34	34
σ [kHz][e]	5.8	5.9	4.7	4.9	5.5

[a]Standard error within parentheses are expressed in units of the last two digits. [b]The values in square brackets were fixed to those of the normal species. [c]Y and N denote that the corresponding transitions were observed or not observed. [d]Number of the lines in the fit. [e]Root-mean-square deviation of the fit.

Table S17. Experimental spectroscopic parameters for singly deuterium-substituted isotopologues of the PA-FA⋯FA cluster.

Species	PA-FA ⋯FACD (D1)	PA-FA ⋯FAOD (D2)	PA-FACD ⋯FA (D3)	PA-FAOD ⋯FA (D4)	PAOD-FA ⋯FA (D5)
$\boldsymbol{A}$ [MHz]	2417.9235(27)[a]	2424.1173(43)	2413.8503(21)	2399.3813(46)	2439.2312(10)
$\boldsymbol{B}$ [MHz]	348.80896(18)	354.02682(25)	354.10456(14)	354.95865(28)	355.26360(10)
$\boldsymbol{C}$ [MHz]	305.05894(17)	309.14287(28)	309.03109(13)	309.44392(27)	310.327001(88)
$\boldsymbol{D_J}$ [kHz]	0.02042(55)	0.0201(11)	0.02213(48)	0.02150(97)	0.02354(40)
$\boldsymbol{D_{JK}}$ [kHz]	-0.3087(86)	-0.290(31)	-0.3126(73)	-0.368(48)	-0.3328(50)
$\boldsymbol{D_K}$ [kHz]	6.42(54)	-	5.77(42)	-	5.75(22)
$\boldsymbol{d_1}$ [kHz]	-0.00479(50)	-	-0.00482(37)	-	-0.00497(17)
$\boldsymbol{\mu_a/\mu_b/\mu_c}$ [D]	Y/Y/N[b]	Y/Y/N	Y/Y/N	Y/Y/N	Y/Y/N
N[c]	75	46	81	43	89
$\boldsymbol{\sigma}$ [kHz][d]	5.2	7.2	4.5	6.6	3.7

[a]Standard error within parentheses are expressed in units of the last two digits. [b]Y and N denote that the corresponding transitions were observed or not observed. [c]Number of the lines in the fit. [d]Root-mean-square deviation of the fit.

Table S18. Experimental spectroscopic parameters for double deuterium-substituted isotopologues of the PA-FA···FA cluster.

Species	D1 & D2	D1 & D3	D1 & D4
A [MHz]	2403.308(74)[a]	2392.3305(15)	2378.278(71)
B [MHz]	347.64685(28)	347.72881(21)	348.50296(29)
C [MHz]	303.94565(33)	303.82804(17)	304.19230(32)
D_J [kHz]	0.0201(12)	0.02185(77)	0.0192(11)
D_{JK} [kHz]	-	-0.322(18)	-
$\mu_a/\mu_b/\mu_c$ [D]	Y/N/N[b]	Y/Y/N	Y/N/N
N[c]	30	59	36
σ [kHz][d]	6.1	6.4	7.1

[a]Standard error within parentheses are expressed in units of the last two digits. [b]Y and N denote that the corresponding transitions were observed or not observed. [c]Number of the lines in the fit. [d]Root-mean-square deviation of the fit.

Table S19. Experimental spectroscopic parameters for singly deuterium-substituted isotopologues of the PA-FA⋯PA cluster.

**Species**	**PA-FA ⋯PAOD (D1)**	**PA-FACD ⋯PA (D2)**	**PA-FAOD ⋯PA (D3)**	**PAOD-FA ⋯PA (D4)**
$\boldsymbol{A}$ **[MHz]**	1967.08833(90)[a]	1944.8753(16)	1942.6564(14)	1966.1800(27)
$\boldsymbol{B}$ **[MHz]**	228.12632(12)	228.200456(58)	227.99919(12)	228.08845(11)
$\boldsymbol{C}$ **[MHz]**	204.54778(12)	204.361007(52)	204.17725(10)	204.504197(98)
$\boldsymbol{D_J}$ **[kHz]**	0.00907(26)	0.008961(100)	0.00821(25)	0.00876(19)
$\boldsymbol{D_{JK}}$ **[kHz]**	-0.262(11)	-0.2407(27)	-0.208(12)	-0.2332(70)
$\boldsymbol{D_K}$ **[kHz]**	-	6.72(32)	-	7.34(53)
$\boldsymbol{d_1}$ **[kHz]**	-	-0.001747(63)	-	-0.00196(14)
$\boldsymbol{\mu_a/\mu_b/\mu_c}$ **[D]**	Y/Y/N[b]	Y/Y/N	Y/Y/N	Y/Y/N
N[c]	87	148	86	103
$\boldsymbol{\sigma}$ **[kHz]**[d]	7.5	4.3	7.2	6.1

[a]Standard error within parentheses are expressed in units of the last two digits. [b]Y and N denote that the corresponding transitions were observed or not observed. [c]Number of the lines in the fit. [d]Root-mean-square deviation of the fit.

Table S20. Experimental transition frequencies (v/MHz) together with the corresponding observed - calculated differences (Δv/MHz) for the parent FA-FA···PA complex.

J′	K_a′	K_c′	*J*	K_a	K_c	*v*/MHz	Δ*v*/MHz
3	0	3	2	0	2	1924.0270	0.0013
4	0	4	3	0	3	2563.2124	0.0001
5	0	5	4	0	4	3200.5617	0.0002
6	0	6	5	0	5	3835.6329	-0.0025
7	0	7	6	0	6	4468.0242	-0.0017
8	0	8	7	0	7	5097.3720	0.0005
9	0	9	8	0	8	5723.3888	0.0079
10	0	10	9	0	9	6345.8557	-0.0020
11	0	11	10	0	10	6964.7395	0.0163
12	0	12	11	0	11	7580.0229	-0.0080
13	0	13	12	0	12	8191.9795	0.0082
3	1	3	2	1	2	1868.4442	-0.0004
3	1	2	2	1	1	1982.7272	0.0003
4	1	4	3	1	3	2490.7232	-0.0035
4	1	3	3	1	2	2643.0903	0.0042
5	1	5	4	1	4	3112.5637	0.0010
5	1	4	4	1	3	3302.9604	-0.0029
6	1	6	5	1	5	3733.8552	0.0015
6	1	5	5	1	4	3962.2229	-0.0023
7	1	7	6	1	6	4354.5042	-0.0052
7	1	6	6	1	5	4620.7338	0.0051
8	1	8	7	1	7	4974.4515	0.0019
8	1	7	7	1	6	5278.3212	0.0011
9	1	9	8	1	8	5593.6024	-0.0020
9	1	8	8	1	7	5934.8277	-0.0053
10	1	10	9	1	9	6211.9288	0.0121
10	1	9	9	1	8	6590.0811	-0.0060
11	1	11	10	1	10	6829.3306	-0.0107
11	1	10	10	1	9	7243.8980	0.0126
4	2	3	3	2	2	2567.4791	0.0046
4	2	2	3	2	1	2572.0960	0.0041
5	2	4	4	2	3	3208.7640	0.0018
5	2	3	4	2	2	3217.9837	-0.0006
6	2	5	5	2	4	3849.6639	0.0011
6	2	4	5	2	3	3865.7589	-0.0032
7	2	6	6	2	5	4490.0989	-0.0003
7	2	5	6	2	4	4515.7558	0.0002
8	2	7	7	2	6	5129.9874	-0.0074
8	2	6	7	2	5	5168.2597	0.0126

9	2	8	8	2	7	5769.2747	0.0015
9	2	7	8	2	6	5823.4536	0.0052
10	2	9	9	2	8	6407.8604	0.0016
10	2	8	9	2	7	6481.4756	0.0002
11	2	10	10	2	9	7045.6767	-0.0005
11	2	9	10	2	8	7142.3255	0.0005
12	2	11	11	2	10	7682.6502	-0.0057
12	2	10	11	2	9	7805.8522	-0.0070
5	3	3	4	3	2	3211.3472	-0.0003
5	3	2	4	3	1	3211.4225	0.0013
6	3	4	5	3	3	3854.1978	0.0034
6	3	3	5	3	2	3854.3912	0.0006
7	3	5	6	3	4	4497.3283	0.0033
7	3	4	6	3	3	4497.7670	0.0011
8	3	6	7	3	5	5140.7639	0.0026
8	3	5	7	3	4	5141.6414	-0.0010
9	3	7	8	3	6	5784.5142	0.0000
9	3	6	8	3	5	5786.1272	-0.0002
10	3	8	9	3	7	6428.5753	-0.0075
10	3	7	9	3	6	6431.3445	0.0013
11	3	9	10	3	8	7072.9563	0.0030
11	3	8	10	3	7	7077.4320	0.0045
5	4	2	4	4	1	3210.8304	-0.0011
5	4	1	4	4	0	3210.8304	-0.0011
6	4	3	5	4	2	3853.3354	0.0035
6	4	2	5	4	1	3853.3354	0.0035
7	4	4	6	4	3	4496.0148	0.0001
7	4	3	6	4	2	4496.0148	0.0001
9	4	6	8	4	5	5782.0454	-0.0040
9	4	5	8	4	4	5782.0454	-0.0040
11	4	8	10	4	7	7069.1463	0.0034
11	4	7	10	4	6	7069.2315	0.0131
12	4	9	11	4	8	7713.1736	0.0093
7	5	2	6	5	1	4495.3687	-0.0014
7	5	3	6	5	2	4495.3687	-0.0014
8	5	3	7	5	2	5137.9387	-0.0036
8	5	4	7	5	3	5137.9387	-0.0036
9	5	5	8	5	4	5780.6537	-0.0115
9	5	4	8	5	3	5780.6537	-0.0115
11	5	7	10	5	6	7066.6494	0.0111
11	5	6	10	5	5	7066.6494	0.0111
1	1	1	0	0	0	2977.8816	-0.0007
2	1	2	1	0	1	3581.7431	-0.0024

3	1	3	2	0	2	4166.7425	0.0067
4	1	4	3	0	3	4733.4369	0.0000
5	1	5	4	0	4	5282.7888	0.0016
6	1	6	5	0	5	5816.0807	0.0011
7	1	7	6	0	6	6334.9527	-0.0007
8	1	8	7	0	7	6841.3790	0.0019
9	1	9	8	0	8	7337.6079	-0.0022
10	1	10	9	0	9	7826.1378	-0.0080
11	1	11	10	0	10	8309.6197	-0.0096
12	1	12	11	0	11	8790.7538	0.0004
6	0	6	5	1	5	1753.4093	-0.0003
7	0	7	6	1	6	2487.5869	0.0050
8	0	8	7	1	7	3230.4441	0.0002
9	0	9	8	1	8	3979.3729	-0.0022
10	0	10	9	1	9	4731.6270	-0.0014
11	0	11	10	1	10	5484.4307	-0.0043
12	0	12	11	1	11	6235.1126	-0.0120
13	0	13	12	1	12	6981.2573	0.0084
14	0	14	13	1	13	7720.6939	0.0018
2	2	1	1	1	0	8329.7126	-0.0062
3	2	2	2	1	1	8933.5830	-0.0002
2	2	0	1	1	1	8368.2732	-0.0032
13	1	12	12	2	11	2916.0784	0.0080
14	1	13	13	2	12	3791.6658	-0.0031
1	1	0	1	0	1	2374.0199	0.0006
2	1	1	2	0	2	2412.5786	0.0012
3	1	2	3	0	3	2471.2766	-0.0018
4	1	3	4	0	4	2551.1538	0.0015
5	1	4	5	0	5	2653.5589	0.0046
6	1	5	6	0	6	2780.1433	-0.0006
7	1	6	7	0	7	2932.8466	0.0000
8	1	7	8	0	8	3113.7918	-0.0034
9	1	8	9	0	9	3325.2475	0.0000
10	1	9	10	0	10	3569.4749	-0.0019
11	1	10	11	0	11	3848.6388	-0.0002
12	1	11	12	0	12	4164.6225	0.0002
13	1	12	13	0	13	4518.8958	0.0022
14	1	13	14	0	14	4912.3556	-0.0013
15	1	14	15	0	15	5345.2431	0.0017
16	1	15	16	0	16	5817.0324	0.0042
17	1	16	17	0	17	6326.4261	-0.0033
19	1	18	19	0	19	7449.2663	-0.0008
16	2	14	16	1	15	5910.7024	-0.0130

15	2	13	15	1	14	5914.1406	-0.0065
17	2	15	17	1	16	5937.9448	0.0010
14	2	12	14	1	13	5944.9517	-0.0026
18	2	16	18	1	17	5998.7983	-0.0055
13	2	11	13	1	12	5999.5323	0.0009
12	2	10	12	1	11	6073.9763	0.0035
19	2	17	19	1	18	6095.9805	0.0028
11	2	9	11	1	10	6164.1276	-0.0001
10	2	8	10	1	9	6265.6866	-0.0015
9	2	7	9	1	8	6374.3031	0.0031
8	2	6	8	1	7	6485.6850	0.0003
7	2	5	7	1	6	6595.7573	-0.0002
6	2	4	6	1	5	6700.7333	0.0027
5	2	3	5	1	4	6797.1902	-0.0032
4	2	2	4	1	3	6882.1742	0.0016
3	2	1	3	1	2	6953.1698	0.0030
2	2	0	2	1	1	7008.1637	-0.0048
2	2	1	2	1	2	7121.9907	-0.0018
3	2	2	3	1	3	7179.4222	-0.0026
4	2	3	4	1	4	7256.1812	0.0087
5	2	4	5	1	5	7352.3897	0.0177
6	2	5	6	1	6	7468.1848	0.0035
7	2	6	7	1	7	7603.7688	-0.0022
8	2	7	8	1	8	7759.3128	-0.0035
9	2	8	9	1	9	7934.9764	-0.0085
10	2	9	10	1	10	8130.9104	-0.0166
12	2	11	12	1	12	8584.0692	-0.0027
6	2	5	7	1	6	2047.7536	0.0010
5	2	4	6	1	5	2818.8189	0.0005
4	2	3	5	1	4	3572.2805	-0.0009
3	2	2	4	1	3	4307.7693	-0.0010
2	2	1	3	1	2	5024.9786	-0.0008
8	2	6	9	1	9	2261.8731	0.0033
7	2	5	8	1	8	2687.2287	0.0016
6	2	4	7	1	7	3145.9220	0.0009
5	2	3	6	1	6	3634.6721	0.0038
4	2	2	5	1	5	4150.5363	-0.0012
3	2	1	4	1	4	4691.0088	0.0003
2	2	0	3	1	3	5254.0121	0.0021
13	3	11	14	2	12	2206.5248	0.0054
12	3	10	13	2	11	2983.7910	0.0039
11	3	9	12	2	10	3737.9947	0.0048
10	3	8	11	2	9	4470.8945	-0.0013

9	3	7	10	2	8	5184.6356	-0.0022
8	3	6	9	2	7	5881.6015	0.0025
7	3	5	8	2	6	6564.2900	0.0039
6	3	4	7	2	5	7235.2094	0.0012
5	3	3	6	2	4	7896.7579	-0.0115
14	3	11	15	2	14	2451.7611	0.0040
13	3	10	14	2	13	3017.0991	0.0088
12	3	9	13	2	12	3598.0696	0.0040
11	3	8	12	2	11	4192.2550	0.0004
10	3	7	11	2	10	4797.5010	0.0179
9	3	6	10	2	9	5411.8170	-0.0001
8	3	5	9	2	8	6033.5519	0.0034
7	3	4	8	2	7	6661.1763	-0.0028
6	3	3	7	2	6	7293.3988	-0.0092
5	3	2	6	2	5	7929.1129	-0.0040
16	4	13	17	3	14	5369.3298	-0.0079
15	4	12	16	3	13	6058.3869	-0.0078
13	4	10	14	3	11	7409.4174	0.0075

Table S21. Experimental transition frequencies (v/MHz) together with the corresponding observed - calculated differences (Δv/MHz) for the ^{13}C1 isotopologue of the FA-FA···PA complex.

J'	K_a'	K_c'	J	K_a	K_c	v/MHz	Δv/MHz
5	0	5	4	0	4	3168.5067	-0.0015
6	0	6	5	0	5	3797.3045	0.0046
7	0	7	6	0	6	4423.4712	-0.0034
8	0	8	7	0	7	5046.6790	0.0003
4	1	3	3	1	2	2616.1300	-0.0038
5	1	4	4	1	3	3269.2943	-0.0019
6	1	6	5	1	5	3696.9192	0.0037
6	1	5	5	1	4	3921.8520	-0.0073
7	1	7	6	1	6	4311.4695	0.0133
7	1	6	6	1	5	4573.6828	-0.0011
2	1	2	1	0	1	3559.5573	0.0008
3	1	3	2	0	2	4138.9508	0.0077
4	1	4	3	0	3	4700.3031	-0.0041
5	1	5	4	0	4	5244.5664	0.0031
6	1	6	5	0	5	5772.9694	-0.0011
7	1	7	6	0	6	6287.1247	-0.0022
9	1	9	8	0	8	7280.6423	-0.0044

Table S22. Experimental transition frequencies (v/MHz) together with the corresponding observed - calculated differences (Δv/MHz) for the $^{13}C2$ isotopologue of the FA-FA···PA complex.

J'	K_a'	K_c'	J	K_a	K_c	v/MHz	Δv/MHz
6	0	6	5	0	5	3832.2324	0.0017
7	0	7	6	0	6	4463.9133	0.0010
8	0	8	7	0	7	5092.4805	-0.0096
9	0	9	8	0	8	5717.6739	0.0019
0	0	10	9	0	9	6339.2676	0.0034
4	1	4	3	1	3	2488.1980	0.0038
4	1	3	3	1	2	2641.4929	0.0019
5	1	5	4	1	4	3109.3859	0.0064
5	1	4	4	1	3	3300.9437	-0.0066
6	1	6	5	1	5	3730.0072	-0.0017
6	1	5	5	1	4	3959.7861	0.0054
7	1	7	6	1	6	4349.9892	-0.0015
7	1	6	6	1	5	4617.8378	0.0020
8	1	8	7	1	7	4969.2367	-0.0065
1	1	1	0	0	0	2956.9187	-0.0038
2	1	2	1	0	1	3560.0425	0.0024
3	1	3	2	0	2	4144.1699	-0.0011
4	1	4	3	0	3	4709.9153	0.0033
5	1	5	4	0	4	5258.2184	-0.0026
6	1	6	5	0	5	5790.4231	0.0045
7	1	7	6	0	6	6308.1762	-0.0024
8	1	8	7	0	7	6813.5040	-0.0056
9	1	9	8	0	8	7308.7209	0.0054

Table S23. Experimental transition frequencies (v/MHz) together with the corresponding observed - calculated differences (Δv/MHz) for the ^{13}C3 isotopologue of the FA-FA···PA complex.

J'	K_a'	K_c'	J	K_a	K_c	v/MHz	Δv/MHz
5	0	5	4	0	4	3186.1526	0.0012
6	0	6	5	0	5	3818.4447	0.0081
7	0	7	6	0	6	4448.0888	0.0024
8	0	8	7	0	7	5074.7404	-0.0041
10	0	10	9	0	9	6318.0253	0.0022
11	0	11	10	0	10	6934.3683	0.0053
7	1	7	6	1	6	4335.4042	-0.0050
8	1	8	7	1	7	4952.6529	-0.0060
8	1	7	7	1	6	5253.7959	0.0005
4	1	3	3	1	2	2630.7453	-0.0031
5	1	5	4	1	4	3098.8766	-0.0031
5	1	4	4	1	3	3287.5496	-0.0087
6	1	6	5	1	5	3717.4620	0.0055
10	1	10	9	1	9	6184.7869	0.0012
2	1	2	1	0	1	3577.9597	0.0014
3	1	3	2	0	2	4160.5478	0.0017
4	1	4	3	0	3	4725.0053	0.0022
5	1	5	4	0	4	5272.2483	-0.0015
6	1	6	5	0	5	5803.5502	-0.0047
7	1	7	6	0	6	6320.5283	0.0005

Table S24. Experimental transition frequencies (v/MHz) together with the corresponding observed - calculated differences (Δv/MHz) for the ^{13}C4 isotopologue of the FA-FA···PA complex.

J'	K_a'	K_c'	J	K_a	K_c	v/MHz	Δv/MHz
5	0	5	4	0	4	3167.1991	0.0064
6	0	6	5	0	5	3795.7997	0.0034
7	0	7	6	0	6	4421.8299	0.0079
8	0	8	7	0	7	5044.9169	-0.0025
9	0	9	8	0	8	5664.8050	0.0018
4	1	3	3	1	2	2614.5782	-0.0087
5	1	5	4	1	4	3080.8234	-0.0036
5	1	4	4	1	3	3267.3767	0.0003
6	1	6	5	1	5	3695.8220	0.0024
6	1	5	5	1	4	3919.5680	-0.0079
7	1	7	6	1	6	4310.2016	0.0000
7	1	6	6	1	5	4571.0538	0.0046
8	1	7	7	1	6	5221.6393	-0.0103
9	1	8	8	1	7	5871.2231	0.0045
10	1	9	9	1	8	6519.5841	0.0001
6	2	5	5	2	4	3809.2609	0.0056
6	2	4	5	2	3	3824.7001	-0.0012
7	2	6	6	2	5	4443.0024	-0.0023
2	1	2	1	0	1	3571.0831	0.0036
4	1	4	3	0	3	4711.9273	0.0002
5	1	5	4	0	4	5256.3491	0.0002
6	1	6	5	0	5	5784.9733	-0.0024
7	1	7	6	0	6	6299.3836	0.0025
8	1	8	7	0	7	6801.4506	-0.0035
9	1	9	8	0	8	7293.3664	-0.0009

Table S25. Experimental transition frequencies (v/MHz) together with the corresponding observed - calculated differences (Δv/MHz) for the ^{13}C5 isotopologue of the FA-FA···PA complex.

J'	K_a'	K_c'	J	K_a	K_c	v/MHz	Δv/MHz
4	0	4	3	0	3	2518.9783	-0.0102
5	0	5	4	0	4	3145.4980	-0.0019
6	0	6	5	0	5	3769.8793	0.0008
7	0	7	6	0	6	4391.7380	-0.0002
10	0	10	9	0	9	6239.0980	0.0009
11	0	11	10	0	10	6848.1693	-0.0028
5	1	4	4	1	3	3244.3830	0.0060
6	1	6	5	1	5	3670.9929	0.0014
6	1	5	5	1	4	3892.0063	-0.0011
7	1	7	6	1	6	4281.2740	0.0021
9	1	8	8	1	7	5830.0716	0.0063
10	1	10	9	1	9	6107.8125	-0.0068
2	1	2	1	0	1	3561.3494	-0.0044
3	1	3	2	0	2	4137.0487	0.0014
4	1	4	3	0	3	4695.0067	-0.0109
5	1	5	4	0	4	5236.1470	0.0035
6	1	6	5	0	5	5761.6390	0.0039
7	1	7	6	0	6	6273.0396	0.0111
8	1	8	7	0	7	6772.1722	0.0028
9	1	9	8	0	8	7261.1683	-0.0125
10	1	10	9	0	9	7742.4180	0.0052

Table S26. Experimental transition frequencies (v/MHz) together with the corresponding observed - calculated differences (Δv/MHz) for the D1 isotopologue of the FA-FA···PA complex.

J'	K_a'	K_c'	J	K_a	K_c	v/MHz	Δv/MHz
4	0	4	3	0	3	2523.6544	0.0004
5	0	5	4	0	4	3151.1921	-0.0004
6	0	6	5	0	5	3776.5077	-0.0002
7	0	7	6	0	6	4399.1997	-0.0004
8	0	8	7	0	7	5018.9160	0.0010
9	0	9	8	0	8	5635.3639	-0.0024
10	0	10	9	0	9	6248.3681	0.0083
11	0	11	10	0	10	6857.8175	0.0029
12	0	12	11	0	11	7463.7959	0.0161
4	1	4	3	1	3	2452.4431	-0.0004
4	1	3	3	1	2	2602.0854	0.0023
5	1	5	4	1	4	3064.7256	-0.0017
5	1	4	4	1	3	3251.7293	-0.0015
6	1	6	5	1	5	3676.4798	0.0014
6	1	5	5	1	4	3900.7772	-0.0005
7	1	7	6	1	6	4287.6086	0.0006
7	1	6	6	1	5	4549.0833	-0.0009
8	1	8	7	1	7	4898.0390	0.0017
8	1	7	7	1	6	5196.5021	0.0014
9	1	9	8	1	8	5507.7058	0.0077
9	1	8	8	1	7	5842.8586	-0.0060
10	1	10	9	1	9	6116.5411	0.0073
10	1	9	9	1	8	6487.9941	-0.0056
11	1	11	10	1	10	6724.5104	0.0103
11	1	10	10	1	9	7131.6915	-0.0229
5	2	4	4	2	3	3159.2116	0.0064
5	2	3	4	2	2	3168.2162	0.0000
6	2	5	5	2	4	3790.2165	0.0025
6	2	4	5	2	3	3805.9474	0.0022
7	2	6	6	2	5	4420.7705	0.0014
7	2	5	6	2	4	4445.8456	0.0056
8	2	7	7	2	6	5050.7958	0.0001
8	2	6	7	2	5	5088.1786	0.0008
9	2	8	8	2	7	5680.2170	-0.0019
9	2	7	8	2	6	5733.1577	-0.0096
10	2	9	9	2	8	6308.9706	0.0053
10	2	8	9	2	7	6380.9006	-0.0235
11	2	10	10	2	9	6936.9685	0.0067
11	2	9	10	2	8	7031.4191	-0.0295

1	1	1	0	0	0	2938.3464	-0.0043
2	1	2	1	0	1	3532.9725	-0.0057
3	1	3	2	0	2	4109.0658	-0.0028
4	1	4	3	0	3	4667.1896	-0.0027
5	1	5	4	0	4	5208.2612	-0.0046
6	1	6	5	0	5	5733.5468	-0.0047
7	1	7	6	0	6	6244.6490	-0.0025
8	1	8	7	0	7	6743.4881	-0.0007
9	1	9	8	0	8	7232.2768	0.0048
10	1	10	9	0	9	7713.4517	0.0124
7	0	7	6	1	6	2442.1602	0.0037
8	0	8	7	1	7	3173.4673	0.0038
9	0	9	8	1	8	3910.7958	0.0033
10	0	10	9	1	9	4651.4550	0.0009
11	0	11	10	1	10	5392.7315	-0.0034
12	0	12	11	1	11	6132.0107	-0.0038
13	0	13	12	1	12	6866.8826	-0.0027
14	0	14	13	1	13	7595.2619	0.0001
12	2	10	12	1	11	5999.3935	-0.0002
11	2	9	11	1	10	6088.5810	-0.0028
10	2	8	10	1	9	6188.8477	-0.0018
8	2	6	8	1	7	6405.6155	-0.0070
7	2	5	7	1	6	6513.9334	-0.0119
6	2	4	6	1	5	6617.1761	-0.0135
5	2	3	5	1	4	6712.0136	-0.0085
4	2	2	4	1	3	6795.5272	-0.0096
3	2	1	3	1	2	6865.2872	-0.0029
3	2	2	3	1	3	7087.5248	0.0054
4	2	3	4	1	4	7162.8998	0.0057
5	2	4	5	1	5	7257.3804	0.0084
6	2	5	6	1	6	7371.1172	0.0097
7	2	6	7	1	7	7504.2855	0.0168
8	2	7	8	1	8	7657.0314	0.0043
9	2	8	9	1	9	7829.5556	0.0076
13	0	13	12	0	12	8066.4398	0.0030
11	1	11	10	0	10	8189.5922	0.0125

Table S27. Experimental transition frequencies (v/MHz) together with the corresponding observed - calculated differences (Δv/MHz) for the D2 isotopologue of the FA-FA···PA complex.

J'	K_a'	K_c'	J	K_a	K_c	v/MHz	Δv/MHz
5	0	5	4	0	4	3190.1506	-0.0065
6	0	6	5	0	5	3823.0862	-0.0050
7	0	7	6	0	6	4453.2921	-0.0192
8	0	8	7	0	7	5080.4548	0.0011
9	0	9	8	0	8	5704.2257	-0.0008
10	0	10	9	0	9	6324.4292	-0.0066
11	0	11	10	0	10	6941.0092	0.0014
4	1	3	3	1	2	2634.9596	0.0027
5	1	5	4	1	4	3102.0921	-0.0017
5	1	4	4	1	3	3292.7915	0.0001
6	1	6	5	1	5	3721.2669	-0.0091
6	1	5	5	1	4	3950.0042	0.0012
7	1	7	6	1	6	4339.8047	-0.0109
7	1	6	6	1	5	4606.4446	-0.0022
8	1	8	7	1	7	4957.6268	-0.0045
8	1	7	7	1	6	5261.9642	-0.0027
10	1	10	9	1	9	6190.8175	-0.0053
10	1	9	9	1	8	6569.5436	-0.0026
5	2	4	4	2	3	3198.4636	0.0139
5	2	3	4	2	2	3207.7837	0.0025
6	2	5	5	2	4	3837.2785	0.0011
6	2	4	5	2	3	3853.5699	0.0028
7	2	6	6	2	5	4475.6452	0.0097
7	2	5	6	2	4	4501.6024	0.0094
8	2	7	7	2	6	5113.4443	-0.0014
8	2	6	7	2	5	5152.1517	0.0086
10	2	9	9	2	8	6387.1259	0.0102
2	1	2	1	0	1	3557.1811	0.0071
3	1	3	2	0	2	4140.0264	0.0089
5	1	5	4	0	4	5251.7236	0.0006
6	1	6	5	0	5	5782.8357	-0.0062
7	1	7	6	0	6	6299.5631	-0.0031
8	1	8	7	0	7	6803.8949	0.0086
9	1	9	8	0	8	7298.0932	0.0076
9	2	7	9	1	8	6313.8529	0.0077
8	2	6	8	1	7	6424.8084	-0.0029
7	2	5	7	1	6	6534.6284	-0.0066
6	2	4	6	1	5	6639.4890	0.0002
5	2	3	5	1	4	6735.9176	-0.0072

3	2	1	3	1	2	6891.9909	0.0013

Table S28. Experimental transition frequencies (v/MHz) together with the corresponding observed - calculated differences (Δv/MHz) for the D3 isotopologue of the FA-FA···PA complex.

J'	K_a'	K_c'	*J*	K_a	K_c	*v*/MHz	Δ*v*/MHz
4	0	4	3	0	3	2560.7492	0.0006
5	0	5	4	0	4	3197.3149	0.0029
6	0	6	5	0	5	3831.4931	0.0024
7	0	7	6	0	6	4462.8510	-0.0082
8	0	8	7	0	7	5091.0472	0.0016
9	0	9	8	0	8	5715.7557	0.0007
10	0	10	9	0	9	6336.7992	0.0015
11	0	11	10	0	10	6954.1201	0.0078
12	0	12	11	0	11	7567.7891	0.0084
4	1	4	3	1	3	2487.3095	0.0006
4	1	3	3	1	2	2641.9475	0.0055
5	1	5	4	1	4	3108.2501	0.0010
5	1	4	4	1	3	3301.4850	-0.0041
6	1	6	5	1	5	3728.6206	0.0019
6	1	5	5	1	4	3960.3891	-0.0005
7	1	7	6	1	6	4348.3269	0.0033
7	1	6	6	1	5	4618.4917	-0.0009
8	1	8	7	1	7	4967.2800	-0.0004
8	1	7	7	1	6	5275.6346	-0.0012
9	1	9	8	1	8	5585.4249	0.0070
9	1	8	8	1	7	5931.6313	-0.0112
10	1	10	9	1	9	6202.6844	0.0071
10	1	9	9	1	8	6586.3114	-0.0097
11	1	11	10	1	10	6819.0301	0.0163
11	1	10	10	1	9	7239.4505	-0.0116
5	2	4	4	2	3	3205.9242	0.0066
5	2	3	4	2	2	3215.5907	-0.0051
6	2	5	5	2	4	3846.2111	0.0033
6	2	4	5	2	3	3863.1023	0.0018
7	2	6	6	2	5	4486.0131	0.0020
7	2	5	6	2	4	4512.9244	-0.0003
8	2	7	7	2	6	5125.2487	0.0015
8	2	6	7	2	5	5165.3569	-0.0012
9	2	8	8	2	7	5763.8366	0.0008
9	2	7	8	2	6	5820.6086	-0.0033
10	2	9	9	2	8	6401.7027	0.0044
10	2	8	9	2	7	6478.7854	-0.0057
11	2	10	10	2	9	7038.7580	0.0014
11	2	9	10	2	8	7139.8581	-0.0129

12	2	10	11	2	9	7803.6696	-0.0114
9	3	6	8	3	5	5781.5474	-0.0089
10	3	8	9	3	7	6423.4079	0.0041
10	3	7	9	3	6	6426.4063	0.0069
11	3	8	10	3	7	7072.1725	0.0070
1	1	1	0	0	0	2933.3099	0.0005
2	1	2	1	0	1	3536.0375	-0.0076
3	1	3	2	0	2	4119.6270	-0.0051
4	1	4	3	0	3	4684.6776	-0.0058
5	1	5	4	0	4	5232.1786	-0.0055
6	1	6	5	0	5	5763.4855	-0.0052
7	1	7	6	0	6	6280.3139	-0.0098
8	1	8	7	0	7	6784.7396	-0.0053
9	1	9	8	0	8	7279.1175	0.0003
10	1	10	9	0	9	7766.0500	0.0104
7	0	7	6	1	6	2530.8701	0.0110
8	0	8	7	1	7	3273.5833	0.0022
9	0	9	8	1	8	4022.0610	0.0054
10	0	10	9	1	9	4773.4414	0.0060
11	0	11	10	1	10	5524.8675	-0.0029
12	0	12	11	1	11	6273.6278	-0.0094
13	0	13	12	1	12	7017.2684	-0.0007
1	1	0	1	0	1	2330.5835	0.0098
2	1	1	2	0	2	2369.7109	-0.0123
3	1	2	3	0	3	2429.3658	0.0111
4	1	3	4	0	4	2510.5573	0.0091
5	1	4	5	0	5	2614.7382	0.0128
6	1	5	6	0	6	2743.6316	0.0072
7	1	6	7	0	7	2899.2745	0.0167
9	1	8	9	0	9	3299.7337	-0.0020
10	1	9	10	0	10	3549.2515	-0.0076
13	2	11	13	1	12	5873.8746	-0.0037
12	2	10	12	1	11	5944.1915	0.0133
11	2	9	11	1	10	6031.3388	0.0036
10	2	8	10	1	9	6130.9221	-0.0041
9	2	7	9	1	8	6238.4516	-0.0046
8	2	6	8	1	7	6349.4812	-0.0057
7	2	5	7	1	6	6459.7586	-0.0058
6	2	4	6	1	5	6565.3293	-0.0031
5	2	3	5	1	4	6662.6140	-0.0075
4	2	2	4	1	3	6748.5123	-0.0026
3	2	1	3	1	2	6820.3883	-0.0010
2	2	0	2	1	1	6876.1240	-0.0141

3	2	2	3	1	3	7049.9543	0.0096
4	2	3	4	1	4	7127.8705	0.0133
5	2	4	5	1	5	7225.5417	0.0161
6	2	5	6	1	6	7343.1210	0.0064
7	2	6	7	1	7	7480.8114	0.0092
8	2	7	8	1	8	7638.7633	-0.0054
9	2	8	9	1	9	7817.1733	-0.0135

Table S29. Experimental transition frequencies (v/MHz) together with the corresponding observed - calculated differences (Δv/MHz) for the D4 isotopologue of the FA-FA···PA complex.

J'	K_a'	K_c'	J	K_a	K_c	v/MHz	Δv/MHz
5	0	5	4	0	4	3183.3268	-0.0018
6	0	6	5	0	5	3814.8958	-0.0028
7	0	7	6	0	6	4443.7512	-0.0046
8	0	8	7	0	7	5069.5376	0.0008
9	0	9	8	0	8	5691.9465	-0.0036
10	0	10	9	0	9	6310.7969	-0.0058
11	0	11	10	0	10	6926.0258	0.0043
5	1	5	4	1	4	3095.3925	-0.0141
5	1	4	4	1	3	3285.8221	0.0027
6	1	6	5	1	5	3713.2463	-0.0062
6	1	5	5	1	4	3941.6419	0.0045
7	1	7	6	1	6	4330.4647	0.0083
7	1	6	6	1	5	4596.6844	-0.0037
8	1	7	7	1	6	5250.8217	0.0059
9	1	9	8	1	8	5562.6155	-0.0089
9	1	8	8	1	7	5903.8475	-0.0039
10	1	10	9	1	9	6177.4613	0.0001
10	1	9	9	1	8	6555.6130	0.0015
11	1	11	10	1	10	6791.4010	-0.0016
11	1	10	10	1	9	7205.8995	0.0034
5	2	3	4	2	2	3200.9429	-0.0071
6	2	5	5	2	4	3829.0947	0.0120
6	2	4	5	2	3	3845.3744	0.0047
7	2	5	6	2	4	4492.0479	0.0185
8	2	7	7	2	6	5102.5305	0.0080
8	2	6	7	2	5	5141.2143	0.0015
11	2	10	10	2	9	7007.8144	0.0015
11	2	9	10	2	8	7105.5027	0.0070
2	1	2	1	0	1	3547.9082	0.0099
3	1	3	2	0	2	4129.4712	0.0099
4	1	4	3	0	3	4692.7373	-0.0051
5	1	5	4	0	4	5238.6917	0.0006
6	1	6	5	0	5	5768.6081	-0.0069
8	1	8	7	0	7	6787.3463	-0.0074
9	1	9	8	0	8	7280.4409	-0.0005
10	1	10	9	0	9	7765.9558	0.0032
8	0	8	7	1	7	3229.1188	-0.0011
9	0	9	8	1	8	3974.1332	0.0001
10	0	10	9	1	9	4722.2963	-0.0151

11	2	9	11	1	10	6087.1057	0.0097
10	2	8	10	1	9	6187.5007	0.0043
9	2	7	9	1	8	6295.2275	0.0145
8	2	6	8	1	7	6405.9379	0.0041
7	2	5	7	1	6	6515.5200	-0.0168
6	2	4	6	1	5	6620.1803	-0.0153
5	2	3	5	1	4	6716.4642	0.0008
4	2	2	4	1	3	6801.3162	-0.0164
3	2	1	3	1	2	6872.2716	-0.0024
2	2	0	2	1	1	6927.2687	0.0122

Table S30. Experimental transition frequencies (v/MHz) together with the corresponding observed - calculated differences (Δv/MHz) for the D5 isotopologue of the FA-FA···PA complex.

J'	K_a'	K_c'	J	K_a	K_c	v/MHz	Δv/MHz
5	0	5	4	0	4	3198.1953	0.0017
6	0	6	5	0	5	3832.7727	0.0023
7	0	7	6	0	6	4464.6496	-0.0017
8	0	8	7	0	7	5093.4711	-0.0027
9	0	9	8	0	8	5718.9518	0.0052
10	0	10	9	0	9	6340.8675	-0.0061
11	0	11	10	0	10	6959.1721	-0.0056
4	1	4	3	1	3	2488.7794	-0.0077
4	1	3	3	1	2	2641.2990	-0.0005
5	1	5	4	1	4	3110.1324	-0.0014
5	1	4	4	1	3	3300.7323	0.0056
6	1	5	5	1	4	3959.5348	-0.0008
7	1	7	6	1	6	4351.0955	0.0015
7	1	6	6	1	5	4617.5793	-0.0035
8	1	8	7	1	7	4970.5354	-0.0002
8	1	7	7	1	6	5274.7183	0.0043
9	1	9	8	1	8	5589.1812	-0.0074
9	1	8	8	1	7	5930.7631	0.0015
10	1	10	9	1	9	6207.0080	0.0130
10	1	9	9	1	8	6585.5287	-0.0153
11	1	11	10	1	10	6823.9244	0.0144
11	1	10	10	1	9	7238.8668	0.0037
6	2	5	5	2	4	3846.8672	0.0000
6	2	4	5	2	3	3863.0413	-0.0029
7	2	6	6	2	5	4486.8241	-0.0083
7	2	5	6	2	4	4512.6164	0.0043
8	2	7	7	2	6	5126.2457	-0.0086
8	2	6	7	2	5	5164.6917	0.0024
9	2	8	8	2	7	5765.0589	0.0027
9	2	7	8	2	6	5819.4924	0.0049
10	2	9	9	2	8	6403.1596	-0.0027
10	2	8	9	2	7	6477.1240	0.0023
2	1	2	1	0	1	3574.1719	0.0108
3	1	3	2	0	2	4158.6355	0.0050
4	1	4	3	0	3	4724.7968	0.0015
5	1	5	4	0	4	5273.6021	0.0039
6	1	6	5	0	5	5806.3364	-0.0012
7	1	7	6	0	6	6324.6542	-0.0069
8	1	8	7	0	7	6830.5378	-0.0078

9	1	9	8	0	8	7326.2595	-0.0007
10	1	10	9	0	9	7814.3032	-0.0053
11	1	11	10	0	10	8297.3499	0.0051
12	1	12	11	0	11	8778.0725	0.0034
7	0	7	6	1	6	2491.0860	0.0019
8	0	8	7	1	7	3233.4805	0.0165
9	0	9	8	1	8	3981.8771	0.0022
10	0	10	9	1	9	4733.5532	-0.0067
12	0	12	11	1	11	6235.7411	-0.0075

Table S31. Experimental transition frequencies (v/MHz) together with the corresponding observed - calculated differences (Δv/MHz) for the doubly deuterated (D1 and D2) isotopologue of the FA-FA···PA complex.

J'	K_a'	K_c'	J	K_a	K_c	v/MHz	Δv/MHz
4	0	4	3	0	3	2515.7690	-0.0055
5	0	5	4	0	4	3141.2944	-0.0117
6	0	6	5	0	5	3764.5920	0.0016
7	0	7	6	0	6	4385.2205	-0.0035
9	0	9	8	0	8	5617.1807	-0.0039
10	0	10	9	0	9	6228.0285	-0.0044
11	0	11	10	0	10	6835.3224	0.0017
5	1	5	4	1	4	3054.7738	-0.0069
5	1	4	4	1	3	3242.0585	-0.0026
6	1	6	5	1	5	3664.5385	0.0079
6	1	5	5	1	4	3889.1674	0.0074
7	1	7	6	1	6	4273.6551	0.0019
7	1	6	6	1	5	4535.5147	0.0045
8	1	8	7	1	7	4882.0700	0.0007
8	1	7	7	1	6	5180.9543	-0.0054
9	1	9	8	1	8	5489.7126	0.0025
9	1	8	8	1	7	5825.3364	-0.0079
10	1	10	9	1	9	6096.5132	-0.0061
11	1	11	10	1	10	6702.4605	0.0076
6	2	5	5	2	4	3778.4552	0.0104
7	2	6	6	2	5	4407.0317	0.0047
7	2	5	6	2	4	4432.3831	0.0046
8	2	7	7	2	6	5035.0723	-0.0031
8	2	6	7	2	5	5072.8800	0.0083
1	1	1	0	0	0	2917.0941	-0.0004
2	1	2	1	0	1	3509.7169	0.0082
3	1	3	2	0	2	4083.7650	0.0052
4	1	4	3	0	3	4639.8150	-0.0098
5	1	5	4	0	4	5178.8264	-0.0047
6	1	6	5	0	5	5702.0574	0.0017
7	1	7	6	0	6	6211.1179	-0.0005
8	1	8	7	0	7	6707.9620	-0.0015
9	1	9	8	0	8	7194.8262	0.0034
10	1	10	9	0	9	7674.1540	-0.0034
4	1	3	4	0	4	2498.7370	0.0042
5	1	4	5	0	5	2599.4856	-0.0021
11	2	9	11	1	10	6032.7140	0.0015
10	2	8	10	1	9	6132.1560	-0.0039
8	2	6	8	1	7	6347.9638	0.0141

7	2	5	7	1	6	6456.0286	-0.0091
6	2	4	6	1	5	6559.1744	0.0050
5	2	3	5	1	4	6653.9810	0.0050
4	2	2	4	1	3	6737.5106	-0.0091
3	2	1	3	1	2	6807.3264	-0.0033

Table S32. Experimental transition frequencies (v/MHz) together with the corresponding observed - calculated differences (Δv/MHz) for the doubly deuterated (D1 and D3) isotopologue of the FA-FA···PA complex.

J'	K_a'	K_c'	J	K_a	K_c	v/MHz	Δv/MHz
4	0	4	3	0	3	2521.2096	-0.0014
5	0	5	4	0	4	3147.9749	0.0012
6	0	6	5	0	5	3772.4072	0.0011
7	0	7	6	0	6	4394.0926	-0.0001
8	0	8	7	0	7	5012.6715	0.0027
9	0	9	8	0	8	5627.8413	-0.0037
10	0	10	9	0	9	6239.4379	0.0041
11	0	11	10	0	10	6847.3749	0.0031
4	1	3	3	1	2	2600.9282	-0.0011
5	1	5	4	1	4	3060.4705	0.0036
5	1	4	4	1	3	3250.2402	-0.0039
6	1	6	5	1	5	3671.3122	0.0023
6	1	5	5	1	4	3898.9249	-0.0018
7	1	7	6	1	6	4281.5049	0.0013
7	1	6	6	1	5	4546.8290	-0.0007
8	1	8	7	1	7	4890.9586	-0.0078
8	1	7	7	1	6	5193.7923	-0.0024
9	1	9	8	1	8	5499.6348	0.0061
9	1	8	8	1	7	5839.6432	-0.0065
9	1	9	8	1	8	5499.6348	0.0061
9	1	8	8	1	7	5839.6432	-0.0065
10	1	10	9	1	9	6107.4348	0.0021
10	1	9	9	1	8	6484.2088	0.0015
11	1	11	10	1	10	6714.3363	0.0015
11	1	10	10	1	9	7127.2680	0.0044
12	1	12	11	1	11	7320.3052	0.0006
5	2	4	4	2	3	3156.3825	0.0033
5	2	3	4	2	2	3165.8460	0.0128
6	2	5	5	2	4	3786.7864	0.0046
6	2	4	5	2	3	3803.2828	-0.0005
7	2	6	6	2	5	4416.7089	0.0003
7	2	5	6	2	4	4442.9990	-0.0005
8	2	7	7	2	6	5046.0812	0.0005
8	2	6	7	2	5	5085.2632	-0.0026
9	2	8	8	2	7	5674.8214	0.0011
9	2	7	8	2	6	5730.2902	0.0005
10	2	9	9	2	8	6302.8541	0.0040
10	2	8	9	2	7	6378.1701	-0.0062
11	2	10	10	2	9	6930.0935	-0.0003

11	2	9	10	2	8	7028.9103	0.0056
12	2	11	11	2	10	7556.4694	-0.0079
9	3	7	8	3	6	5690.4193	-0.0022
9	3	6	8	3	5	5692.1141	-0.0085
10	3	8	9	3	7	6324.0569	0.0006
10	3	7	9	3	6	6326.9800	0.0135
11	3	9	10	3	8	6958.0020	0.0090
1	1	1	0	0	0	2894.5919	-0.0138
2	1	2	1	0	1	3488.1238	0.0023
3	1	3	2	0	2	4062.8200	-0.0103
4	1	4	3	0	3	4619.3224	-0.0090
6	1	6	5	0	5	5681.9223	-0.0012
7	1	7	6	0	6	6191.0207	-0.0002
8	1	8	7	0	7	6687.8953	0.0006
9	1	9	8	0	8	7174.8549	0.0004
10	1	10	9	0	9	7654.4484	0.0062
7	0	7	6	1	6	2484.5684	-0.0069
8	0	8	7	1	7	3215.7423	0.0016
9	0	9	8	1	8	3952.6210	0.0017
10	0	10	9	1	9	4692.4176	-0.0067
11	0	11	10	1	10	5432.3597	-0.0036
12	0	12	11	1	11	6169.7517	-0.0120
13	0	13	12	1	12	6902.2024	0.0021
14	0	14	13	1	13	7627.6055	-0.0017
1	1	0	1	0	1	2301.0792	-0.0110
2	1	1	2	0	2	2339.5395	0.0033
3	1	2	3	0	3	2398.0959	0.0049
5	1	4	5	0	5	2580.0801	0.0001
6	1	5	6	0	6	2706.6058	0.0052
7	1	6	7	0	7	2859.3257	-0.0119
8	1	7	8	0	8	3040.4640	0.0004
9	1	8	9	0	9	3252.2546	-0.0135
10	1	9	10	0	10	3497.0509	0.0092
11	1	10	11	0	11	3776.9430	0.0094
12	1	11	12	0	12	4093.7986	0.0030
15	2	13	15	1	14	5727.8726	0.0032
16	2	14	16	1	15	5731.0235	-0.0178
13	2	11	13	1	12	5802.0407	0.0003
12	2	10	12	1	11	5871.9080	-0.0090
11	2	9	11	1	10	5958.2085	0.0062
10	2	8	10	1	9	6056.5632	0.0019
9	2	7	9	1	8	6162.5963	0.0042
8	2	6	8	1	7	6271.9561	0.0040

7	2	5	7	1	6	6380.4810	0.0000
6	2	4	6	1	5	6484.3120	0.0007
5	2	3	5	1	4	6579.9526	-0.0020
4	2	2	4	1	3	6664.3646	-0.0010
3	2	1	3	1	2	6734.9714	-0.0100
2	2	1	2	1	2	6903.1753	-0.0098
3	2	2	3	1	3	6960.4288	-0.0099
4	2	3	4	1	4	7036.9646	0.0125
5	2	4	5	1	5	7132.8839	0.0194
6	2	5	6	1	6	7248.3422	0.0058

Table S33. Experimental transition frequencies (v/MHz) together with the corresponding observed - calculated differences (Δv/MHz) for the doubly deuterated (D1 and D4) isotopologue of the FA-FA···PA complex.

J'	K_a'	K_c'	J	K_a	K_c	v/MHz	Δv/MHz
5	0	5	4	0	4	3134.7604	-0.0064
6	0	6	5	0	5	3756.7272	-0.0056
7	0	7	6	0	6	4376.0378	-0.0051
8	0	8	7	0	7	4992.3390	-0.0015
9	0	9	8	0	8	5605.3373	-0.0024
10	0	10	9	0	9	6214.8355	-0.0133
4	1	3	3	1	2	2589.0831	0.0098
5	1	5	4	1	4	3048.3239	-0.0084
5	1	4	4	1	3	3235.4721	0.0155
6	1	6	5	1	5	3656.7856	-0.0031
6	1	5	5	1	4	3881.2302	0.0001
7	1	7	6	1	6	4264.6080	-0.0079
7	1	6	6	1	5	4526.2501	-0.0024
8	1	8	7	1	7	4871.7333	-0.0010
8	1	7	7	1	6	5170.3655	-0.0060
9	1	9	8	1	8	5478.0699	-0.0053
9	1	8	8	1	7	5813.4179	-0.0039
10	1	10	9	1	9	6083.5828	0.0006
10	1	9	9	1	8	6455.2284	0.0043
11	1	10	10	1	9	7095.5961	0.0131
6	2	5	5	2	4	3770.6204	0.0086
6	2	4	5	2	3	3786.5488	0.0003
7	2	6	6	2	5	4397.8838	-0.0002
7	2	5	6	2	4	4423.2728	-0.0071
8	2	7	7	2	6	5024.6232	0.0028
8	2	6	7	2	5	5062.4821	0.0003
9	2	8	8	2	7	5650.7533	0.0081
1	1	1	0	0	0	2907.8353	0.0118
2	1	2	1	0	1	3499.1782	0.0135
3	1	3	2	0	2	4071.9698	0.0113
4	1	4	3	0	3	4626.7805	-0.0022
5	1	5	4	0	4	5164.5602	-0.0059
6	1	6	5	0	5	5686.5981	0.0100
7	1	7	6	0	6	6194.4775	0.0064
8	1	8	7	0	7	6690.1612	-0.0012
11	2	9	11	1	10	6010.4566	0.0011
10	2	8	10	1	9	6109.5706	0.0128
9	2	7	9	1	8	6215.7595	0.0076
8	2	6	8	1	7	6324.8207	0.0081

7	2	5	7	1	6	6432.6894	-0.0129
6	2	4	6	1	5	6535.6619	-0.0130
5	2	3	5	1	4	6630.3494	-0.0070
4	2	2	4	1	3	6713.7931	-0.0109
3	2	1	3	1	2	6783.5428	0.0003

Table S34. Experimental transition frequencies (ν/MHz) together with the corresponding observed - calculated differences (Δν/MHz) for the doubly deuterated (D1 and D5) isotopologue of the FA-FA···PA complex.

J'	K_a'	K_c'	J	K_a	K_c	ν/MHz	$\Delta\nu$/MHz
4	0	4	3	0	3	2521.7506	-0.0156
5	0	5	4	0	4	3148.8126	-0.0048
6	0	6	5	0	5	3773.6316	-0.0036
7	0	7	6	0	6	4395.8112	-0.0070
8	0	8	7	0	7	5015.0074	-0.0039
9	0	9	8	0	8	5630.9145	-0.0137
10	0	10	9	0	9	6243.3742	-0.0008
11	0	11	10	0	10	6852.2733	0.0002
12	0	12	11	0	11	7457.6784	0.0047
4	1	3	3	1	2	2600.2899	0.0104
5	1	5	4	1	4	3062.2997	0.0039
5	1	4	4	1	3	3249.4703	-0.0018
6	1	6	5	1	5	3673.5446	-0.0103
6	1	5	5	1	4	3898.0582	-0.0027
7	1	7	6	1	6	4284.1827	-0.0074
8	1	8	7	1	7	4894.1229	0.0009
8	1	7	7	1	6	5192.8764	0.0205
9	1	9	8	1	8	5503.2833	0.0012
9	1	8	8	1	7	5838.7464	-0.0016
10	1	10	9	1	9	6111.6024	-0.0114
10	1	9	9	1	8	6483.4106	0.0057
2	1	2	1	0	1	3525.5986	0.0141
3	1	3	2	0	2	4101.1603	0.0048
4	1	4	3	0	3	4658.7574	0.0120
5	1	5	4	0	4	5199.2840	0.0093
6	1	6	5	0	5	5724.0185	0.0062
7	1	7	6	0	6	6234.5650	-0.0021
8	1	8	7	0	7	6732.8697	-0.0011
9	1	9	8	0	8	7221.1495	0.0081
10	1	10	9	0	9	7701.8277	0.0006
10	2	8	10	1	9	6169.7513	-0.0025
9	2	7	9	1	8	6276.6627	0.0031
7	2	5	7	1	6	6494.5461	0.0015
6	2	4	6	1	5	6597.7873	-0.0057
5	2	3	5	1	4	6692.6477	-0.0104
4	2	2	4	1	3	6776.2172	-0.0032

Table S35. Experimental transition frequencies (v/MHz) together with the corresponding observed - calculated differences (Δv/MHz) for the doubly deuterated (D2 and D3) isotopologue of the FA-FA···PA complex.

J'	K_a'	K_c'	J	K_a	K_c	v/MHz	Δv/MHz
5	0	5	4	0	4	3186.8730	-0.0062
6	0	6	5	0	5	3818.8944	-0.0139
7	0	7	6	0	6	4448.0939	-0.0013
8	0	8	7	0	7	5074.0608	-0.0045
9	0	9	8	0	8	5696.5212	-0.0030
10	0	10	9	0	9	6315.2842	-0.0006
11	0	11	10	0	10	6930.2901	-0.0017
4	1	4	3	1	3	2478.9201	-0.0025
4	1	3	3	1	2	2633.7975	0.0019
5	1	5	4	1	4	3097.7503	-0.0050
5	1	4	4	1	3	3291.2906	-0.0031
6	1	6	5	1	5	3715.9986	-0.0113
6	1	5	5	1	4	3948.1334	-0.0031
7	1	7	6	1	6	4333.5861	-0.0060
7	1	6	6	1	5	4604.1742	0.0031
8	1	8	7	1	7	4950.4061	-0.0114
8	1	7	7	1	6	5259.2299	-0.0022
9	1	9	8	1	8	5566.4182	0.0037
9	1	8	8	1	7	5913.1459	0.0052
10	1	10	9	1	9	6181.5226	-0.0010
10	1	9	9	1	8	6565.7094	0.0078
11	1	11	10	1	10	6795.6866	-0.0141
11	1	10	10	1	9	7216.7042	0.0021
13	1	13	12	1	12	8021.1487	-0.0023
6	2	5	5	2	4	3833.7905	-0.0020
6	2	4	5	2	3	3850.8942	0.0097
7	2	6	6	2	5	4471.5117	0.0014
7	2	5	6	2	4	4498.7333	-0.0055
8	2	6	7	2	5	5149.2351	0.0069
9	2	8	8	2	7	5745.1486	0.0087
9	2	7	8	2	6	5802.5667	0.0042
10	2	9	9	2	8	6380.9056	0.0146
10	2	8	9	2	7	6458.8304	-0.0123
11	2	10	10	2	9	7015.8282	0.0011
1	1	1	0	0	0	2911.1533	0.0158
3	1	3	2	0	2	4093.1972	0.0129
4	1	4	3	0	3	4656.0733	0.0108
5	1	5	4	0	4	5201.3656	-0.0148
6	1	6	5	0	5	5730.5227	0.0114

7	1	7	6	0	6	6245.2033	0.0081
8	1	8	7	0	7	6747.5154	-0.0021
9	1	9	8	0	8	7239.8764	0.0097
10	1	10	9	0	9	7724.8702	0.0042
7	2	5	7	1	6	6399.5391	-0.0036
6	2	4	6	1	5	6504.9676	-0.0073
5	2	3	5	1	4	6602.2196	-0.0074
4	2	2	4	1	3	6688.1442	-0.0022
3	2	1	3	1	2	6760.0756	-0.0035
2	2	0	2	1	1	6815.8991	0.0055

Table S36. Experimental transition frequencies (v/MHz) together with the corresponding observed - calculated differences (Δv/MHz) for the doubly deuterated (D3 and D4) isotopologue of the FA-FA···PA complex.

J'	K_a'	K_c'	J	K_a	K_c	v/MHz	Δv/MHz
5	0	5	4	0	4	3180.1385	-0.0002
7	0	7	6	0	6	4438.6614	-0.0102
8	0	8	7	0	7	5063.2905	-0.0146
9	0	9	8	0	8	5684.4254	-0.0059
10	0	10	9	0	9	6301.8629	-0.0009
11	0	11	10	0	10	6915.5483	0.0005
4	1	3	3	1	2	2628.2660	0.0029
5	1	5	4	1	4	3091.1742	-0.0001
5	1	4	4	1	3	3284.3762	-0.0028
6	1	6	5	1	5	3708.1118	-0.0024
6	1	5	5	1	4	3939.8382	-0.0020
7	1	7	6	1	6	4324.3739	-0.0084
7	1	6	6	1	5	4594.4912	-0.0026
8	1	7	7	1	6	5248.1700	-0.0049
9	1	9	8	1	8	5554.5791	0.0002
9	1	8	8	1	7	5900.6992	-0.0052
10	1	10	9	1	9	6168.3872	0.0104
6	2	5	5	2	4	3825.6973	0.0024
6	2	4	5	2	3	3842.7789	0.0082
7	2	6	6	2	5	4462.0591	-0.0051
7	2	5	6	2	4	4489.2643	-0.0025
8	2	7	7	2	6	5097.8682	0.0089
8	2	6	7	2	5	5138.3891	-0.0061
9	2	8	8	2	7	5733.0055	0.0065
9	2	7	8	2	6	5790.3729	0.0075
10	2	9	9	2	8	6367.4049	0.0012
2	1	2	1	0	1	3503.4329	0.0257
3	1	3	2	0	2	4083.6071	0.0149
4	1	4	3	0	3	4645.2638	0.0109
5	1	5	4	0	4	5189.3800	-0.0053
6	1	6	5	0	5	5717.3690	0.0080
7	1	7	6	0	6	6230.9109	-0.0076
8	1	8	7	0	7	6732.1438	0.0025
9	1	9	8	0	8	7223.4201	0.0052
10	1	10	9	0	9	7707.3591	-0.0012
10	2	8	10	1	9	6056.4518	-0.0055
8	2	6	8	1	7	6273.4050	-0.0012
7	2	5	7	1	6	6383.1794	-0.0065
6	2	4	6	1	5	6488.4057	-0.0073

5	2	3	5	1	4	6585.4886	0.0059
4	2	2	4	1	3	6671.2424	-0.0030

Table S37. Experimental transition frequencies (v/MHz) together with the corresponding observed - calculated differences (Δv/MHz) for the doubly deuterated (D3 and D5) isotopologue of the FA-FA···PA complex.

J'	K_a'	K_c'	J	K_a	K_c	ν/MHz	$\Delta\nu$/MHz
3	1	3	2	0	2	4111.4327	0.0040
4	1	4	3	0	3	4675.9464	0.0122
5	1	5	4	0	4	5222.8804	0.0031
7	1	7	6	0	6	6269.9054	0.0099
8	1	8	7	0	7	6773.7762	0.0047
9	1	9	8	0	8	7267.6091	-0.0139
10	1	10	9	0	9	7754.0592	-0.0008
4	0	4	3	0	3	2558.8374	-0.0160
5	0	5	4	0	4	3194.9251	0.0000
6	0	6	5	0	5	3828.5972	-0.0030
7	0	7	6	0	6	4459.4514	-0.0005
8	0	8	7	0	7	5087.1038	-0.0031
9	0	9	8	0	8	5711.2781	0.0073
10	0	10	9	0	9	6331.7493	-0.0049
11	0	11	10	0	10	6948.4950	-0.0033
12	0	12	11	0	11	7561.5959	0.0074
4	1	4	3	1	3	2485.3500	-0.0002
4	1	3	3	1	2	2640.1551	0.0077
5	1	5	4	1	4	3105.7961	-0.0003
5	1	4	4	1	3	3299.2466	0.0055
6	1	6	5	1	5	3725.6665	-0.0027
6	1	5	5	1	4	3957.6858	0.0011
7	1	7	6	1	6	4344.8758	0.0014
7	1	6	6	1	5	4615.3249	-0.0017
8	1	8	7	1	7	4963.3372	0.0092
8	1	7	7	1	6	5272.0014	-0.0018
9	1	9	8	1	8	5580.9537	-0.0049
9	1	8	8	1	7	5927.5235	-0.0137
10	1	10	9	1	9	6197.7081	0.0004
10	1	9	9	1	8	6581.7315	-0.0038
11	1	10	10	1	9	7234.3952	0.0083
10	2	8	10	1	9	6111.0957	0.0105
9	2	7	9	1	8	6218.4146	0.0022
8	2	6	8	1	7	6329.3221	-0.0034
7	2	5	7	1	6	6439.5468	-0.0065
6	2	4	6	1	5	6545.1056	-0.0162
5	2	3	5	1	4	6642.4350	-0.0100
4	2	2	4	1	3	6728.4083	0.0173

Table S38. Experimental transition frequencies (v/MHz) together with the corresponding observed - calculated differences (Δv/MHz) for the parent PA-FA···FA complex.

J'	K_a'	K_c'	J	K_a	K_c	v/MHz	Δv/MHz
3	0	3	2	0	2	1993.8924	0.0004
4	0	4	3	0	3	2655.1873	0.0124
5	0	5	4	0	4	3313.6134	-0.0011
6	0	6	5	0	5	3968.5614	0.0024
7	0	7	6	0	6	4619.4312	-0.0001
8	0	8	7	0	7	5265.7846	0.0087
9	0	9	8	0	8	5907.2937	-0.0132
10	0	10	9	0	9	6543.9677	0.0127
11	0	11	10	0	10	7175.8863	-0.0038
12	0	12	11	0	11	7803.5185	-0.0002
3	1	3	2	1	2	1928.9196	0.0009
3	1	2	2	1	1	2063.7196	0.0002
4	1	4	3	1	3	2571.0678	0.0003
4	1	3	3	1	2	2750.7680	0.0008
5	1	5	4	1	4	3212.5311	0.0000
5	1	4	4	1	3	3437.0600	0.0028
6	1	6	5	1	5	3853.1655	0.0006
6	1	5	5	1	4	4122.3712	-0.0014
7	1	7	6	1	6	4492.8413	0.0010
7	1	6	6	1	5	4806.4784	0.0003
8	1	8	7	1	7	5131.4459	-0.0029
8	1	7	7	1	6	5489.1146	-0.0011
9	1	9	8	1	8	5768.9077	0.0045
9	1	8	8	1	7	6170.0047	0.0024
10	1	10	9	1	9	6405.1425	0.0032
10	1	9	9	1	8	6848.8231	-0.0020
11	1	11	10	1	10	7040.1195	0.0029
11	1	10	10	1	9	7525.2276	-0.0136
12	1	12	11	1	11	7673.8187	0.0004
12	1	11	11	1	10	8198.8679	-0.0090
3	2	2	2	2	1	1996.7777	0.0032
3	2	1	2	2	0	1999.6481	-0.0007
4	2	3	3	2	2	2661.8090	0.0048
4	2	2	3	2	1	2668.9832	0.0012
5	2	4	4	2	3	3326.3542	0.0015
5	2	3	4	2	2	3340.6762	0.0018
6	2	5	5	2	4	3990.2992	-0.0005
6	2	4	5	2	3	4015.2608	0.0038
7	2	6	6	2	5	4653.5258	-0.0005

7	2	5	6	2	4	4693.1848	0.0005
8	2	7	7	2	6	5315.9202	0.0067
8	2	6	7	2	5	5374.7861	-0.0029
9	2	8	8	2	7	5977.3481	0.0036
9	2	7	8	2	6	6060.2287	-0.0026
10	2	9	9	2	8	6637.7075	0.0036
10	2	8	9	2	7	6749.4510	-0.0024
11	2	10	10	2	9	7296.8995	0.0196
11	2	9	10	2	8	7442.1507	-0.0015
12	2	11	11	2	10	7954.7670	0.0028
12	2	10	11	2	9	8137.7713	-0.0130
5	3	3	4	3	2	3330.3624	-0.0009
5	3	2	4	3	1	3330.5106	-0.0036
6	3	4	5	3	3	3997.2827	-0.0265
6	3	3	5	3	2	3997.7098	-0.0015
7	3	5	6	3	4	4664.6685	0.0000
7	3	4	6	3	3	4665.5671	-0.0051
8	3	6	7	3	5	5332.4626	0.0040
8	3	5	7	3	4	5334.2629	-0.0004
9	3	7	8	3	6	6000.6777	0.0015
9	3	6	8	3	5	6003.9729	-0.0042
10	3	8	9	3	7	6669.2909	-0.0036
10	3	7	9	3	6	6674.9295	-0.0051
11	3	9	10	3	8	7338.2599	-0.0031
11	3	8	10	3	7	7347.3855	-0.0020
12	3	10	11	3	9	8007.5040	-0.0039
12	3	9	11	3	8	8021.6070	-0.0107
13	3	11	12	3	10	8676.9267	-0.0056
6	4	3	5	4	2	3996.0203	-0.0067
6	4	2	5	4	1	3996.0203	-0.0067
7	4	4	6	4	3	4662.7496	-0.0009
7	4	3	6	4	2	4662.7496	-0.0009
8	4	5	7	4	4	5329.7976	-0.0086
8	4	4	7	4	3	5329.7976	-0.0086
9	4	6	8	4	5	5997.2221	0.0037
9	4	5	8	4	4	5997.2774	0.0119
10	4	7	9	4	6	6665.0645	0.0094
10	4	6	9	4	5	6665.1511	-0.0060
11	4	8	10	4	7	7333.3516	0.0060
11	4	7	10	4	6	7333.5496	0.0002
12	4	9	11	4	8	8002.1335	0.0085
12	4	8	11	4	7	8002.5164	0.0100
7	5	2	6	5	1	4661.7688	0.0039

7	5	3	6	5	2	4661.7688	0.0039
8	5	4	7	5	3	5328.3045	-0.0151
8	5	3	7	5	2	5328.3045	-0.0151
9	5	5	8	5	4	5995.1127	0.0028
9	5	4	8	5	3	5995.1127	0.0028
10	5	6	9	5	5	6662.1544	-0.0107
10	5	5	9	5	4	6662.1544	-0.0107
11	5	7	10	5	6	7329.5222	0.0072
11	5	6	10	5	5	7329.5222	0.0072
12	5	8	11	5	7	7997.1991	0.0096
12	5	7	11	5	6	7997.1991	0.0096
9	6	3	8	6	2	5994.0295	-0.0025
9	6	4	8	6	3	5994.0295	-0.0025
10	6	4	9	6	3	6660.6731	0.0042
10	6	5	9	6	4	6660.6731	0.0042
11	6	5	10	6	4	7327.5214	0.0155
11	6	6	10	6	5	7327.5214	0.0155
12	6	6	11	6	5	7994.5570	-0.0061
12	6	7	11	6	6	7994.5570	-0.0061
1	1	1	0	0	0	2749.5520	-0.0001
2	1	2	1	0	1	3370.2088	0.0024
3	1	3	2	0	2	3968.6682	0.0026
4	1	4	3	0	3	4545.8443	0.0033
5	1	5	4	0	4	5103.2009	0.0036
6	1	6	5	0	5	5642.7450	-0.0025
7	1	7	6	0	6	6167.0324	0.0035
8	1	8	7	0	7	6679.0477	0.0014
9	1	9	8	0	8	7182.1717	-0.0019
10	1	10	9	0	9	7680.0094	0.0036
6	0	6	5	1	5	2178.9765	0.0002
7	0	7	6	1	6	2945.2458	0.0030
8	0	8	7	1	7	3718.1806	0.0023
9	0	9	8	1	8	4494.0319	-0.0046
10	0	10	9	1	9	5269.0803	-0.0080
1	1	0	1	0	1	2128.9001	0.0011
2	1	1	2	0	2	2174.5574	0.0020
3	1	2	3	0	3	2244.3829	0.0002
4	1	3	4	0	4	2339.9715	-0.0035
5	1	4	5	0	5	2463.4111	-0.0065
6	1	5	6	0	6	2617.2299	-0.0013
7	1	6	7	0	7	2804.2770	-0.0010
8	1	7	8	0	8	3027.6217	0.0037
9	1	8	9	0	9	3290.3118	-0.0015

10	1	9	10	0	10	3595.1858	0.0023
11	1	10	11	0	11	3944.5276	-0.0069
12	1	11	12	0	12	4339.8924	-0.0004
13	1	12	13	0	13	4781.7841	0.0023
14	1	13	14	0	14	5269.5829	0.0042
13	2	11	13	1	12	5288.5085	-0.0070
14	2	12	14	1	13	5287.0634	-0.0075
11	2	9	11	1	10	5383.3315	-0.0027
12	2	10	12	1	11	5322.2330	-0.0085
9	2	7	9	1	8	5565.7910	-0.0038
8	2	6	8	1	7	5675.5665	0.0006
7	2	5	7	1	6	5789.8927	0.0000
5	2	3	5	1	4	6010.3020	-0.0001
4	2	2	4	1	3	6106.6965	0.0115
3	2	1	3	1	2	6188.4758	0.0056
2	2	0	2	1	1	6252.5237	-0.0169
2	2	1	2	1	2	6386.6236	-0.0067
3	2	2	3	1	3	6454.4993	0.0132
4	2	3	4	1	4	6545.2349	0.0121
5	2	4	5	1	5	6659.0407	-0.0035
6	2	5	6	1	6	6796.1829	0.0037
7	2	6	7	1	7	6956.8717	0.0065
8	2	7	8	1	8	7141.3316	0.0016
9	2	8	9	1	9	7349.7792	0.0079
10	2	9	10	1	10	7582.3306	-0.0052
11	2	10	11	1	11	7839.0963	-0.0028
4	2	2	5	1	5	3343.4653	0.0026
3	2	1	4	1	4	3887.0100	-0.0018
7	3	5	8	2	6	5091.9101	-0.0086
6	3	4	7	2	5	5802.0294	-0.0100
5	3	3	6	2	4	6497.9123	-0.0021
10	3	7	11	2	10	3343.2596	0.0030
8	3	5	9	2	8	4598.9238	-0.0045
6	3	3	7	2	6	5892.3592	0.0084
3	3	0	4	2	3	7870.4834	0.0158
2	2	1	1	1	0	7627.9412	0.0034
3	2	2	2	1	1	8248.5956	-0.0005
4	2	3	3	1	2	8846.6773	-0.0037
2	2	0	1	1	1	7673.5942	0.0013
3	2	1	2	1	2	8386.9864	-0.0116

Table S39. Experimental transition frequencies (v/MHz) together with the corresponding observed - calculated differences (Δv/MHz) for the ^{13}C1 isotopologue of the PA-FA···FA complex.

J'	K_a'	K_c'	*J*	K_a	K_c	*v*/MHz	Δ*v*/MHz
5	0	5	4	0	4	3275.6313	-0.0002
7	0	7	6	0	6	4566.9823	0.0052
8	0	8	7	0	7	5206.3131	0.0004
9	0	9	8	0	8	5840.9843	-0.0026
10	0	10	9	0	9	6470.9165	0.0011
11	0	11	10	0	10	7096.2383	-0.0033
12	0	12	11	0	11	7717.3324	-0.0039
6	1	6	5	1	5	3809.8254	-0.0024
6	1	5	5	1	4	4073.6965	0.0032
7	1	7	6	1	6	4442.3875	0.0159
7	1	6	6	1	5	4749.7936	-0.0068
8	1	8	7	1	7	5073.8818	-0.0032
8	1	7	7	1	6	5424.5044	0.0057
9	1	9	8	1	8	5704.2825	-0.0006
9	1	8	8	1	7	6097.5184	0.0015
10	1	10	9	1	9	6333.5018	-0.0004
10	1	9	9	1	8	6768.5608	0.0041
11	1	11	10	1	10	6961.4942	-0.0073
11	1	10	10	1	9	7437.2854	-0.0051
12	1	12	11	1	11	7588.2663	0.0036
8	2	7	7	2	6	5254.6105	0.0000
8	2	6	7	2	5	5311.3116	-0.0055
9	2	8	8	2	7	5908.4893	-0.0037
9	2	7	8	2	6	5988.3691	0.0033
10	2	9	9	2	8	6561.3583	0.0144
10	2	8	9	2	7	6669.0904	-0.0079

Table S40. Experimental transition frequencies (v/MHz) together with the corresponding observed - calculated differences (Δv/MHz) for the ^{13}C2 isotopologue of the PA-FA···FA complex.

J'	K_a'	K_c'	J	K_a	K_c	v/MHz	Δv/MHz
5	0	5	4	0	4	3309.4423	-0.0073
6	0	6	5	0	5	3963.4275	-0.0028
7	0	7	6	0	6	4613.2804	0.0055
8	0	8	7	0	7	5258.5260	0.0004
9	0	9	8	0	8	5898.9036	0.0021
10	0	10	9	0	9	6534.3463	0.0020
11	0	11	10	0	10	7165.0420	0.0000
4	1	3	3	1	2	2748.0271	0.0050
5	1	5	4	1	4	3208.0421	0.0016
5	1	4	4	1	3	3433.5994	-0.0016
6	1	5	5	1	4	4118.1836	-0.0042
7	1	6	6	1	5	4801.5397	-0.0023
6	1	6	5	1	5	3847.7440	-0.0010
6	1	5	5	1	4	4118.1836	-0.0042
7	1	7	6	1	6	4486.4801	0.0036
7	1	6	6	1	5	4801.5397	-0.0023
8	1	8	7	1	7	5124.1142	-0.0108
8	1	7	7	1	6	5483.4071	0.0064
9	1	9	8	1	8	5760.5938	-0.0090
9	1	8	8	1	7	6163.4749	0.0010
10	1	10	9	1	9	6395.8470	0.0006
11	1	11	10	1	10	7029.8203	0.0047
11	1	10	10	1	9	7516.9509	-0.0044
12	1	12	11	1	11	7662.4964	0.0015
6	2	5	5	2	4	3985.5448	0.0037
6	2	4	5	2	3	4010.9208	-0.0029
7	2	6	6	2	5	4647.9459	0.0027
8	2	7	7	2	6	5309.4742	-0.0174
8	2	6	7	2	5	5369.3401	-0.0057
9	2	8	8	2	7	5970.0831	0.0156
9	2	7	8	2	6	6054.3099	0.0065
10	2	8	9	2	7	6743.0743	0.0030
11	2	10	10	2	9	7287.8390	0.0022

Table S41. Experimental transition frequencies (v/MHz) together with the corresponding observed - calculated differences (Δv/MHz) for the ^{13}C3 isotopologue of the PA-FA···FA complex.

J'	K_a'	K_c'	J	K_a	K_c	v/MHz	Δv/MHz
5	0	5	4	0	4	3300.1786	0.0033
6	0	6	5	0	5	3952.5625	0.0011
8	0	8	7	0	7	5244.8616	0.0016
9	0	9	8	0	8	5884.0378	0.0046
10	0	10	9	0	9	6518.3823	-0.0003
11	0	11	10	0	10	7148.0693	0.0033
5	1	5	4	1	4	3199.8185	-0.0026
5	1	4	4	1	3	3422.5132	0.0015
6	1	5	5	1	4	4104.9618	0.0073
7	1	7	6	1	6	4475.1296	0.0066
7	1	6	6	1	5	4786.2022	-0.0065
8	1	8	7	1	7	5111.2565	0.0047
8	1	7	7	1	6	5466.0232	0.0012
9	1	9	8	1	8	5746.2299	-0.0136
9	1	8	8	1	7	6144.1182	0.0020
10	1	10	9	1	9	6380.0325	-0.0021
10	1	9	9	1	8	6820.1865	0.0010
11	1	11	10	1	10	7012.5821	-0.0017
11	1	10	10	1	9	7493.8920	-0.0020
12	1	12	11	1	11	7643.8711	-0.0028
7	2	5	6	2	4	4673.4558	-0.0072
8	2	7	7	2	6	5294.1706	-0.0018
8	2	6	7	2	5	5352.0760	0.0012
9	2	8	8	2	7	5952.9399	0.0028
9	2	7	8	2	6	6034.4757	0.0021
10	2	8	9	2	7	6720.6099	-0.0014
3	1	3	2	0	2	3962.9484	-0.0022
4	1	4	3	0	3	4538.0982	-0.0025
5	1	5	4	0	4	5093.5648	-0.0056
6	1	6	5	0	5	5631.3489	0.0102

Table S42. Experimental transition frequencies (v/MHz) together with the corresponding observed - calculated differences (Δv/MHz) for the ^{13}C4 isotopologue of the PA-FA···FA complex.

J'	K_a'	K_c'	J	K_a	K_c	v/MHz	Δv/MHz
5	0	5	4	0	4	3281.2227	0.0030
6	0	6	5	0	5	3929.9577	0.0000
7	0	7	6	0	6	4574.7628	0.0020
8	0	8	7	0	7	5215.1833	0.0023
9	0	9	8	0	8	5850.9333	0.0027
10	0	10	9	0	9	6481.9215	-0.0033
11	0	11	10	0	10	7108.3092	0.0008
12	0	12	11	0	11	7730.4531	0.0005
4	1	3	3	1	2	2722.8648	-0.0059
5	1	5	4	1	4	3181.7578	-0.0103
5	1	4	4	1	3	3402.2325	-0.0048
6	1	6	5	1	5	3816.3085	-0.0054
6	1	5	5	1	4	4080.6608	-0.0044
7	1	7	6	1	6	4449.9435	0.0099
7	1	6	6	1	5	4757.9304	0.0020
8	1	8	7	1	7	5082.5162	-0.0047
8	1	7	7	1	6	5433.7832	0.0036
9	1	9	8	1	8	5713.9903	-0.0004
9	1	8	8	1	7	6107.9490	0.0019
10	1	10	9	1	9	6344.2806	0.0015
10	1	9	9	1	8	6780.1338	0.0018
11	1	11	10	1	10	6973.3471	0.0020
11	1	10	10	1	9	7450.0060	0.0002
12	1	12	11	1	11	7601.1605	-0.0098
8	2	7	7	2	6	5263.5766	-0.0039
9	2	8	8	2	7	5918.5711	-0.0069
10	2	9	9	2	8	6572.5542	0.0125
11	2	10	10	2	9	7225.3696	0.0063
11	2	9	10	2	8	7365.8397	-0.0040
2	1	2	1	0	1	3357.8292	0.0000
3	1	3	2	0	2	3950.9121	-0.0052
4	1	4	3	0	3	4523.0870	0.0014
6	1	6	5	0	5	5610.8412	0.0006
7	1	7	6	0	6	6130.8193	0.0028

Table S43. Experimental transition frequencies (v/MHz) together with the corresponding observed - calculated differences (Δv/MHz) for the ^{13}C5 isotopologue of the PA-FA···FA complex.

J'	K_a'	K_c'	J	K_a	K_c	v/MHz	Δv/MHz
5	0	5	4	0	4	3259.1744	-0.0001
6	0	6	5	0	5	3903.6498	-0.0007
7	0	7	6	0	6	4544.2616	-0.0044
8	0	8	7	0	7	5180.5822	0.0043
9	0	9	8	0	8	5812.3064	0.0084
10	0	10	9	0	9	6439.3371	0.0020
11	0	11	10	0	10	7061.8212	0.0003
5	1	5	4	1	4	3160.7009	-0.0128
5	1	4	4	1	3	3378.7626	-0.0117
6	1	6	5	1	5	3791.0916	0.0078
7	1	7	6	1	6	4420.5433	-0.0015
7	1	6	6	1	5	4725.1879	0.0016
8	1	8	7	1	7	5048.9928	0.0002
8	1	7	7	1	6	5396.4424	0.0025
9	1	9	8	1	8	5676.3436	0.0009
9	1	8	8	1	7	6066.0464	0.0008
10	1	10	9	1	9	6302.5163	-0.0151
10	1	9	9	1	8	6733.7097	-0.0021
11	1	11	10	1	10	6927.5237	0.0054
11	1	10	10	1	9	7399.1217	0.0039
12	1	12	11	1	11	7551.2854	0.0014
7	2	5	6	2	4	4614.0315	-0.0017
8	2	7	7	2	6	5228.0256	-0.0126
8	2	6	7	2	5	5283.7600	0.0025
9	2	8	8	2	7	5878.6551	0.0039
9	2	7	8	2	6	5957.1529	0.0015
10	2	9	9	2	8	6528.2522	0.0014
11	2	10	10	2	9	7176.7332	0.0028
12	2	11	11	2	10	7823.9858	-0.0014
3	1	3	2	0	2	3934.9783	-0.0007
4	1	4	3	0	3	4503.6165	-0.0040
5	1	5	4	0	4	5052.9478	0.0030
6	1	6	5	0	5	5584.8565	0.0026
7	1	7	6	0	6	6101.7468	-0.0013

Table S44. Experimental transition frequencies (v/MHz) together with the corresponding observed - calculated differences (Δv/MHz) for the D1 isotopologue of the PA-FA···FA complex.

J'	K_a'	K_c'	J	K_a	K_c	v/MHz	Δv/MHz
4	0	4	3	0	3	2608.6160	0.0041
5	0	5	4	0	4	3255.6530	0.0020
6	0	6	5	0	5	3899.3511	0.0022
7	0	7	6	0	6	4539.1496	-0.0004
8	0	8	7	0	7	5174.6103	-0.0001
9	0	9	8	0	8	5805.4429	-0.0013
10	0	10	9	0	9	6431.5637	-0.0028
11	0	11	10	0	10	7053.1156	-0.0032
12	0	12	11	0	11	7670.4606	-0.0070
3	1	2	2	1	1	2026.7972	0.0018
4	1	4	3	1	3	2526.6147	-0.0023
4	1	3	3	1	2	2701.5729	-0.0013
5	1	5	4	1	4	3157.0287	0.0024
5	1	4	4	1	3	3375.6297	-0.0006
6	1	6	5	1	5	3786.6443	0.0024
6	1	5	5	1	4	4048.7579	0.0008
7	1	7	6	1	6	4415.3422	0.0017
7	1	6	6	1	5	4720.7297	-0.0012
8	1	8	7	1	7	5043.0185	0.0014
8	1	7	7	1	6	5391.3054	-0.0016
9	1	9	8	1	8	5669.5865	-0.0005
9	1	8	8	1	7	6060.2069	-0.0097
10	1	10	9	1	9	6294.9847	-0.0024
10	1	9	9	1	8	6727.1611	-0.0028
11	1	11	10	1	10	6919.1720	-0.0049
11	1	10	10	1	9	7391.8188	-0.0052
12	1	12	11	1	11	7542.1295	-0.0084
6	2	5	5	2	4	3920.1198	0.0038
6	2	4	5	2	3	3943.9556	0.0006
7	2	6	6	2	5	4571.7301	0.0024
7	2	5	6	2	4	4609.6172	-0.0010
8	2	7	7	2	6	5222.5381	0.0001
8	2	6	7	2	5	5278.8106	-0.0002
9	2	8	8	2	7	5872.4325	-0.0025
9	2	7	8	2	6	5951.6980	-0.0010
10	2	9	9	2	8	6521.3095	0.0006
10	2	8	9	2	7	6628.2418	-0.0041
11	2	10	10	2	9	7169.0522	0.0000
11	2	9	10	2	8	7308.1797	-0.0045

12	2	11	11	2	10	7815.5575	-0.0036
12	2	10	11	2	9	7991.0077	-0.0088
9	3	7	8	3	6	5894.7536	0.0106
9	3	6	8	3	5	5897.8368	0.0016
10	3	8	9	3	7	6551.5282	0.0049
10	3	7	9	3	6	6556.8117	0.0041
11	3	9	10	3	8	7208.6509	0.0037
11	3	8	10	3	7	7217.1921	-0.0051
12	3	10	11	3	9	7866.0499	0.0036
12	3	9	11	3	8	7879.2707	-0.0002
2	1	2	1	0	1	3333.0980	0.0027
3	1	3	2	0	2	3921.5976	-0.0021
4	1	4	3	0	3	4489.3598	-0.0002
5	1	5	4	0	4	5037.7752	0.0005
6	1	6	5	0	5	5568.7659	0.0003
7	1	7	6	0	6	6084.7542	-0.0029
8	1	8	7	0	7	6588.6289	0.0046
9	1	9	8	0	8	7083.6126	0.0117
10	1	10	9	0	9	7573.1461	0.0022
7	0	7	6	1	6	2869.7260	-0.0073
8	0	8	7	1	7	3628.9992	-0.0041
9	0	9	8	1	8	4391.4350	0.0045
10	0	10	9	1	9	5153.4181	0.0081
11	0	11	10	1	10	5911.5268	-0.0147
12	0	12	11	1	11	6662.8477	0.0154
13	0	13	12	1	12	7404.8832	0.0199
13	2	11	13	1	12	5252.4781	-0.0040
10	2	8	10	1	9	5435.7401	0.0026
8	2	6	8	1	7	5643.1789	0.0057
7	2	5	7	1	6	5755.6657	-0.0036
6	2	4	6	1	5	5866.7794	-0.0025
5	2	3	5	1	4	5971.5816	-0.0025
4	2	2	4	1	3	6065.7215	0.0011
4	2	3	4	1	4	6492.8804	-0.0033
6	2	5	6	1	6	6737.1523	0.0047
9	2	8	9	1	9	7275.9046	0.0010

Table S45. Experimental transition frequencies (v/MHz) together with the corresponding observed - calculated differences (Δv/MHz) for the D2 isotopologue of the PA-FA···FA complex.

J'	K_a'	K_c'	*J*	K_a	K_c	*v*/MHz	Δ*v*/MHz
4	0	4	3	0	3	2645.4681	0.0031
6	0	6	5	0	5	3953.9404	0.0048
7	0	7	6	0	6	4602.3295	0.0004
8	0	8	7	0	7	5246.1721	-0.0077
9	0	9	8	0	8	5885.2032	-0.0020
10	0	10	9	0	9	6519.3199	-0.0199
11	0	11	10	0	10	7148.7614	0.0004
12	0	12	11	0	11	7773.8952	0.0124
4	1	3	3	1	2	2740.9824	0.0045
5	1	4	4	1	3	3424.8151	-0.0003
6	1	6	5	1	5	3838.7781	0.0009
6	1	5	5	1	4	4107.6837	0.0090
7	1	7	6	1	6	4476.0453	0.0013
7	1	6	6	1	5	4789.3204	0.0015
8	1	8	7	1	7	5112.2387	-0.0010
8	1	7	7	1	6	5469.4871	-0.0016
9	1	9	8	1	8	5747.2775	0.0002
9	1	8	8	1	7	6147.8960	-0.0031
10	1	10	9	1	9	6381.0988	0.0063
10	1	9	9	1	8	6824.2247	-0.0108
11	1	11	10	1	10	7013.6478	0.0027
11	1	10	10	1	9	7498.1667	0.0142
12	1	12	11	1	11	7644.9275	0.0087
5	2	3	4	2	2	3328.6245	-0.0041
6	2	5	5	2	4	3975.7685	0.0021
6	2	4	5	2	3	4000.8459	0.0163
7	2	6	6	2	5	4636.5650	0.0013
7	2	5	6	2	4	4676.3912	0.0035
8	2	7	7	2	6	5296.5164	-0.0019
8	2	6	7	2	5	5355.6383	0.0033
9	2	8	8	2	7	5955.5110	-0.0020
9	2	7	8	2	6	6038.7240	-0.0046
10	2	9	9	2	8	6613.4327	0.0008
10	2	8	9	2	7	6725.5941	-0.0109
11	2	10	10	2	9	7270.1650	0.0023
11	2	9	10	2	8	7415.9415	-0.0129
12	2	11	11	2	10	7925.5877	-0.0089
11	3	9	10	3	8	7311.7052	0.0049
11	3	8	10	3	7	7320.9186	0.0044

2	1	2	1	0	1	3351.5488	0.0018
3	1	3	2	0	2	3947.6798	0.0145
4	1	4	3	0	3	4522.5275	-0.0032
5	1	5	4	0	4	5077.6174	0.0035
6	1	6	5	0	5	5614.9310	-0.0050
7	1	7	6	0	6	6137.0318	-0.0126
9	1	9	8	0	8	7148.0526	0.0000

Table S46. Experimental transition frequencies (v/MHz) together with the corresponding observed - calculated differences (Δv/MHz) for the D3 isotopologue of the PA-FA···FA complex.

J'	K_a'	K_c'	J	K_a	K_c	v/MHz	Δv/MHz
4	0	4	3	0	3	2645.2346	0.0028
5	0	5	4	0	4	3301.0927	0.0007
6	0	6	5	0	5	3953.3983	0.0006
7	0	7	6	0	6	4601.5657	-0.0004
8	0	8	7	0	7	5245.1420	0.0013
9	0	9	8	0	8	5883.8421	0.0003
10	0	10	9	0	9	6517.6124	-0.0005
11	0	11	10	0	10	7146.6452	0.0000
12	0	12	11	0	11	7771.3620	-0.0069
3	1	2	2	1	1	2056.5520	-0.0030
4	1	4	3	1	3	2560.9544	0.0020
4	1	3	3	1	2	2741.1986	-0.0006
5	1	5	4	1	4	3199.8637	-0.0012
5	1	4	4	1	3	3425.0711	-0.0006
6	1	6	5	1	5	3837.9354	0.0014
6	1	5	5	1	4	4107.9540	0.0027
7	1	7	6	1	6	4475.0294	-0.0001
7	1	6	6	1	5	4789.5988	0.0014
8	1	8	7	1	7	5111.0417	-0.0005
8	1	7	7	1	6	5469.7460	-0.0006
9	1	9	8	1	8	5745.8838	-0.0007
9	1	8	8	1	7	6148.1096	0.0007
10	1	10	9	1	9	6379.4907	-0.0018
10	1	9	9	1	8	6824.3641	-0.0004
11	1	11	10	1	10	7011.8235	-0.0033
11	1	10	10	1	9	7498.1666	0.0045
12	1	12	11	1	11	7642.8691	-0.0030
5	2	4	4	2	3	3314.0647	0.0109
5	2	3	4	2	2	3328.6245	-0.0039
6	2	5	5	2	4	3975.5212	0.0034
6	2	4	5	2	3	4000.9137	0.0011
7	2	6	6	2	5	4636.2509	0.0024
7	2	5	6	2	4	4676.5921	-0.0018
8	2	7	7	2	6	5296.1255	0.0003
8	2	6	7	2	5	5356.0021	-0.0016
9	2	8	8	2	7	5955.0309	0.0020
9	2	7	8	2	6	6039.2932	-0.0007
10	2	9	9	2	8	6612.8436	0.0009
10	2	8	9	2	7	6726.3898	-0.0008

11	2	10	10	2	9	7269.4545	0.0019
11	2	9	10	2	8	7416.9672	-0.0007
12	2	11	11	2	10	7924.7528	0.0037
9	3	7	8	3	6	5978.7459	-0.0031
9	3	6	8	3	5	5982.1547	-0.0027
10	3	8	9	3	7	6644.9644	0.0128
10	3	7	9	3	6	6650.7758	0.0011
11	3	9	10	3	8	7311.5096	0.0060
11	3	8	10	3	7	7320.9184	-0.0042
12	3	10	11	3	9	7978.3244	-0.0034
2	1	2	1	0	1	3340.9362	-0.0029
3	1	3	2	0	2	3936.7375	-0.0058
4	1	4	3	0	3	4511.2169	0.0004
5	1	5	4	0	4	5065.8498	0.0000
6	1	6	5	0	5	5602.6887	-0.0031
7	1	7	6	0	6	6124.3264	0.0025
8	1	8	7	0	7	6633.8066	0.0065
9	1	9	8	0	8	7134.5362	-0.0076
10	1	10	9	0	9	7630.1963	0.0017
6	0	6	5	1	5	2188.6305	-0.0092
7	0	7	6	1	6	2952.2717	-0.0002
8	0	8	7	1	7	3722.3921	0.0091
10	0	10	9	1	9	5266.9063	-0.0046
11	0	11	10	1	10	6034.0528	-0.0108
12	0	12	11	1	11	6793.6071	0.0013
13	0	13	12	1	12	7543.1488	0.0066
2	2	1	1	1	0	7550.4921	-0.0094
2	2	0	1	1	1	7596.3005	-0.0061
12	2	10	12	1	11	5256.8751	-0.0045
9	2	7	9	1	8	5494.7115	0.0002
8	2	6	8	1	7	5603.5302	0.0040
7	2	5	7	1	6	5717.2614	-0.0075
6	2	4	6	1	5	5830.2705	-0.0017
5	2	3	5	1	4	5937.3147	0.0038
4	2	2	4	1	3	6033.7667	0.0125
5	2	4	5	1	5	6587.6638	0.0042
7	2	6	7	1	7	6886.4684	0.0063
8	2	7	8	1	8	7071.5497	0.0047
9	2	8	9	1	9	7280.6897	0.0003
10	2	9	10	1	10	7514.0367	-0.0027
11	2	10	11	1	11	7771.6677	0.0025
12	2	11	12	1	12	8053.5352	-0.0069

Table S47. Experimental transition frequencies (v/MHz) together with the corresponding observed - calculated differences (Δv/MHz) for the D4 isotopologue of the PA-FA···FA complex.

J'	K_a'	K_c'	J	K_a	K_c	v/MHz	Δv/MHz
4	0	4	3	0	3	2650.0943	-0.0075
5	0	5	4	0	4	3307.0278	-0.0055
6	0	6	5	0	5	3960.3227	0.0052
7	0	7	6	0	6	4609.3598	-0.0006
8	0	8	7	0	7	5253.7012	-0.0004
9	0	9	8	0	8	5893.0636	-0.0037
10	0	10	9	0	9	6527.4141	-0.0026
11	0	11	10	0	10	7156.9706	0.0038
12	0	12	11	0	11	7782.1809	0.0020
4	1	4	3	1	3	2565.1065	0.0083
4	1	3	3	1	2	2747.1186	0.0080
5	1	5	4	1	4	3204.9993	-0.0122
5	1	4	4	1	3	3432.4351	0.0127
6	1	6	5	1	5	3844.0534	-0.0056
6	1	5	5	1	4	4116.7088	-0.0047
7	1	7	6	1	6	4482.1040	-0.0043
7	1	6	6	1	5	4799.7392	0.0029
8	1	8	7	1	7	5119.0534	0.0056
8	1	7	7	1	6	5481.2201	0.0014
9	1	9	8	1	8	5754.7850	-0.0039
9	1	8	8	1	7	6160.8623	0.0015
10	1	10	9	1	9	6389.2676	-0.0002
10	1	9	9	1	8	6838.3299	-0.0018
11	1	11	10	1	10	7022.4492	0.0033
11	1	10	10	1	9	7513.2618	-0.0062
12	1	12	11	1	11	7654.3157	0.0066
6	2	5	5	2	4	3983.0347	0.0014
6	2	4	5	2	3	4009.1255	0.0152
7	2	6	6	2	5	4644.9699	0.0033
7	2	5	6	2	4	4686.3957	0.0090
8	2	7	7	2	6	5306.0195	-0.0035
8	2	6	7	2	5	5367.4718	-0.0042
9	2	8	8	2	7	5966.0858	0.0053
9	2	7	8	2	6	6052.5180	-0.0030
10	2	9	9	2	8	6625.0129	-0.0056
10	2	8	9	2	7	6741.4231	-0.0046
11	2	10	10	2	9	7282.7189	-0.0020
11	2	9	10	2	8	7433.8293	-0.0116
12	2	11	11	2	10	7939.0849	0.0098

3	1	3	2	0	2	3924.1360	0.0047
4	1	4	3	0	3	4499.0408	0.0119
5	1	5	4	0	4	5053.9352	-0.0033
6	1	6	5	0	5	5590.9501	-0.0142

Table S48. Experimental transition frequencies (v/MHz) together with the corresponding observed - calculated differences (Δv/MHz) for the D5 isotopologue of the PA-FA···FA complex.

J'	K_a'	K_c'	J	K_a	K_c	v/MHz	Δv/MHz
4	0	4	3	0	3	2655.1822	0.0037
5	0	5	4	0	4	3313.6173	-0.0015
6	0	6	5	0	5	3968.5634	-0.0003
7	0	7	6	0	6	4619.4390	0.0025
8	0	8	7	0	7	5265.7797	-0.0014
9	0	9	8	0	8	5907.3124	0.0000
10	0	10	9	0	9	6543.9594	-0.0008
11	0	11	10	0	10	7175.8953	0.0000
12	0	12	11	0	11	7803.5243	0.0005
3	1	2	2	1	1	2063.7214	-0.0011
4	1	4	3	1	3	2571.0710	0.0004
4	1	3	3	1	2	2750.7684	-0.0029
5	1	5	4	1	4	3212.5349	0.0000
5	1	4	4	1	3	3437.0620	-0.0001
6	1	6	5	1	5	3853.1698	0.0005
6	1	5	5	1	4	4122.3754	-0.0026
7	1	7	6	1	6	4492.8445	-0.0006
7	1	6	6	1	5	4806.4856	0.0017
8	1	8	7	1	7	5131.4536	-0.0004
8	1	7	7	1	6	5489.1201	-0.0014
9	1	9	8	1	8	5768.9092	0.0005
9	1	8	8	1	7	6170.0054	-0.0022
10	1	10	9	1	9	6405.1454	0.0004
10	1	9	9	1	8	6848.8322	0.0022
11	1	11	10	1	10	7040.1223	0.0000
11	1	10	10	1	9	7525.2444	-0.0007
12	1	12	11	1	11	7673.8193	-0.0047
5	2	4	4	2	3	3326.3665	0.0095
5	2	3	4	2	2	3340.6731	-0.0057
6	2	5	5	2	4	3990.3058	0.0010
6	2	4	5	2	3	4015.2608	-0.0012
7	2	6	6	2	5	4653.5322	0.0006
7	2	5	6	2	4	4693.1885	-0.0013
8	2	7	7	2	6	5315.9179	-0.0011
8	2	6	7	2	5	5374.7927	-0.0020
9	2	8	8	2	7	5977.3512	0.0013
10	2	9	9	2	8	6637.7104	0.0013
10	2	8	9	2	7	6749.4555	-0.0030
11	2	10	10	2	9	7296.8863	0.0017

11	2	9	10	2	8	7442.1529	-0.0037
12	2	11	11	2	10	7954.7691	0.0009
9	3	7	8	3	6	6000.6757	-0.0059
9	3	6	8	3	5	6003.9779	-0.0046
10	3	8	9	3	7	6669.3040	0.0045
10	3	7	9	3	6	6674.9335	-0.0062
11	3	9	10	3	8	7338.2739	0.0063
11	3	8	10	3	7	7347.3946	0.0025
2	1	2	1	0	1	3370.2043	-0.0035
3	1	3	2	0	2	3968.6683	0.0009
4	1	4	3	0	3	4545.8458	0.0026
5	1	5	4	0	4	5103.2010	0.0012
6	1	6	5	0	5	5642.7514	0.0012
7	1	7	6	0	6	6167.0335	0.0018
8	1	8	7	0	7	6679.0498	0.0005
9	1	9	8	0	8	7182.1820	0.0051
10	1	10	9	0	9	7680.0071	-0.0022
6	0	6	5	1	5	2178.9939	0.0111
8	0	8	7	1	7	3718.1848	-0.0010
10	0	10	9	1	9	5269.0948	-0.0010
11	0	11	10	1	10	6039.8394	-0.0067
12	0	12	11	1	11	6803.2477	0.0001
13	0	13	12	1	12	7556.8761	0.0067
14	2	12	14	1	13	5287.0702	-0.0009
13	2	11	13	1	12	5288.5161	-0.0001
12	2	10	12	1	11	5322.2436	0.0013
11	2	9	11	1	10	5383.3339	-0.0005
10	2	8	10	1	9	5466.4234	0.0004
9	2	7	9	1	8	5565.7919	-0.0024
8	2	6	8	1	7	5675.5630	-0.0021
7	2	5	7	1	6	5789.8944	0.0025
6	2	4	6	1	5	5903.1895	0.0036
5	2	3	5	1	4	6010.3070	0.0051
3	2	2	3	1	3	6454.4913	0.0029
4	2	3	4	1	4	6545.2229	-0.0026
5	2	4	5	1	5	6659.0387	-0.0087
6	2	5	6	1	6	6796.1803	-0.0026
7	2	6	7	1	7	6956.8696	0.0003
8	2	7	8	1	8	7141.3377	0.0034
1	1	0	1	0	1	2128.8945	-0.0045
2	1	1	2	0	2	2174.5500	-0.0057
3	1	2	3	0	3	2244.3906	0.0070
4	1	3	4	0	4	2339.9741	-0.0023

6	1	5	6	0	6	2617.2302	-0.0038
7	1	6	7	0	7	2804.2850	0.0035
8	1	7	8	0	8	3027.6170	-0.0048
9	1	8	9	0	9	3290.3297	0.0125
11	1	10	11	0	11	3944.5388	0.0019
12	1	11	12	0	12	4339.8928	0.0001
13	1	12	13	0	13	4781.7754	-0.0027

Table S49. Experimental transition frequencies (v/MHz) together with the corresponding observed - calculated differences (Δv/MHz) for the doubly deuterated (D1 and D2) isotopologue of the PA-FA···FA complex.

J'	K_a'	K_c'	J	K_a	K_c	v/MHz	Δv/MHz
4	0	4	3	0	3	2599.4856	0.0043
6	0	6	5	0	5	3885.5881	-0.0084
7	0	7	6	0	6	4523.0610	-0.0042
8	0	8	7	0	7	5156.1775	-0.0009
9	0	9	8	0	8	5784.6503	-0.0021
10	0	10	9	0	9	6408.4026	-0.0038
11	0	11	10	0	10	7027.5938	0.0058
12	0	12	11	0	11	7642.5772	0.0065
5	1	5	4	1	4	3145.7515	-0.0024
6	1	6	5	1	5	3773.1014	-0.0053
6	1	5	5	1	4	4034.9337	0.0001
7	1	7	6	1	6	4399.5319	-0.0064
7	1	6	6	1	5	4704.5980	0.0060
8	1	8	7	1	7	5024.9381	-0.0057
8	1	7	7	1	6	5372.8415	-0.0048
9	1	9	8	1	8	5649.2323	-0.0056
9	1	8	8	1	7	6039.4171	-0.0090
10	1	10	9	1	9	6272.3558	-0.0019
10	1	9	9	1	8	6704.0231	-0.0103
11	1	11	10	1	10	6894.2652	0.0023
12	1	12	11	1	11	7514.9336	-0.0014
5	2	3	4	2	2	3270.1595	0.0032
6	2	5	5	2	4	3906.4435	0.0077
6	2	4	5	2	3	3930.3825	0.0076
7	2	6	6	2	5	4555.7749	0.0146
8	2	7	7	2	6	5204.2866	0.0068
8	2	6	7	2	5	5260.7851	0.0033
9	2	8	8	2	7	5851.8926	0.0104
9	2	7	8	2	6	5931.4598	0.0004
10	2	8	9	2	7	6605.7967	-0.0027

Table S50. Experimental transition frequencies (v/MHz) together with the corresponding observed - calculated differences (Δv/MHz) for the doubly deuterated (D1 and D3) isotopologue of the PA-FA···FA complex.

J'	K_a'	K_c'	J	K_a	K_c	v/MHz	Δv/MHz
4	0	4	3	0	3	2599.2387	0.0001
5	0	5	4	0	4	3243.8405	0.0014
6	0	6	5	0	5	3885.0355	-0.0023
7	0	7	6	0	6	4522.2698	-0.0028
8	0	8	7	0	7	5155.0940	-0.0037
9	0	9	8	0	8	5783.2291	-0.0030
10	0	10	9	0	9	6406.6019	-0.0012
11	0	11	10	0	10	7025.3732	0.0013
12	0	12	11	0	11	7639.9357	0.0074
4	1	4	3	1	3	2517.0421	-0.0026
4	1	3	3	1	2	2692.6111	0.0050
5	1	5	4	1	4	3145.0298	-0.0073
5	1	4	4	1	3	3364.3993	0.0044
6	1	6	5	1	5	3772.2146	-0.0064
6	1	5	5	1	4	4035.2404	0.0042
7	1	7	6	1	6	4398.4642	-0.0074
7	1	6	6	1	5	4704.9035	0.0022
8	1	8	7	1	7	5023.6783	-0.0041
8	1	7	7	1	6	5373.1395	-0.0004
9	1	9	8	1	8	5647.7648	-0.0035
9	1	8	8	1	7	6039.6754	-0.0012
10	1	10	9	1	9	6270.6677	0.0015
10	1	9	9	1	8	6704.2016	-0.0061
11	1	11	10	1	10	6892.3387	0.0029
11	1	10	10	1	9	7366.3907	-0.0095
12	1	12	11	1	11	7512.7694	0.0091
5	2	4	4	2	3	3256.2368	0.0047
5	2	3	4	2	2	3270.1595	-0.0064
6	2	5	5	2	4	3906.1918	0.0008
6	2	4	5	2	3	3930.4713	-0.0019
7	2	6	6	2	5	4555.4505	0.0017
7	2	5	6	2	4	4594.0450	0.0079
8	2	7	7	2	6	5203.8911	0.0012
9	2	8	8	2	7	5851.3948	-0.0055
9	2	7	8	2	6	5932.0707	0.0000
10	2	9	9	2	8	6497.8639	-0.0041
10	2	8	9	2	7	6606.6454	-0.0021
11	2	10	10	2	9	7143.1824	-0.0014
11	2	9	10	2	8	7284.6208	-0.0035

12	2	11	11	2	10	7787.2384	-0.0039
12	2	10	11	2	9	7965.4660	-0.0111
10	3	8	9	3	7	6528.6205	0.0052
10	3	7	9	3	6	6534.0816	0.0011
11	3	9	10	3	8	7183.4666	0.0000
11	3	8	10	3	7	7192.3123	0.0038
12	3	10	11	3	9	7838.5919	0.0025
12	3	9	11	3	8	7852.2766	0.0131
2	1	2	1	0	1	3303.8430	0.0271
3	1	3	2	0	2	3889.7928	0.0053
4	1	4	3	0	3	4454.9734	0.0129
5	1	5	4	0	4	5000.7664	0.0074
6	1	6	5	0	5	5529.1402	-0.0007
7	1	7	6	0	6	6042.5786	0.0038
9	1	9	8	0	8	7036.6491	-0.0061
8	2	6	8	1	7	5570.1498	-0.0045
7	2	5	7	1	6	5682.1013	-0.0098
6	2	4	6	1	5	5792.9675	-0.0077
5	2	3	5	1	4	5897.7377	-0.0004
4	2	2	4	1	3	5991.9725	0.0054

Table S51. Experimental transition frequencies (v/MHz) together with the corresponding observed - calculated differences (Δv/MHz) for the doubly deuterated (D1 and D4) isotopologue of the PA-FA···FA complex.

J'	K_a'	K_c'	J	K_a	K_c	v/MHz	Δv/MHz
4	0	4	3	0	3	2603.6141	0.0024
5	0	5	4	0	4	3249.1635	-0.0081
6	0	6	5	0	5	3891.2391	-0.0060
7	0	7	6	0	6	4529.2571	-0.0025
8	0	8	7	0	7	5162.7601	-0.0056
9	0	9	8	0	8	5791.4838	-0.0032
10	0	10	9	0	9	6415.3664	0.0007
11	0	11	10	0	10	7034.5886	0.0031
12	0	12	11	0	11	7649.5715	0.0059
4	1	3	3	1	2	2697.9422	0.0019
5	1	5	4	1	4	3149.6155	-0.0105
5	1	4	4	1	3	3371.0307	0.0038
6	1	6	5	1	5	3777.6745	-0.0084
6	1	5	5	1	4	4043.1487	0.0081
7	1	7	6	1	6	4404.7755	-0.0090
7	1	6	6	1	5	4714.0463	0.0003
8	1	8	7	1	7	5030.8216	-0.0013
8	1	7	7	1	6	5383.4873	0.0027
9	1	9	8	1	8	5655.7068	-0.0052
9	1	8	8	1	7	6051.1682	-0.0041
10	1	10	9	1	9	6279.3888	-0.0001
10	1	9	9	1	8	6716.7873	-0.0087
11	1	11	10	1	10	6901.8159	0.0017
11	1	10	10	1	9	7379.9973	-0.0136
12	1	12	11	1	11	7522.9731	0.0005
7	2	6	6	2	5	4563.2674	0.0055
7	2	5	6	2	4	4602.8454	0.0100
8	2	7	7	2	6	5212.7686	0.0086
8	2	6	7	2	5	5271.5072	0.0102
9	2	8	8	2	7	5861.3164	0.0124
9	2	7	8	2	6	5943.9754	0.0053
10	2	9	9	2	8	6508.7825	0.0033
10	2	8	9	2	7	6620.1803	-0.0047
11	2	10	10	2	9	7155.0904	0.0165
11	2	9	10	2	8	7299.8160	-0.0095
12	2	11	11	2	10	7800.0746	-0.0054

Table S52. Experimental transition frequencies (v/MHz) together with the corresponding observed - calculated differences (Δv/MHz) for the parent PA-FA···PA complex.

J'	K_a'	K_c'	J	K_a	K_c	v/MHz	Δv/MHz
5	0	5	4	0	4	2161.0197	-0.0019
6	0	6	5	0	5	2590.6247	-0.0017
7	0	7	6	0	6	3018.8374	0.0008
8	0	8	7	0	7	3445.4509	0.0004
9	0	9	8	0	8	3870.2934	0.0005
10	0	10	9	0	9	4293.2209	-0.0039
11	0	11	10	0	10	4714.1545	0.0006
12	0	12	11	0	11	5133.0437	-0.0004
13	0	13	12	0	12	5549.9221	0.0000
14	0	14	13	0	13	5964.8824	0.0038
15	0	15	14	0	14	6378.0642	0.0000
16	0	16	15	0	15	6789.6910	0.0115
17	0	17	16	0	16	7199.9607	0.0015
18	0	18	17	0	17	7609.1587	0.0035
5	1	4	4	1	3	2223.6761	0.0002
6	1	6	5	1	5	2526.3122	0.0011
6	1	5	5	1	4	2667.7360	0.0048
7	1	7	6	1	6	2946.4897	-0.0139
7	1	6	6	1	5	3111.4005	-0.0014
8	1	8	7	1	7	3366.3174	-0.0025
8	1	7	7	1	6	3554.6286	0.0161
9	1	9	8	1	8	3785.7196	-0.0009
9	1	8	8	1	7	3997.2827	0.0013
10	1	10	9	1	9	4204.6677	-0.0034
10	1	9	9	1	8	4439.3207	-0.0008
11	1	11	10	1	10	4623.1514	0.0082
11	1	10	10	1	9	4880.6374	-0.0013
12	1	12	11	1	11	5041.1142	0.0000
12	1	11	11	1	10	5321.1306	-0.0003
13	1	12	12	1	11	5760.7009	0.0123
14	1	14	13	1	13	5875.5047	0.0105
14	1	13	13	1	12	6199.1888	-0.0047
15	1	15	14	1	14	6291.8886	-0.0005
15	1	14	14	1	13	6636.5186	-0.0015
16	1	16	15	1	15	6707.7654	0.0105
16	1	15	15	1	14	7072.5414	0.0064
17	1	17	16	1	16	7123.0888	-0.0100
17	1	16	16	1	15	7507.0982	-0.0007
18	1	18	17	1	17	7537.9290	-0.0048

18	1	17	17	1	16	7940.0652	-0.0044
19	1	19	18	1	18	7952.2773	0.0002
5	2	4	4	2	3	2165.2503	-0.0001
5	2	3	4	2	2	2169.9997	0.0019
6	2	5	5	2	4	2597.8659	0.0035
6	2	4	5	2	3	2606.1539	-0.0020
7	2	6	6	2	5	3030.2284	-0.0070
7	2	5	6	2	4	3043.4689	0.0004
8	2	7	7	2	6	3462.3307	0.0006
8	2	6	7	2	5	3482.0897	-0.0064
9	2	8	8	2	7	3894.1107	0.0038
9	2	7	8	2	6	3922.1744	0.0006
10	2	9	9	2	8	4325.5305	0.0038
10	2	8	9	2	7	4363.8010	0.0016
11	2	10	10	2	9	4756.5473	-0.0033
11	2	9	10	2	8	4807.0227	-0.0009
12	2	10	11	2	9	5251.8354	-0.0051
13	2	12	12	2	11	5617.2626	0.0029
13	2	11	12	2	10	5698.1773	-0.0028
14	2	13	13	2	12	6046.8688	-0.0017
14	2	12	13	2	11	6145.9149	0.0077
15	2	14	14	2	13	6475.9336	-0.0043
15	2	13	14	2	12	6594.8157	-0.0091
16	2	15	15	2	14	6904.4275	-0.0005
16	2	14	15	2	13	7044.6807	-0.0039
17	2	16	16	2	15	7332.3170	0.0086
17	2	15	16	2	14	7495.1999	-0.0009
18	2	17	17	2	16	7759.5423	-0.0063
18	2	16	17	2	15	7946.0582	-0.0103
7	3	5	6	3	4	3033.9859	0.0020
7	3	4	6	3	3	3034.1706	-0.0018
8	3	6	7	3	5	3467.9232	0.0026
8	3	5	7	3	4	3468.3008	0.0033
9	3	7	8	3	6	3902.0338	0.0018
9	3	6	8	3	5	3902.7225	0.0001
10	3	8	9	3	7	4336.3206	-0.0019
10	3	7	9	3	6	4337.5016	-0.0027
11	3	9	10	3	8	4770.7912	0.0008
11	3	8	10	3	7	4772.6989	-0.0085
12	3	10	11	3	9	5205.4228	-0.0051
12	3	9	11	3	8	5208.3983	-0.0049
13	3	11	12	3	10	5640.2160	-0.0053
13	3	10	12	3	9	5644.6720	0.0008

14	3	12	13	3	11	6075.1630	0.0122
14	3	11	13	3	10	6081.5907	-0.0080
15	3	12	14	3	11	6519.2918	0.0102
16	3	14	15	3	13	6945.3079	0.0014
16	3	13	15	3	12	6957.8318	0.0098
17	3	15	16	3	14	7380.4616	-0.0012
17	3	14	16	3	13	7397.3365	0.0081
18	3	16	17	3	15	7815.6103	-0.0063
15	3	13	14	3	12	6510.1834	-0.0064
13	4	10	12	4	9	5637.3101	-0.0024
13	4	9	12	4	8	5637.3926	-0.0091
16	4	13	15	4	12	6941.7458	0.0090
16	4	12	15	4	11	6942.1340	0.0058
18	4	15	17	4	14	7812.4493	0.0017
18	4	14	17	4	13	7813.3476	0.0007
1	1	1	0	0	0	2176.0613	-0.0033
2	1	2	1	0	1	2585.6348	-0.0009
3	1	3	2	0	2	2983.5022	-0.0024
4	1	4	3	0	3	3369.9769	0.0057
5	1	5	4	0	4	3745.5165	-0.0007
6	1	6	5	0	5	4110.8159	0.0093
7	1	7	6	0	6	4466.6779	-0.0058
8	1	8	7	0	7	4814.1581	-0.0090
9	1	9	8	0	8	5154.4337	-0.0036
10	1	10	9	0	9	5488.8102	-0.0054
11	1	11	10	0	10	5818.7315	-0.0025
12	1	12	11	0	11	6145.7009	0.0065
13	1	13	12	0	12	6471.2196	0.0014
14	1	14	13	0	13	6796.7967	0.0066
15	1	15	14	0	14	7123.7917	-0.0089
16	1	16	15	0	15	7453.4861	-0.0052
17	1	17	16	0	16	7786.9164	0.0056
2	2	0	1	1	1	6142.3717	0.0084
3	2	1	2	1	2	6600.0602	0.0028
4	2	2	3	1	3	7070.9407	0.0083
6	2	4	5	1	5	8056.3218	0.0012
3	2	2	2	1	1	6528.1112	-0.0044
4	2	3	3	1	2	6925.8598	-0.0034
5	2	4	4	1	3	7311.8089	0.0021
6	2	5	5	1	4	7685.9910	-0.0022
10	2	9	10	1	10	5922.3919	0.0042
9	2	8	9	1	9	5801.5334	0.0012
8	2	7	8	1	8	5693.1463	0.0004

7	2	6	7	1	7	5597.1375	0.0015
6	2	5	6	1	6	5513.4079	0.0038
5	2	4	5	1	5	5441.8362	-0.0165
4	2	3	4	1	4	5382.3836	-0.0053
3	2	2	3	1	3	5334.9279	-0.0006
2	2	1	2	1	2	5299.4005	0.0006
2	2	0	2	1	1	5228.8874	0.0030
3	2	1	3	1	2	5194.6232	0.0107
5	2	3	5	1	4	5096.4420	-0.0005
6	2	4	6	1	5	5034.8714	0.0040
7	2	5	7	1	6	4966.9360	0.0022
8	2	6	8	1	7	4894.4205	0.0029
9	2	7	9	1	8	4819.3113	0.0013
11	2	9	11	1	10	4670.1745	0.0017
12	2	10	12	1	11	4600.8905	0.0081
11	2	10	11	1	11	6055.7694	-0.0256
7	2	6	6	1	5	8048.5006	0.0031
2	2	1	1	1	0	6118.5405	-0.0006
11	2	10	11	1	11	6055.7694	-0.0256
5	2	3	4	1	4	7555.9483	-0.0027
9	0	9	8	1	8	2501.5793	0.0032
10	0	10	9	1	9	3009.0748	-0.0055
11	0	11	10	1	10	3518.5594	-0.0036
12	0	12	11	1	11	4028.4594	-0.0046
13	0	13	12	1	12	4537.2737	0.0017
14	0	14	13	1	13	5043.5861	0.0034
15	0	15	14	1	14	5546.1502	-0.0025
17	0	17	16	1	16	6536.1475	0.0002
18	0	18	17	1	17	7022.2038	0.0002
19	0	19	18	1	18	7501.7863	-0.0015
20	0	20	19	1	19	7974.7903	-0.0032
18	2	16	18	1	17	4409.6369	-0.0059
16	2	14	16	1	15	4415.5491	0.0070
15	2	13	15	1	14	4443.3807	-0.0117
10	2	8	10	1	9	4743.7731	-0.0146
14	2	12	14	1	13	4485.0856	-0.0020
13	2	11	13	1	12	4538.3748	0.0008
4	2	2	4	1	3	5150.1223	0.0015
12	2	11	12	1	12	6201.8344	0.0127
13	2	12	13	1	13	6360.5217	0.0082
14	2	13	14	1	14	6531.9066	0.0167
15	2	14	15	1	15	6715.9425	0.0039
19	2	18	19	1	19	7577.2782	-0.0018

Table S53. Experimental transition frequencies (v/MHz) together with the corresponding observed - calculated differences (Δv/MHz) for the D1 isotopologue of the PA-FA···PA complex.

J'	K_a'	K_c'	J	K_a	K_c	v/MHz	Δv/MHz
5	0	5	4	0	4	2158.6088	-0.0015
6	0	6	5	0	5	2587.7253	-0.0029
7	0	7	6	0	6	3015.4472	-0.0018
8	0	8	7	0	7	3441.5694	-0.0015
9	0	9	8	0	8	3865.9201	0.0016
10	0	10	9	0	9	4288.3495	-0.0030
11	0	11	10	0	10	4708.7751	-0.0062
12	0	12	11	0	11	5127.1652	-0.0038
13	0	13	12	0	12	5543.5389	-0.0038
14	0	14	13	0	13	5957.9929	-0.0013
15	0	15	14	0	14	6370.6889	0.0138
16	0	16	15	0	15	6781.7948	0.0082
17	0	17	16	0	16	7191.5527	-0.0114
18	0	18	17	0	17	7600.2545	-0.0059
6	1	6	5	1	5	2523.4339	-0.0069
7	1	7	6	1	6	2943.1433	-0.0097
8	1	8	7	1	7	3362.4856	-0.0026
9	1	9	8	1	8	3781.4036	-0.0030
9	1	8	8	1	7	3992.9238	0.0033
10	1	10	9	1	9	4199.8676	-0.0062
10	1	9	9	1	8	4434.4762	0.0033
11	1	11	10	1	10	4617.8530	-0.0085
11	1	10	10	1	9	4875.3027	0.0017
12	1	12	11	1	11	5035.3477	0.0005
12	1	11	11	1	10	5315.3022	-0.0003
13	1	13	12	1	12	5452.3207	0.0065
13	1	12	12	1	11	5754.3673	-0.0002
14	1	14	13	1	13	5868.7522	-0.0003
14	1	13	13	1	12	6192.3744	-0.0032
15	1	15	14	1	14	6284.6603	0.0017
15	1	14	14	1	13	6629.2007	-0.0058
16	1	16	15	1	15	6700.0472	0.0132
16	1	15	15	1	14	7064.7054	-0.0152
17	1	17	16	1	16	7114.9029	0.0164
17	1	16	16	1	15	7498.7713	-0.0091
7	2	6	6	2	5	3026.8709	0.0019
7	2	5	6	2	4	3040.1236	-0.0017
8	2	7	7	2	6	3458.4833	0.0020

8	2	6	7	2	5	3478.2755	-0.0064
9	2	8	8	2	7	3889.7928	0.0176
9	2	7	8	2	6	3917.8886	-0.0022
10	2	9	9	2	8	4320.7073	-0.0040
11	2	10	10	2	9	4751.2490	-0.0020
11	2	9	10	2	8	4801.8084	-0.0011
12	2	11	11	2	10	5181.3557	-0.0003
12	2	10	11	2	9	5246.1714	0.0083
13	2	12	12	2	11	5610.9864	-0.0023
13	2	11	12	2	10	5692.0383	-0.0015
14	2	13	13	2	12	6040.1057	-0.0066
14	2	12	13	2	11	6139.3083	0.0047
15	2	14	14	2	13	6468.6866	-0.0048
15	2	13	14	2	12	6587.7520	-0.0044
16	2	14	15	2	13	7037.1406	-0.0080
11	3	8	10	3	7	4767.4442	0.0030
12	3	10	11	3	9	5199.6774	0.0014
12	3	9	11	3	8	5202.6622	-0.0004
14	3	12	13	3	11	6068.4467	0.0045
14	3	11	13	3	10	6074.9192	0.0044
16	3	14	15	3	13	6937.6355	-0.0046
16	3	13	15	3	12	6950.2197	0.0158
4	1	4	3	0	3	3364.1389	0.0103
5	1	5	4	0	4	3739.2282	0.0175
6	1	6	5	0	5	4104.0505	0.0094
7	1	7	6	0	6	4459.4514	-0.0144
10	1	10	9	0	9	5480.2942	-0.0019
11	1	11	10	0	10	5809.7979	-0.0073
12	1	12	11	0	11	6136.3587	-0.0123
13	1	13	12	0	12	6461.5117	-0.0045
14	1	14	13	0	13	6786.7279	0.0018
15	1	15	14	0	14	7113.4113	0.0209
10	0	10	9	1	9	3007.9258	-0.0044
11	0	11	10	1	10	3516.8436	0.0058
13	0	13	12	1	12	4534.3566	0.0159
15	0	15	14	1	14	5541.9357	-0.0074
16	0	16	15	1	15	6039.0664	-0.0047
10	2	8	10	1	9	4732.3906	-0.0089
9	2	7	9	1	8	4807.8153	-0.0070
8	2	6	8	1	7	4882.8427	-0.0093
7	2	5	7	1	6	4955.3077	-0.0004
6	2	4	6	1	5	5023.1996	0.0031
4	2	2	4	1	3	5138.3895	-0.0032

3	2	1	3	1	2	5182.8701	0.0022
2	2	1	2	1	2	5287.6236	-0.0027
2	2	0	2	1	1	5217.1293	0.0003
2	2	0	1	1	1	6129.6418	0.0068
5	2	4	4	1	3	7297.6820	0.0126
6	2	5	5	1	4	7671.3924	0.0025

Table S54. Experimental transition frequencies (v/MHz) together with the corresponding observed - calculated differences (Δv/MHz) for the D2 isotopologue of the PA-FA···PA complex.

J'	K_a'	K_c'	J	K_a	K_c	v/MHz	Δv/MHz
5	0	5	4	0	4	2157.8776	-0.0018
6	0	6	5	0	5	2586.7601	-0.0001
7	0	7	6	0	6	3014.1963	0.0000
8	0	8	7	0	7	3439.9825	0.0019
9	0	9	8	0	8	3863.9360	0.0013
10	0	10	9	0	9	4285.9212	0.0021
11	0	11	10	0	10	4705.8443	-0.0004
12	0	12	11	0	11	5123.6849	0.0018
13	0	13	12	0	12	5539.4719	0.0004
14	0	14	13	0	13	5953.3146	0.0000
15	0	15	14	0	14	6365.3766	-0.0020
16	0	16	15	0	15	6775.8789	-0.0006
17	0	17	16	0	16	7185.0632	-0.0026
18	0	18	17	0	17	7593.1967	-0.0032
5	1	5	4	1	4	2102.1459	0.0005
5	1	4	4	1	3	2221.3039	-0.0035
6	1	6	5	1	5	2521.9156	-0.0032
6	1	5	5	1	4	2664.8635	0.0002
7	1	7	6	1	6	2941.3481	-0.0006
7	1	6	6	1	5	3108.0189	-0.0005
8	1	8	7	1	7	3360.3888	-0.0009
8	1	7	7	1	6	3550.7004	0.0033
9	1	9	8	1	8	3779.0019	0.0006
9	1	8	8	1	7	3992.8101	-0.0013
10	1	10	9	1	9	4197.1493	0.0009
10	1	9	9	1	8	4434.2706	-0.0006
11	1	11	10	1	10	4614.8040	0.0017
11	1	10	10	1	9	4874.9757	-0.0019
12	1	12	11	1	11	5031.9445	0.0039
12	1	11	11	1	10	5314.8246	0.0004
13	1	13	12	1	12	5448.5497	0.0025
13	1	12	12	1	11	5753.6974	0.0018
14	1	14	13	1	13	5864.6137	0.0007
14	1	13	13	1	12	6191.4673	-0.0009
15	1	15	14	1	14	6280.1356	0.0007
15	1	14	14	1	13	6628.0054	-0.0048
16	1	16	15	1	15	6695.1160	0.0000
16	1	15	15	1	14	7063.1835	0.0013
17	1	17	16	1	16	7109.5678	0.0021

17	1	16	16	1	15	7496.8387	0.0000
18	1	18	17	1	17	7523.4929	-0.0049
18	1	17	17	1	16	7928.8308	-0.0011
19	1	19	18	1	18	7936.9208	-0.0104
5	2	3	4	2	2	2167.1719	-0.0159
6	2	5	5	2	4	2594.2662	0.0031
6	2	4	5	2	3	2602.8618	-0.0017
7	2	6	6	2	5	3026.0150	0.0011
7	2	5	6	2	4	3039.7311	-0.0034
8	2	7	7	2	6	3457.4774	0.0012
8	2	6	7	2	5	3477.9638	-0.0021
9	2	8	8	2	7	3888.6084	-0.0003
9	2	7	8	2	6	3917.6936	-0.0007
10	2	9	9	2	8	4319.3710	-0.0003
10	2	8	9	2	7	4359.0163	-0.0001
11	2	10	10	2	9	4749.7238	0.0000
11	2	9	10	2	8	4801.9773	-0.0007
12	2	11	11	2	10	5179.6288	0.0023
12	2	10	11	2	9	5246.5630	-0.0011
13	2	12	12	2	11	5609.0421	0.0013
13	2	11	12	2	10	5692.6927	0.0005
14	2	13	13	2	12	6037.9306	0.0016
14	2	12	13	2	11	6140.2106	-0.0004
15	2	14	14	2	13	6466.2542	-0.0001
15	2	13	14	2	12	6588.9079	0.0015
16	2	15	15	2	14	6893.9799	-0.0024
16	2	14	15	2	13	7038.5100	-0.0019
17	2	16	16	2	15	7321.0809	0.0014
17	2	15	16	2	14	7488.7231	-0.0032
18	2	17	17	2	16	7747.5223	0.0073
18	2	16	17	2	15	7939.2309	0.0001
9	3	7	8	3	6	3896.8232	0.0049
9	3	6	8	3	5	3897.5645	0.0117
10	3	8	9	3	7	4330.5506	-0.0010
10	3	7	9	3	6	4331.8022	-0.0068
11	3	9	10	3	8	4764.4662	0.0001
11	3	8	10	3	7	4766.5073	0.0016
12	3	10	11	3	9	5198.5606	0.0078
12	3	9	11	3	8	5201.7210	0.0031
13	3	11	12	3	10	5632.8014	0.0048
13	3	10	12	3	9	5637.5271	-0.0020
14	3	12	13	3	11	6067.1779	0.0025
14	3	11	13	3	10	6074.0128	-0.0192

15	3	13	14	3	12	6501.6614	0.0001
15	3	12	14	3	11	6511.3292	0.0026
16	3	14	15	3	13	6936.2228	0.0032
16	3	13	15	3	12	6949.5233	0.0028
17	3	15	16	3	14	7370.8119	0.0016
17	3	14	16	3	13	7388.7274	0.0003
18	3	16	17	3	15	7805.3891	0.0009
18	3	15	17	3	14	7829.0638	0.0016
2	1	2	1	0	1	2557.9461	-0.0067
3	1	3	2	0	2	2954.8427	-0.0066
5	1	5	4	0	4	3714.5924	-0.0041
6	1	6	5	0	5	4078.6340	-0.0019
7	1	7	6	0	6	4433.2223	-0.0021
8	1	8	7	0	7	4779.4164	-0.0015
9	1	9	8	0	8	5118.4377	-0.0008
10	1	10	9	0	9	5451.6498	-0.0023
11	1	11	10	0	10	5780.5362	0.0008
12	1	12	11	0	11	6106.6351	0.0040
13	1	13	12	0	12	6431.5104	0.0152
14	1	14	13	0	13	6756.6390	0.0023
15	1	15	14	0	14	7083.4573	0.0003
16	1	16	15	0	15	7413.1979	0.0035
17	1	17	16	0	16	7746.8766	-0.0039
8	0	8	7	1	7	2020.9456	-0.0068
9	0	9	8	1	8	2524.4956	-0.0017
10	0	10	9	1	9	3031.4194	0.0041
11	0	11	10	1	10	3540.1058	-0.0059
12	0	12	11	1	11	4048.9937	0.0011
13	0	13	12	1	12	4556.5249	0.0013
14	0	14	13	1	13	5061.2851	-0.0057
15	0	15	14	1	14	5562.0624	0.0059
16	0	16	15	1	15	6057.7996	-0.0015
17	0	17	16	1	16	6547.7560	0.0051
4	2	2	3	1	3	6991.1258	0.0062
5	2	3	4	1	4	7476.2328	0.0040
6	2	4	5	1	5	7976.9517	0.0048
3	2	2	2	1	1	6447.6303	0.0127
4	2	3	3	1	2	6844.3798	-0.0082
5	2	4	4	1	3	7229.2203	-0.0062
6	2	5	5	1	4	7602.1746	-0.0076
7	2	6	6	1	5	7963.3305	-0.0024
8	2	7	7	1	6	8312.7801	-0.0095
17	2	15	17	1	16	4337.7416	-0.0033

16	2	14	16	1	15	4345.8600	0.0026
18	2	16	18	1	17	4348.1423	-0.0015
15	2	13	15	1	14	4370.5334	0.0059
14	2	12	14	1	13	4409.6253	-0.0059
13	2	11	13	1	12	4460.8886	0.0001
12	2	10	12	1	11	4521.8922	0.0001
11	2	9	11	1	10	4590.1506	-0.0013
10	2	8	10	1	9	4663.1484	-0.0032
9	2	7	9	1	8	4738.4045	-0.0020
8	2	6	8	1	7	4813.5238	0.0001
7	2	5	7	1	6	4886.2530	-0.0017
6	2	4	6	1	5	4954.5400	0.0003
5	2	3	5	1	4	5016.5413	0.0019
4	2	2	4	1	3	5070.6621	0.0032
4	2	3	4	1	4	5305.3493	0.0053
5	2	4	5	1	5	5365.4678	0.0046
6	2	5	6	1	6	5437.8112	0.0037
7	2	6	7	1	7	5522.4733	0.0006
8	2	7	8	1	8	5619.5565	-0.0024
9	2	8	9	1	9	5729.1635	-0.0030
10	2	9	10	1	10	5851.3950	0.0054
11	2	10	11	1	11	5986.3077	-0.0033
12	2	11	12	1	12	6133.9969	-0.0002

Table S55. Experimental transition frequencies (v/MHz) together with the corresponding observed - calculated differences (Δv/MHz) for the D3 isotopologue of the PA-FA···PA complex.

J'	K_a'	K_c'	J	K_a	K_c	v/MHz	Δv/MHz
5	0	5	4	0	4	2155.9563	0.0008
6	0	6	5	0	5	2584.4587	0.0061
7	0	7	6	0	6	3011.5040	-0.0012
8	0	8	7	0	7	3436.9052	-0.0014
9	0	9	8	0	8	3860.4736	-0.0048
10	0	10	9	0	9	4282.0772	-0.0039
11	0	11	10	0	10	4701.6215	-0.0041
13	0	13	12	0	12	5534.4840	-0.0077
14	0	14	13	0	13	5947.9523	-0.0030
15	0	15	14	0	14	6359.6341	-0.0065
16	0	16	15	0	15	6769.7608	-0.0024
17	0	17	16	0	16	7178.5852	0.0132
18	0	18	17	0	17	7586.3392	0.0104
5	1	4	4	1	3	2219.3324	-0.0063
6	1	6	5	1	5	2519.6642	0.0038
6	1	5	5	1	4	2662.5017	0.0000
7	1	7	6	1	6	2938.7063	-0.0075
7	1	6	6	1	5	3105.2742	0.0086
8	1	8	7	1	7	3357.3750	-0.0033
8	1	7	7	1	6	3547.5551	0.0037
9	1	9	8	1	8	3775.6089	-0.0043
9	1	8	8	1	7	3989.2755	0.0009
10	1	10	9	1	9	4193.3772	-0.0064
10	1	9	9	1	8	4430.3470	0.0034
11	1	11	10	1	10	4610.6536	-0.0071
11	1	10	10	1	9	4870.6653	0.0053
12	1	12	11	1	11	5027.4193	-0.0027
12	1	11	11	1	10	5310.1196	0.0025
13	1	13	12	1	12	5443.6497	-0.0018
13	1	12	12	1	11	5748.6067	0.0069
14	1	14	13	1	13	5859.3403	0.0003
14	1	13	13	1	12	6185.9780	-0.0061
15	1	15	14	1	14	6274.4887	0.0047
15	1	14	14	1	13	6622.1257	-0.0127
16	1	16	15	1	15	6689.0820	-0.0050
16	1	15	15	1	14	7056.9112	-0.0118
7	2	6	6	2	5	3023.3235	0.0054
7	2	5	6	2	4	3037.0369	0.0021
8	2	7	7	2	6	3454.3947	-0.0011

8	2	6	7	2	5	3474.8853	0.0052
9	2	8	8	2	7	3885.1462	0.0018
9	2	7	8	2	6	3914.2303	0.0082
10	2	8	9	2	7	4355.1592	0.0013
11	2	10	10	2	9	4745.4992	0.0075
11	2	9	10	2	8	4797.7376	0.0046
12	2	11	11	2	10	5175.0118	0.0008
12	2	10	11	2	9	5241.9349	0.0024
13	2	12	12	2	11	5604.0405	-0.0015
13	2	11	12	2	10	5687.6758	0.0020
14	2	13	13	2	12	6032.5446	-0.0027
14	2	12	13	2	11	6134.8037	-0.0021
15	2	14	14	2	13	6460.4897	-0.0003
15	2	13	14	2	12	6583.1101	-0.0042
16	2	15	15	2	14	6887.8228	-0.0125
16	2	14	15	2	13	7032.3183	-0.0146
17	2	16	16	2	15	7314.5626	0.0124
17	2	15	16	2	14	7482.1626	0.0024
12	3	10	11	3	9	5193.9434	0.0139
12	3	9	11	3	8	5197.1052	0.0099
13	3	10	12	3	9	5632.5276	0.0047
14	3	12	13	3	11	6061.7825	-0.0015
14	3	11	13	3	10	6068.6428	0.0003
15	3	13	14	3	12	6495.8798	-0.0064
15	3	12	14	3	11	6505.5518	-0.0026
16	3	13	15	3	12	6943.3706	0.0040
17	3	15	16	3	14	7364.2582	-0.0107
17	3	14	16	3	13	7382.1960	0.0038
2	1	2	1	0	1	2555.2040	0.0159
3	1	3	2	0	2	2951.7415	0.0159
4	1	4	3	0	3	3336.7752	0.0187
5	1	5	4	0	4	3710.7938	0.0137
6	1	6	5	0	5	4074.4927	0.0078
7	1	7	6	0	6	4428.7496	0.0033
8	1	8	7	0	7	4774.6120	-0.0073
9	1	9	8	0	8	5113.3148	-0.0111
10	1	10	9	0	9	5446.2277	-0.0034
11	1	11	10	0	10	5774.7961	-0.0147
12	1	12	11	0	11	6100.5978	-0.0095
13	1	13	12	0	12	6425.1688	-0.0067
14	1	14	13	0	13	6750.0235	-0.0001
15	1	15	14	0	14	7076.5544	0.0020
9	2	7	9	1	8	4732.7674	-0.0095

8	2	6	8	1	7	4807.8150	-0.0143
7	2	5	7	1	6	4880.4953	-0.0054
6	2	4	6	1	5	4948.7352	0.0037
5	2	3	5	1	4	5010.6919	0.0085

Table S56. Experimental transition frequencies (v/MHz) together with the corresponding observed - calculated differences (Δv/MHz) for the D4 isotopologue of the PA-FA···PA complex.

J'	K_a'	K_c'	J	K_a	K_c	v/MHz	Δv/MHz
5	0	5	4	0	4	2158.2009	0.0024
6	0	6	5	0	5	2587.2342	0.0023
7	0	7	6	0	6	3014.8636	-0.0031
8	0	8	7	0	7	3440.9094	0.0077
9	0	9	8	0	8	3865.1632	0.0020
10	0	10	9	0	9	4287.5085	0.0022
11	0	11	10	0	10	4707.8390	-0.0060
12	0	12	11	0	11	5126.1415	-0.0010
13	0	13	12	0	12	5542.4275	0.0014
14	0	14	13	0	13	5956.7866	-0.0015
15	0	15	14	0	14	6369.3828	0.0020
16	0	16	15	0	15	6780.4104	0.0047
18	0	18	17	0	17	7598.7151	0.0014
6	1	6	5	1	5	2522.9302	-0.0049
6	1	5	5	1	4	2664.3514	-0.0007
7	1	7	6	1	6	2942.5652	0.0022
7	1	6	6	1	5	3107.4603	0.0032
8	1	8	7	1	7	3361.8161	0.0021
8	1	7	7	1	6	3550.1010	0.0008
9	1	9	8	1	8	3780.6511	0.0028
9	1	8	8	1	7	3992.2025	0.0023
10	1	10	9	1	9	4199.0343	0.0025
10	1	9	9	1	8	4433.6647	-0.0047
11	1	11	10	1	10	4616.9353	-0.0006
11	1	10	10	1	9	4874.4182	0.0049
12	1	12	11	1	11	5034.3261	-0.0121
12	1	11	11	1	10	5314.3246	-0.0050
13	1	13	12	1	12	5451.2205	-0.0020
13	1	12	12	1	11	5753.3039	-0.0043
14	1	14	13	1	13	5867.5736	-0.0053
14	1	13	13	1	12	6191.2284	-0.0023
15	1	15	14	1	14	6283.3985	-0.0050
16	1	16	15	1	15	6698.7016	0.0029
16	1	15	15	1	14	7063.3956	0.0010
17	1	16	16	1	15	7497.3625	-0.0003
7	2	6	6	2	5	3026.3059	0.0101
7	2	5	6	2	4	3039.5642	-0.0001
8	2	7	7	2	6	3457.8320	0.0064
8	2	6	7	2	5	3477.6426	-0.0016

9	2	8	8	2	7	3889.0388	0.0022
9	2	7	8	2	6	3917.1637	-0.0136
10	2	9	9	2	8	4319.8879	-0.0020
10	2	8	9	2	7	4358.2686	0.0071
11	2	10	10	2	9	4750.3463	-0.0001
11	2	9	10	2	8	4800.9443	-0.0023
12	2	11	11	2	10	5180.3679	-0.0002
12	2	10	11	2	9	5245.2232	-0.0027
13	2	12	12	2	11	5609.9226	0.0053
13	2	11	12	2	10	5691.0268	-0.0015
14	2	13	13	2	12	6038.9568	-0.0003
14	2	12	13	2	11	6138.2141	-0.0027
15	2	14	14	2	13	6467.4496	-0.0028
15	2	13	14	2	12	6586.5936	0.0003
16	2	15	15	2	14	6895.3670	-0.0021
16	2	14	15	2	13	7035.9081	0.0007
17	2	16	16	2	15	7322.6847	0.0098
17	2	15	16	2	14	7485.8677	-0.0044
12	3	10	11	3	9	5198.6953	-0.0041
13	3	11	12	3	10	5632.9228	-0.0110
13	3	10	12	3	9	5637.4048	-0.0027
14	3	12	13	3	11	6067.3070	0.0030
14	3	11	13	3	10	6073.7999	0.0135
15	3	13	14	3	12	6501.7951	0.0118
16	3	14	15	3	13	6936.3451	0.0054
16	3	13	15	3	12	6948.9125	-0.0087
17	3	15	16	3	14	7370.9362	0.0010
2	1	2	1	0	1	2579.6797	-0.0053
3	1	3	2	0	2	2976.9897	-0.0028
4	1	4	3	0	3	3362.8951	-0.0039
5	1	5	4	0	4	3737.8854	-0.0023
6	1	6	5	0	5	4102.6253	0.0009
7	1	7	6	0	6	4457.9554	0.0000
8	1	8	7	0	7	4804.9024	-0.0002
9	1	9	8	0	8	5144.6559	0.0066
10	1	10	9	0	9	5478.5252	0.0053
11	1	11	10	0	10	5807.9525	0.0029
12	1	12	11	0	11	6134.4502	0.0073
13	1	13	12	0	12	6459.5247	0.0017
15	1	15	14	0	14	7111.2823	-0.0088
9	0	9	8	1	8	2501.1666	0.0063
10	0	10	9	1	9	3008.0154	-0.0027
11	0	11	10	1	10	3516.8436	0.0122

13	0	13	12	1	12	4534.1144	-0.0114
14	0	14	13	1	13	5039.6872	-0.0041
16	0	16	15	1	15	6038.5087	0.0135
18	0	18	17	1	17	7015.1417	0.0042
19	0	19	18	1	18	7493.8923	-0.0097
3	2	2	2	1	1	6511.9462	-0.0059
5	2	4	4	1	3	7294.5032	-0.0165
6	2	5	5	1	4	7668.1392	-0.0057
12	2	10	12	1	11	4587.0717	-0.0216
11	2	9	11	1	10	4656.1886	-0.0085
10	2	8	10	1	9	4729.6561	-0.0075
9	2	7	9	1	8	4805.0787	0.0069
8	2	6	8	1	7	4880.1053	0.0107
7	2	5	7	1	6	4952.5536	0.0031
6	2	4	6	1	5	5020.4471	0.0039
4	2	3	4	1	4	5367.9174	0.0068
6	2	5	6	1	6	5498.9299	0.0038
7	2	6	7	1	7	5582.6645	0.0057
8	2	7	8	1	8	5678.6711	0.0008
9	2	8	9	1	9	5787.0678	0.0092
10	2	9	10	1	10	5907.9182	0.0015

Table S57. Experimental transition frequencies (v/MHz) together with the corresponding observed - calculated differences (Δv/MHz) for the parent (PA-FA)||(FA-FA) complex.

J'	K_a'	K_c'	J	K_a	K_c	v/MHz	Δv/MHz
3	0	3	2	0	2	2663.8949	0.0001
4	0	4	3	0	3	3507.3545	-0.0005
5	0	5	4	0	4	4335.8872	-0.0045
6	0	6	5	0	5	5160.8739	0.0039
7	0	7	6	0	6	5987.4930	-0.0025
8	0	8	7	0	7	6816.3600	-0.0076
9	0	9	8	0	8	7646.7599	-0.0003
3	1	3	2	1	2	2597.2665	0.0025
4	1	4	3	1	3	3450.9750	0.0013
5	1	5	4	1	4	4297.7814	-0.0032
6	1	6	5	1	5	5138.9510	-0.0017
7	1	7	6	1	6	5976.1444	-0.0022
8	1	8	7	1	7	6810.8606	-0.0130
9	1	9	8	1	8	7644.2154	-0.0025
3	1	2	2	1	1	2812.2442	0.0003
4	1	3	3	1	2	3730.9534	0.0002
5	1	4	4	1	3	4629.7929	0.0009
6	1	5	5	1	4	5501.9245	0.0059
7	1	6	6	1	5	6346.0893	0.0034
8	1	7	7	1	6	7170.7309	0.0037
9	1	8	8	1	7	7988.7619	0.0122
3	2	2	2	2	1	2712.9337	-0.0006
3	2	1	2	2	0	2761.9773	0.0063
4	2	3	3	2	2	3607.1268	0.0002
4	2	2	3	2	1	3716.8595	0.0022
5	2	4	4	2	3	4492.9566	0.0037
5	2	3	4	2	2	4677.4747	0.0010
6	2	5	5	2	4	5369.0436	-0.0008
6	2	4	5	2	3	5629.1615	0.0016
7	2	6	6	2	5	6234.8723	-0.0034
7	2	5	6	2	4	6562.1412	-0.0031
8	2	7	7	2	6	7090.9644	-0.0013
8	2	6	7	2	5	7469.8150	-0.0005
9	2	8	8	2	7	7938.7882	0.0107
4	3	2	3	3	1	3639.1919	0.0004
4	3	1	3	3	0	3647.7981	0.0009
5	3	3	4	3	2	4552.6218	0.0005
5	3	2	4	3	1	4581.3180	0.0000
6	3	4	5	3	3	5463.5214	0.0034

6	3	3	5	3	2	5533.2726	0.0005
7	3	5	6	3	4	6368.6705	0.0034
7	3	4	6	3	3	6503.0417	-0.0031
8	3	6	7	3	5	7265.2221	0.0051
8	3	5	7	3	4	7479.9511	-0.0102
5	4	2	4	4	1	4550.0763	0.0021
5	4	1	4	4	0	4551.1582	-0.0041
6	4	3	5	4	2	5468.1186	-0.0056
6	4	2	5	4	1	5472.8892	-0.0070
7	4	4	6	4	3	6388.5425	0.0022
7	4	3	6	4	2	6403.7550	-0.0028
8	4	5	7	4	4	7309.4778	0.0011
8	4	4	7	4	3	7348.3904	0.0021
7	5	3	6	5	2	6376.2387	0.0034
7	5	2	6	5	1	6376.8587	-0.0030
8	5	4	7	5	3	7297.3916	-0.0023
8	5	3	7	5	2	7299.8294	-0.0020
7	6	2	6	6	1	6366.6667	-0.0014
7	6	1	6	6	0	6366.6667	-0.0014
3	1	3	2	0	2	2801.6920	0.0004
4	1	4	3	0	3	3588.7699	-0.0006
5	1	5	4	0	4	4379.1942	-0.0059
6	1	6	5	0	5	5182.2548	-0.0065
7	1	7	6	0	6	5997.5403	0.0022
8	1	8	7	0	7	6820.9132	-0.0028
9	1	9	8	0	8	7648.7649	-0.0015
3	0	3	2	1	2	2459.4671	0.0000
4	0	4	3	1	3	3369.5741	0.0159
5	0	5	4	1	4	4254.4778	0.0016
6	0	6	5	1	5	5117.5603	-0.0011
7	0	7	6	1	6	5966.1004	-0.0038
8	0	8	7	1	7	6806.3263	0.0011
9	0	9	8	1	8	7642.2095	-0.0023
2	2	0	1	1	1	2756.8901	0.0033
3	2	1	2	1	2	3782.3873	0.0069
4	2	2	3	1	3	4901.9809	0.0072
5	1	4	4	2	3	4061.8602	0.0030
6	1	5	5	2	4	5070.8241	0.0011
7	1	6	6	2	5	6047.8676	0.0032
9	1	8	8	2	7	7881.4995	-0.0001
2	2	1	1	1	0	2671.8484	-0.0008
3	2	2	2	1	1	3504.0065	0.0012
4	2	3	3	1	2	4298.8874	-0.0005

5	2	4	4	1	3	5060.8870	-0.0006
6	2	5	5	1	4	5800.1399	-0.0002
7	2	6	6	1	5	6533.1086	0.0113
8	2	7	7	1	6	7277.9827	0.0054
3	3	1	2	2	0	4206.3020	-0.0026
4	3	2	3	2	1	5083.5230	-0.0019
5	3	3	4	2	2	5919.2815	-0.0075
6	3	4	5	2	3	6705.3245	-0.0088
7	3	5	6	2	4	7444.8477	0.0071
3	3	0	2	2	1	4220.6551	-0.0018
4	3	1	3	2	2	5155.5169	-0.0027
5	3	2	4	2	3	6129.7106	-0.0005
6	3	3	5	2	4	7170.0269	-0.0034
6	2	4	5	3	3	4387.3460	0.0014
7	2	5	6	3	4	5485.9674	-0.0035
4	4	0	3	3	1	5718.3283	0.0028

Table S58. Experimental transition frequencies (v/MHz) together with the corresponding observed - calculated differences (Δv/MHz) for the parent (PA-FA)||(PA-FA) complex.

J'	K_a'	K_c'	J	K_a	K_c	v/MHz	Δv/MHz
3	0	3	2	0	2	2149.6443	0.0049
4	0	4	3	0	3	2812.1264	0.0045
5	0	5	4	0	4	3470.7059	0.0046
6	0	6	5	0	5	4132.1900	0.0037
7	0	7	6	0	6	4796.0701	-0.0012
8	0	8	7	0	7	5461.0935	-0.0093
9	0	9	8	0	8	6126.5716	-0.0236
3	1	3	2	1	2	2101.1294	0.0047
3	1	2	2	1	1	2329.3219	-0.0022
4	1	4	3	1	3	2783.0676	0.0062
4	1	3	3	1	2	3068.5581	0.0003
5	1	5	4	1	4	3457.2826	0.0052
5	1	4	4	1	3	3770.2281	0.0017
6	1	6	5	1	5	4126.8511	0.0037
6	1	5	5	1	4	4436.8319	0.0035
7	1	7	6	1	6	4794.1226	-0.0020
7	1	6	6	1	5	5088.6221	0.0043
8	1	8	7	1	7	5460.4217	-0.0094
8	1	7	7	1	6	5742.2291	0.0072
9	1	9	8	1	8	6126.3501	-0.0221
9	1	8	8	1	7	6401.1701	0.0066
0	1	9	9	1	8	7063.6751	0.0046
1	1	10	10	1	9	7727.9322	-0.0054
3	2	2	2	2	1	2230.0020	-0.0034
3	2	1	2	2	0	2310.3754	-0.0099
4	2	3	3	2	2	2955.3081	0.0050
4	2	2	3	2	1	3115.8425	-0.0023
5	2	4	4	2	3	3666.6062	0.0065
5	2	3	4	2	2	3907.2576	-0.0028
6	2	5	5	2	4	4363.4371	0.0091
6	2	4	5	2	3	4669.0593	-0.0038
7	2	6	6	2	5	5047.7663	0.0102
7	2	5	6	2	4	5390.7335	-0.0038
8	2	7	7	2	6	5723.1514	0.0098
8	2	6	7	2	5	6069.0605	-0.0059
9	2	8	8	2	7	6393.2139	0.0095
9	2	7	8	2	6	6718.6983	-0.0050
0	2	9	9	2	8	7060.5940	0.0082
0	2	8	9	2	7	7362.8965	-0.0005

1	2	10	10	2	9	7726.8005	-0.0016
5	3	3	4	3	2	3760.1475	0.0017
5	3	2	4	3	1	3834.7184	-0.0034
6	3	4	5	3	3	4501.8849	0.0042
6	3	3	5	3	2	4656.0853	-0.0010
7	3	5	6	3	4	5229.3169	0.0057
7	3	4	6	3	3	5472.4605	-0.0014
8	3	6	7	3	5	5940.4057	0.0088
8	3	5	7	3	4	6260.2911	-0.0048
9	3	7	8	3	6	6635.8606	0.0144
9	3	6	8	3	5	7006.1781	-0.0126
0	3	8	9	3	7	7318.6695	0.0141
0	3	7	9	3	6	7703.4406	-0.0191
5	4	2	4	4	1	3766.6606	-0.0130
5	4	1	4	4	0	3772.0000	-0.0057
6	4	3	5	4	2	4530.5176	-0.0047
6	4	2	5	4	1	4552.7041	-0.0124
7	4	4	6	4	3	5292.6921	0.0029
7	4	3	6	4	2	5356.8400	-0.0025
8	4	5	7	4	4	6047.3322	0.0038
8	4	4	7	4	3	6186.0211	0.0018
9	4	6	8	4	5	6788.9158	0.0048
9	4	5	8	4	4	7022.5591	0.0055
0	4	7	9	4	6	7513.8357	0.0057
8	5	3	7	5	2	6073.7985	-0.0161
9	5	5	8	5	4	6825.6004	-0.0008
9	5	4	8	5	3	6878.0198	0.0003
0	5	6	9	5	5	7590.7523	0.0010
0	5	5	9	5	4	7708.8629	0.0146

Table S59. Equilibrium (r_e) and substitution (r_s) coordinates of the FA-FA···PA complex.

Atom	Coordinates (Å)					
	r_e			$\|r_s\|^a$		
	x	y	z	x	y	z
C	3.920970	-0.968007	0.000000	3.9450(5)	0.981(2)	
O	3.990489	0.247238	0.000000			
O	2.822075	-1.680239	0.000000			
H	4.816336	-1.598761	0.000000	4.8429(4)	1.594(1)	
H	2.018587	-1.086199	0.000000	2.089(1)	1.236(1)	
C	0.792358	1.200116	0.000000	0.779(2)	1.212(1)	
O	0.701338	-0.027969	0.000000			
O	1.906792	1.864034	0.000000			
H	-0.103469	1.825643	0.000000	0.16(1)*i	1.777(1)	
H	2.707074	1.250379	0.000000	2.7374(7)	1.435(1)	
C	-2.692578	0.058181	0.000000	2.6804(7)	0.07(2)*i	
O	-2.339332	1.216820	0.000000			
O	-1.870150	-0.986678	0.000000			
H	-0.936134	-0.656124	0.000000	0.954(2)	0.696 (2)	
C	-4.085349	-0.335735	0.000000	4.082(2)	0.349(4)	
C	-5.252408	-0.609604	0.000000	5.2520(4)	0.634(2)	
H	-6.287185	-0.852950	0.000000			

[a]The substitution (r_s) coordinates were obtained based on the assumption that the cluster is planar.

Table S60. Effective ground-state (r_0) coordinates of the FA-FA···PA complex.

Atom	Coordinates (Å)					
	A	dA	B	dB	C	dC
C	3.94857	0.00295	0.97309	0.00898	0.00000	0.00000
O	3.96516	0.01031	-0.30029	0.00534	0.00000	0.00000
O	2.88628	0.00243	1.72784	0.00373	0.00000	0.00000
H	4.87088	0.00802	1.56374	0.00847	0.00000	0.00000
H	2.08386	0.00222	1.13236	0.00392	0.00000	0.00000
C	0.81716	0.00584	-1.19100	0.00109	0.00000	0.00000
O	0.72395	0.00480	0.03692	0.00103	0.00000	0.00000
O	1.93278	0.00641	-1.85293	0.00194	0.00000	0.00000
H	-0.07755	0.00638	-1.81812	0.00075	0.00000	0.00000
H	2.73197	0.00588	-1.23785	0.00261	0.00000	0.00000
C	-2.69199	0.00117	-0.04733	0.00022	0.00000	0.00000
O	-2.33668	0.00036	-1.20534	0.00010	0.00000	0.00000
O	-1.87143	0.00208	0.99899	0.00051	0.00000	0.00000
H	-0.93683	0.00179	0.67011	0.00134	0.00000	0.00000
C	-4.08546	0.00150	0.34410	0.00146	0.00000	0.00000
C	-5.25301	0.00174	0.61589	0.00250	0.00000	0.00000
H	-6.28822	0.00195	0.85739	0.00342	0.00000	0.00000

Table S61. Semi-experimental equilibrium (r_e^{SE}) coordinates of the FA-FA···PA complex.

Atom	Coordinates (Å)					
	A	dA	B	dB	C	dC
C	3.92670	0.04933	1.00887	0.05813	0.00000	0.00000
O	4.01689	0.02733	-0.20501	0.06077	0.00000	0.00000
O	2.81585	0.06882	1.70231	0.02929	0.00000	0.00000
H	4.81121	0.06743	1.65477	0.08469	0.00000	0.00000
H	2.02259	0.05163	1.09469	0.02422	0.00000	0.00000
C	0.81101	0.07966	-1.23178	0.01705	0.00000	0.00000
O	0.70639	0.05259	0.00037	0.04963	0.00000	0.00000
O	1.93658	0.09964	-1.87664	0.03153	0.00000	0.00000
H	-0.07405	0.09951	-1.87245	0.03922	0.00000	0.00000
H	2.72630	0.08021	-1.24946	0.05494	0.00000	0.00000
C	-2.67067	0.02875	-0.04368	0.04042	0.00000	0.00000
O	-2.33768	0.07383	-1.20829	0.05312	0.00000	0.00000
O	-1.87522	0.08198	0.93146	0.19546	0.00000	0.00000
H	-0.93964	0.06835	0.60536	0.14169	0.00000	0.00000
C	-4.05637	0.03248	0.37445	0.05906	0.00000	0.00000
C	-5.21848	0.04259	0.66861	0.11616	0.00000	0.00000
H	-6.24886	0.05387	0.92995	0.16973	0.00000	0.00000

Table S62. Equilibrium (r_e) and substitution (r_s) coordinates of the PA-FA···FA complex.

Atom	Coordinates (Å)					
	r_e			$\|r_s\|$		
	x	y	z	x	y	z
C	4.157812	-0.792373	0.000000	4.179(1)	0.811(6)	
O	4.123567	0.412852	0.000000			
O	3.105281	-1.599893	0.000000			
H	5.088944	-1.374192	0.000000	5.1239(4)	1.366(1)	
H	2.276349	-1.058875	0.000000	2.2223(8)	1.139(1)	
C	1.080368	1.192193	0.000000	1.077(5)	1.206(4)	
O	0.840662	-0.015531	0.000000			
O	0.182426	2.128922	0.000000			
H	2.110728	1.556146	0.000000	2.1541(8)	1.479(1)	
H	-0.750263	1.747979	0.000000	1.100(5)	1.854(3)	
C	-2.513425	-0.082750	0.000000	2.5018(7)	0.05(3)*i	
O	-2.250299	1.111976	0.000000			
O	-1.625662	-1.052433	0.000000			
H	-0.700569	-0.672385	0.000000	0.04(4)*i	0.006(272)*i[b]	
C	-3.876508	-0.552839	0.000000	3.8730(4)	0.567(3)	
C	-5.021728	-0.905613	0.000000	5.0198(3)	0.932(2)	
H	-6.037779	-1.219547	0.000000			

[a]The substitution (r_s) coordinates were obtained based on the assumption that the cluster is planar.

[b]The large uncertainty arises due to the atom being located near the coordinate origin.

Table S63. Effective ground-state (r_0) coordinates of the PA-FA···FA complex.

Atom	Coordinates (Å)					
	A	dA	B	dB	C	dC
C	4.18599	0.00286	-0.77858	0.01023	0.00000	0.00000
O	4.07805	0.03259	0.42229	0.00743	0.00000	0.00000
O	3.18459	0.04089	-1.64923	0.00519	0.00000	0.00000
H	5.15098	0.01493	-1.30233	0.03633	0.00000	0.00000
H	2.35572	0.03774	-1.10811	0.00845	0.00000	0.00000
C	1.10589	0.00663	1.20137	0.00199	0.00000	0.00000
O	0.86249	0.00244	-0.02423	0.00455	0.00000	0.00000
O	0.20627	0.00766	2.13648	0.00235	0.00000	0.00000
H	2.13560	0.00703	1.56717	0.00225	0.00000	0.00000
H	-0.72574	0.00723	1.75385	0.00309	0.00000	0.00000
C	-2.51409	0.00046	-0.09202	0.00286	0.00000	0.00000
O	-2.25312	0.00180	1.10318	0.00256	0.00000	0.00000
O	-1.62458	0.00088	-1.06010	0.00184	0.00000	0.00000
H	-0.70018	0.00048	-0.67839	0.00090	0.00000	0.00000
C	-3.87633	0.00039	-0.56457	0.00446	0.00000	0.00000
C	-5.02091	0.00072	-0.91940	0.00582	0.00000	0.00000
H	-6.03639	0.00108	-1.23517	0.00703	0.00000	0.00000

Table S64. Semi-experimental equilibrium (r_e^{SE}) coordinates of the PA-FA···FA complex.

Atom	Coordinates (Å)					
	A	dA	B	dB	C	dC
C	4.16854	0.00501	-0.78443	0.01615	0.00000	0.00000
O	4.04961	0.06012	0.41540	0.01071	0.00000	0.00000
O	3.20227	0.07487	-1.64020	0.00758	0.00000	0.00000
H	5.13829	0.02484	-1.29931	0.06326	0.00000	0.00000
H	2.37172	0.06893	-1.10168	0.01267	0.00000	0.00000
C	1.09686	0.01119	1.20854	0.00297	0.00000	0.00000
O	0.85861	0.00525	-0.01102	0.00886	0.00000	0.00000
O	0.19313	0.01333	2.13970	0.00324	0.00000	0.00000
H	2.12494	0.01204	1.57886	0.00431	0.00000	0.00000
H	-0.73718	0.01244	1.75299	0.00473	0.00000	0.00000
C	-2.50380	0.00094	-0.09492	0.00540	0.00000	0.00000
O	-2.24808	0.00375	1.10141	0.00478	0.00000	0.00000
O	-1.61006	0.00161	-1.05909	0.00328	0.00000	0.00000
H	-0.68733	0.00077	-0.67333	0.00130	0.00000	0.00000
C	-3.86395	0.00060	-0.57344	0.00867	0.00000	0.00000
C	-5.00697	0.00132	-0.93330	0.01144	0.00000	0.00000
H	-6.02105	0.00207	-1.25351	0.01390	0.00000	0.00000

Table S65. Equilibrium (r_e) and substitution (r_s) coordinates of the PA-FA···PA complex.

Atom	Coordinates (Å)					
	r_e			$\|r_s\|$[a]		
	x	y	z	x	y	z
C	-5.872763	-1.147790	0.000000			
H	-6.864626	-1.530156	0.000000			
C	-4.753987	-0.717204	0.000000			
C	-3.428098	-0.136648	0.000000			
O	-3.236663	1.059472	0.000000			
O	-2.470640	-1.059296	0.000000			
H	-1.590665	-0.604341	0.000000	1.522(1)	0.740(2)	
C	-0.135210	1.476788	0.000000			
H	-1.108773	1.972511	0.000000	1.280(1)	1.8566(8)	
O	-0.055550	0.247864	0.000000			
O	0.877439	2.287429	0.000000			
H	1.752737	1.787737	0.000000	1.887(1)	1.9455(8)	
C	5.645052	-1.393073	0.000000			
H	6.612345	-1.835001	0.000000			
C	4.554674	-0.896023	0.000000			
C	3.263453	-0.254595	0.000000			
O	3.156056	0.964075	0.000000			
O	2.258378	-1.102209	0.000000			
H	1.390343	-0.606690	0.000000	1.650(1)	0.817(2)	

[a]The substitution (r_s) coordinates were obtained based on the assumption that the cluster is planar.

Table S66. Equilibrium (r_e) coordinates of the (PA-FA)||(FA-FA) complex.

Atom	Coordinates (Å)		
	x	y	z
C	-0.196946	-1.946510	1.178474
O	-0.376373	-2.013159	-0.025879
O	0.888867	-1.532942	1.775318
H	-0.971222	-2.243213	1.892941
H	1.577168	-1.238876	1.105125
C	-1.880758	0.677510	-0.131039
O	-1.418313	0.817813	0.995593
O	-1.282536	1.026526	-1.242721
H	-0.373657	1.413738	-1.049714
C	-3.182729	0.091562	-0.334363
C	-4.268003	-0.400221	-0.461486
H	-5.230216	-0.836965	-0.578991
O	2.651379	-0.704518	-0.031387
C	2.450502	-0.742705	-1.234239
O	1.388920	-1.217007	-1.826379
H	0.715030	-1.543087	-1.154039
H	3.184529	-0.361848	-1.952024
O	1.115138	2.005698	-0.731620
C	1.574691	2.137973	0.390727
H	2.584980	2.524819	0.557095
O	0.967829	1.855294	1.511244
H	0.053564	1.478154	1.337791

Table S67. Equilibrium (r_e) coordinates of the (PA-FA)||(PA-FA) complex.

Atom	Coordinates (Å)		
	x	y	z
C	3.415795	1.964687	1.071866
H	4.402823	2.072655	1.451648
C	2.301321	1.842321	0.649491
C	0.979023	1.670895	0.101319
O	0.812535	1.202859	-1.018825
O	0.021248	2.059844	0.907573
H	-0.878761	1.903827	0.486424
C	-2.494591	1.127241	-1.305210
H	-3.491285	0.931958	-1.713803
O	-2.340049	1.604548	-0.192955
O	-1.549239	0.792272	-2.139895
H	-0.642629	0.956579	-1.738922
C	3.390940	-2.046370	-1.065734
H	4.375727	-2.175626	-1.444677
C	2.279001	-1.899836	-0.644357
C	0.960121	-1.700577	-0.097299
O	0.802883	-1.243171	1.028566
O	-0.005224	-2.053733	-0.910909
H	-0.901945	-1.880091	-0.489931
C	-2.502215	-1.071173	1.301532
H	-3.494239	-0.848987	1.707699
O	-2.357685	-1.550571	0.188866
O	-1.550223	-0.763803	2.139364
H	-0.647304	-0.954373	1.741438

References

1. Schmitz, D., Alvin Shubert, V., Betz, T. & Schnell, M. Multi-resonance effects within a single chirp in broadband rotational spectroscopy: The rapid adiabatic passage regime for benzonitrile. *J. Mol. Spectrosc.* **280**, 77–84 (2012).

2. Li, W. *et al.* Evolution of Solute–Water Interactions in the Benzaldehyde-$(H_2O)_{1-6}$ Clusters by Rotational Spectroscopy. *J. Am. Chem. Soc.* **145**, 4119–4128 (2023).

3. Plusquellic, D. F. JB95 Spectral Fitting Program. National Institute of Standards and Technology (NIST), Gaithersburg, MD.

4. Kisiel, Z. *et al.* Rotational spectrum of *trans–trans* diethyl ether in the ground and three excited vibrational states. *J. Mol. Spectrosc.* **233**, 231–243 (2005).

5. Costain, C. C. Determination of Molecular Structures from Ground State Rotational Constants. *J. Chem. Phys.* **29**, 864–874 (1958).

6. Pracht, P., Bohle, F. & Grimme, S. Automated exploration of the low-energy chemical space with fast quantum chemical methods. *Phys. Chem. Chem. Phys.* **22**, 7169–7192 (2020).

7. Lee, C., Yang, W. & Parr, R. G. Development of the Colle-Salvetti correlation-energy formula into a functional of the electron density. *Phys. Rev. B* **37**, 785–789 (1988).

8. Becke, A. D. Density-functional thermochemistry. III. The role of exact exchange. *J. Chem. Phys.* **98**, 5648–5652 (1993).

9. Weigend, F. & Ahlrichs, R. Balanced basis sets of split valence, triple zeta valence and quadruple zeta valence quality for H to R_n: Design and assessment of accuracy. *Phys. Chem. Chem. Phys.* **7**, 3297 (2005).

10. Caldeweyher, E. *et al.* A generally applicable atomic-charge dependent London dispersion correction. *J. Chem. Phys.* **150**, 154122 (2019).

11. Neese, F. The ORCA program system. *WIREs Comput. Mol. Sci.* **2**, 73–78 (2012).

12. Grimme, S., Ehrlich, S. & Goerigk, L. Effect of the damping function in dispersion corrected density functional theory. *J. Comput. Chem.* **32**, 1456–1465 (2011).

13. Grimme, S., Antony, J., Ehrlich, S. & Krieg, H. A consistent and accurate *ab initio* parametrization of density functional dispersion correction (DFT-D) for the 94 elements H-Pu. *J. Chem. Phys.* **132**, 154104 (2010).

14. Santra, G., Sylvetsky, N. & Martin, J. M. L. Minimally Empirical Double-Hybrid Functionals Trained against the GMTKN55 Database: revDSD-PBEP86-D4, revDOD-PBE-D4, and DOD-SCAN-D4. *J. Phys. Chem. A* **123**, 5129–5143 (2019).

15. Grimme, S. Semiempirical hybrid density functional with perturbative second-order correlation. *J. Chem. Phys.* **124**, 034108 (2006).

16. Papajak, E., Zheng, J., Xu, X., Leverentz, H. R. & Truhlar, D. G. Perspectives on Basis Sets Beautiful: Seasonal Plantings of Diffuse Basis Functions. *J. Chem. Theory Comput.* **7**, 3027–3034 (2011).

17. Møller, Chr. & Plesset, M. S. Note on an Approximation Treatment for Many-Electron Systems. *Phys. Rev.* **46**, 618–622 (1934).

18. Dunning, T. H. Gaussian basis sets for use in correlated molecular calculations. I. The atoms boron through neon and hydrogen. *J. Chem. Phys.* **90**, 1007–1023 (1989).

19. Kendall, R. A., Dunning, T. H. & Harrison, R. J. Electron affinities of the first-row atoms revisited. Systematic basis sets and wave functions. *J. Chem. Phys.* **96**, 6796–6806 (1992).

20. Frisch, M. J. *et al.* Gaussian 09, revision D. 01. *Gaussian Inc. Wallingford CT* (2009).

21. Frisch, M. J. *et al.* Gaussian 16, revision A. 03. *Gaussian Inc. Wallingford CT* (2016).

22. Jeziorski, B., Moszynski, R. & Szalewicz, K. Perturbation Theory Approach to Intermolecular Potential Energy Surfaces of van der Waals Complexes. *Chem. Rev.* **94**, 1887–1930 (1994).

23. Turney, J. M. *et al.* Psi4: an open-source *ab initio* electronic structure program. *WIREs Comput. Mol. Sci.* **2**, 556–565 (2012).

24. Schmider, H. L. & Becke, A. D. Chemical content of the kinetic energy density. *J. Mol. Struct. THEOCHEM* **527**, 51–61 (2000).

25. Lu, T. & Chen, Q. A simple method of identifying π orbitals for non-planar systems and a protocol of studying π electronic structure. *Theor. Chem. Acc.* **139**, 25 (2020).

26. Lu, T. & Chen, F. Multiwfn: A multifunctional wavefunction analyzer. *J Comput Chem* **33**, 580–592 (2012).

27. Humphrey, W., Dalke, A. & Schulten, K. VMD: Visual molecular dynamics. *J. Mol. Graph.* **14**, 33–38 (1996).

28. Richard, R. M. & Herbert, J. M. A generalized many-body expansion and a unified view of fragment-based methods in electronic structure theory. *J. Chem. Phys.* **137**, 064113 (2012).

29. Pulay, P., Meyer, W. & Boggs, J. E. Cubic force constants and equilibrium geometry of methane from Hartree–Fock and correlated wavefunctions. *J. Chem. Phys.* **68**, 5077–5085 (1978).

30. Kisiel, Z. Least-squares mass-dependence molecular structures for selected weakly bound intermolecular clusters. *J. Chem. Phys.* **218**, 58–67 (2003).
